# Ir-Catalyzed Chemo-, Regio-, and Enantioselective Allylic Enolization of 6,6-Dimethyl-3-((trimethylsilyl)oxy)cyclohex-2-en-1-one Involving Keto-Enol Isomerization

**DOI:** 10.3390/molecules27206981

**Published:** 2022-10-17

**Authors:** Xiao-Lin Wang, Ji-Teng Chen, Sheng-Cai Zheng, Xiao-Ming Zhao

**Affiliations:** School of Chemical Technology and Engineering, Tongji University, 1239 Siping Road, Shanghai 200092, China

**Keywords:** enolization, chemoselectivity, regioselectivity, enantioselectivity, fluorination

## Abstract

The utilization of 6,6-dimethyl-3-((trimethylsilyl)oxy)cyclohex-2-en-1-one made from an unsymmetrical 4,4-dimethylcyclohexane-1,3-dione in iridium-catalyzed allylic enolization involving keto-enol isomerization is accomplished under mild conditions. The chemoselectivity, regioselectivity, and enantioselectivity are facilitated by the quaternary carbon and adjusting the reaction conditions. This method provides the substituted 2-(but-3-en-2-yl)-3-hydroxy-6,6-dimethylcyclohex-2-en-1-ones in good to high yields with high level of chemo-, regio-, and enantioselectivities. The chiral carbon-fluorine bond formation is induced by an adjacent chiral carbon center of the allylated 3-hydroxy-6,6-dimethylcyclohex-2-en-1-one, as well.

## 1. Introduction

Enol is of great importance to synthetic chemistry since it could undergo various transformation [1,2]. As a result, new method for introducing enol moiety into organic compounds is highly desirable. To date, acyclic enol as a nucleophile was used in transition-metal catalyzed allylic substitution [3,4,5,6,7,8,9,10,11,12,13,14].

**Scheme 1:**
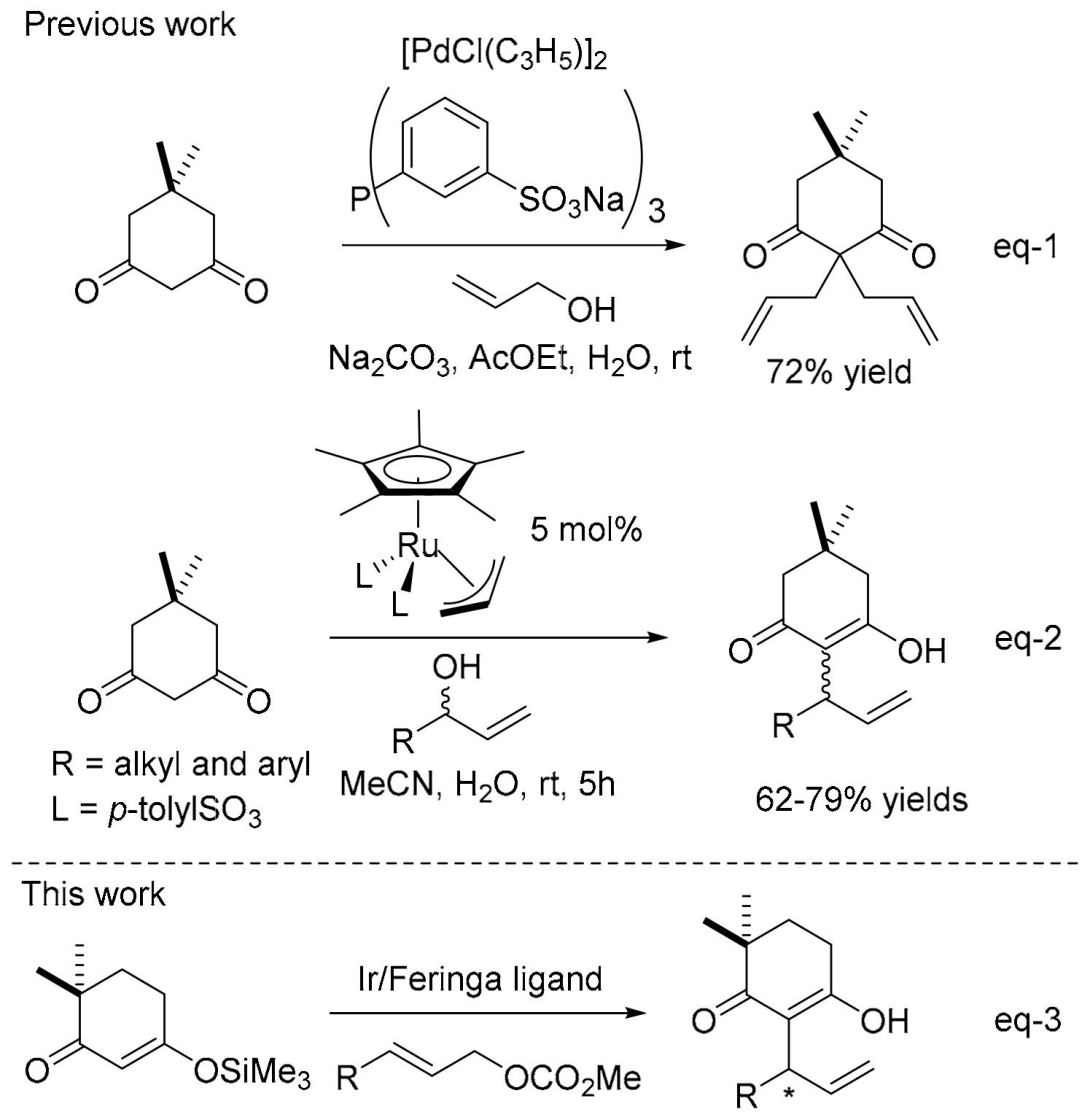
Cyclohexane-1,3-dione derivatives employed in allylic substitution under Pd, Ru, and Ir catalysis.

**Scheme 2:**
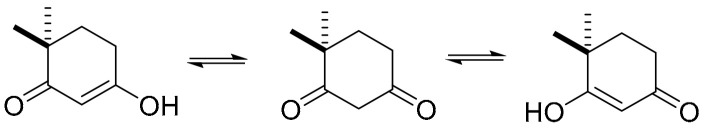
Keto-enol isomerization of 4,4-dimethylcyclohexane-1,3-dione.

Very few cyclic enol such as 5,5-dimethylcyclohexane-1,3-dione was used in allylic substitution under Pd [15,16,17,18] and Ru [19] catalysis (eq-1 and eq-2 in Scheme 1). Interestingly, the racemic allylated 3-hydroxy-5,5-dimethylcyclohex-2-en-1-one was observed in eq-2 of Scheme 1. Iridium-catalyzed allylic substitution has become a powerful tool for the synthesis of optically active compounds [20,21]. In general, 4,4-dimethylcyclohexane-1,3-dione that is structurally unsymmetrical could carry out keto-enol isomerization to form either 3-hydroxy-6,6-dimethylcyclohex-2-en-1-one or 3-hydroxy-4,4-dimethylcyclohex-2-en-1-one [3,22] (Scheme 2); thus, stereoselective introduction of cyclic enol moiety into allyl products is a changeling task. We speculated that unsymmetrical 3-hydroxy-4,4-dimethylcyclohex-2-en-1-one may undergo enantioselective allylic substitution under Ir catalysis to form two chiral centers. In this paper, we describe Ir-catalyzed enantioselective allylic enolization of 6,6-dimethyl-3-((trimethylsilyl)oxy)cyclohex-2-en-1-one involving keto-enol isomerization.

## 2. Results and Discussion

**Figure 1:**
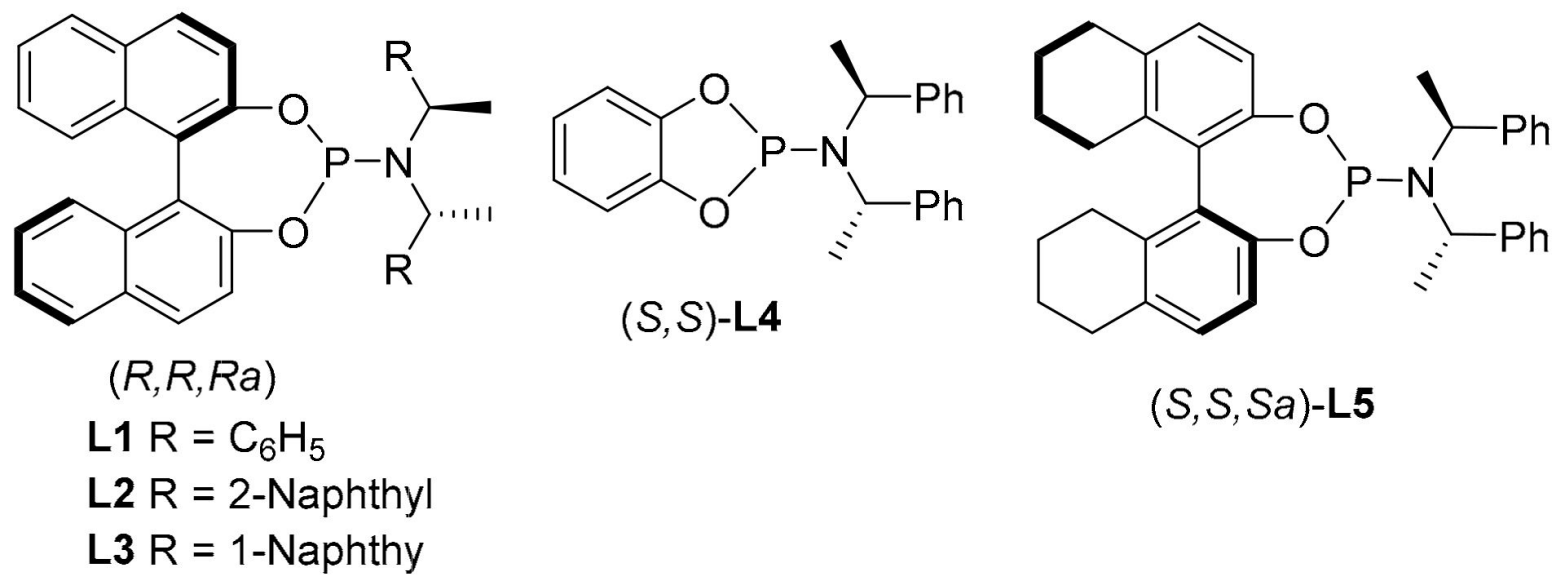
Ligands used in this reaction.

We initiated with a model reaction of 6,6-dimethyl-3-((trimethylsilyl)oxy)cyclohex-2-en-1-one **1a [22]**, which derived from 4,4-dimethylcyclohexane-1,3-dione **1b** and hexamethyldisilazane (HMDS), with (*E*)-cinnamyl methyl carbonate **2a** in the presence of CsF and an iridacycle [23] prepared from [Ir(COD)Cl]_2_ and Feringa’s ligand **L1 [24,25]** (Figure 1). When the reaction was conducted in dichloromethane (DCM) at −20 °C, the formation of 3-hydroxy-6,6-dimethyl-2-(1-phenylallyl)cyclohex-2-en-1-one **3a** was observed. Its enolated isomer, either 4,4-dimethyl-2-(1-phenylallyl)cyclohexane-1,3-dione **3a″** or 3-hydroxy-4,4-dimethyl-2-(1-phenylallyl) cyclohex-2-en-1-one **3a‴** was not found. These results suggested that it is difficult to form **3a‴** due to the repulsion between methyl and hydroxyl group. The structure of **3a** was established by 2D ^1^H NMR and X-ray analysis of **3j** (entry 1 and Figure S2; Supplementary Materials). Inspired by these results, various solvents were examined and they revealed that toluene is a proper solvent (entry 4); whereas other solvents such as DCM and acetonitrile offered **3a** in poor yields with good to high ee values (entries 1, 3, and 4); THF is not favorable for this reaction (entry 2). Base plays an important role in the allylic enolization, thus, a series of bases including CsF, CsCl, Cs_2_CO_3_, CsOH, K_2_CO_3_, and 1,8-diazabicyclo [5.4.0]-7-undecene (DBU) was tested and Cs_2_CO_3_ led to the better results than the others (entries 5–8); DBU is not effective for the reaction (entry 9). The reaction was performed at a temperature ranging from −20 °C to 35 °C, and we found that the reaction at 25 °C gave superior results (entries 6, 10–13). Notably, the incomplete conversion of **1a** led to a 75% yield (entry 12). Iridium salts such as [Ir(Cp*)Cl_2_]_2_ and Ir(COD)(acac) were surveyed, [Ir(Cp*)Cl_2_]_2_ was not suitable for this reaction (entry 14); Ir(COD)(acac) gave **3a** in a poor yield but with high ee value (entry 15). Ligand is crucial to Ir-catalyzed asymmetric allylic substitution [20,21]. Therefore, structurally varying ligands such as **L1**, **L2** [25], **L3** [26], **L4** [27], and **L5** [28] (Figure 1) were explored. **L1** led to a better ee value than **L2**; and **L2** gave a higher yield than that of **L1** (entry 12 vs. entry 16); **L3** gave a moderate ee value; both **L4** and **L5** are ineffective for this reaction (entries 16–19). Moreover, either 4,4-dimethylcyclohexane-1,3-dione **1b** or 2,2-dimethylcyclohexan-1-one **1c** was also tested under the optimal conditions and no corresponding product was detected; these results suggested that **1a** is requirable for this reaction (entry 20).

Having established the optimal reaction conditions shown in the entry 12 of Table 1, we further surveyed the scope of various allylic substrates **2**. When **2a** and substrates **2b**–**2c**, **2e**–**2h** having an electron-rich substituent (e.g., *p*-Et, *p*-*iso*-Propyl, *p*-MeO, *m*-Me, *m*-MeO, and *m*-EtO) on the phenyl ring were employed, the corresponding **3a**–**3c**, **3e**–**3h** were obtained in moderate to good yields with high regio- and enantioselectivities.

**Scheme 3:**
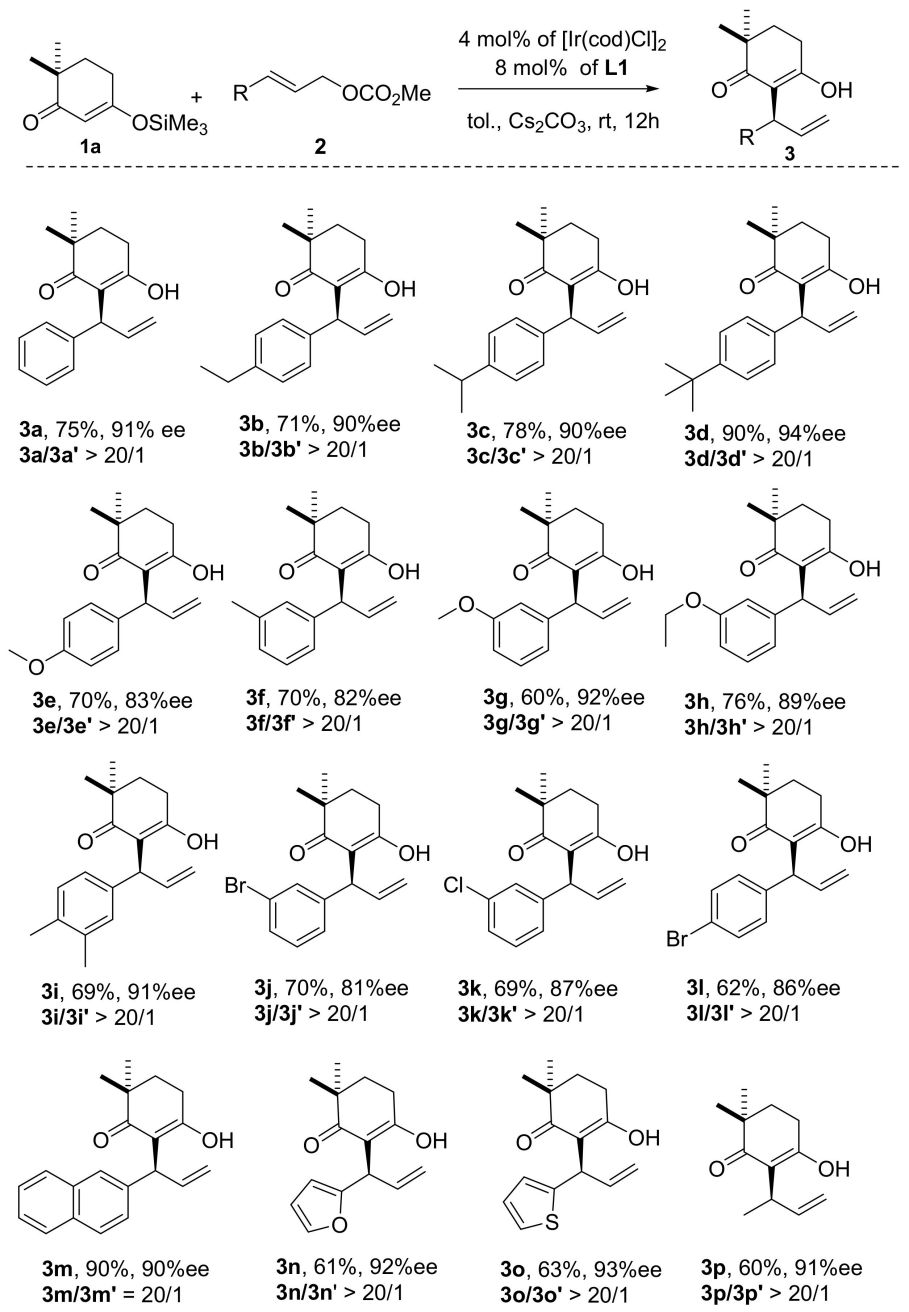
Scope of the allylic substrates **2**. Reaction conditions: [Ir(COD)Cl]_2_ (0.004 mmol), **L1** (0.008 mmol), **1a** (0.1 mmol), **2** (0.2 mmol), Cs_2_CO_3_ (0.12 mmol), and PhMe (1 mL). Yield referred to isolated yield and *ee* was determined by a chiral HPLC.

Interestingly, substrate **2d** bearing a bulky *tert*-butyl group and **2i** attaching 3,4-*di*-Me groups on the phenyl ring were used, **3d** and **3i** were also achieved in good to high yield with a high level of regio- and enantioselectivities. However, the substrates **2j**–**2l** containing the electron-poor substituent (e.g., *m*-Br, *m*-Cl, and *p*-Br) on the phenyl ring were utilized, the allyl products **3j**–**3l** were obtained in moderate to good yields with slight lowering ee values but with high regioselectivities. Naphthyl-substituted substrate **2m** and heteroaryl-substituted substrates (**2n** and **2o**) offered **3m**, **3n**–**3o** in good yield with high regio- and enantioselectivity. In particular, aliphatic-substituted substrate **2p** gave **3p** in a moderate yield with high regio- and enantioselectivity (Scheme 3).

**Scheme 4:**
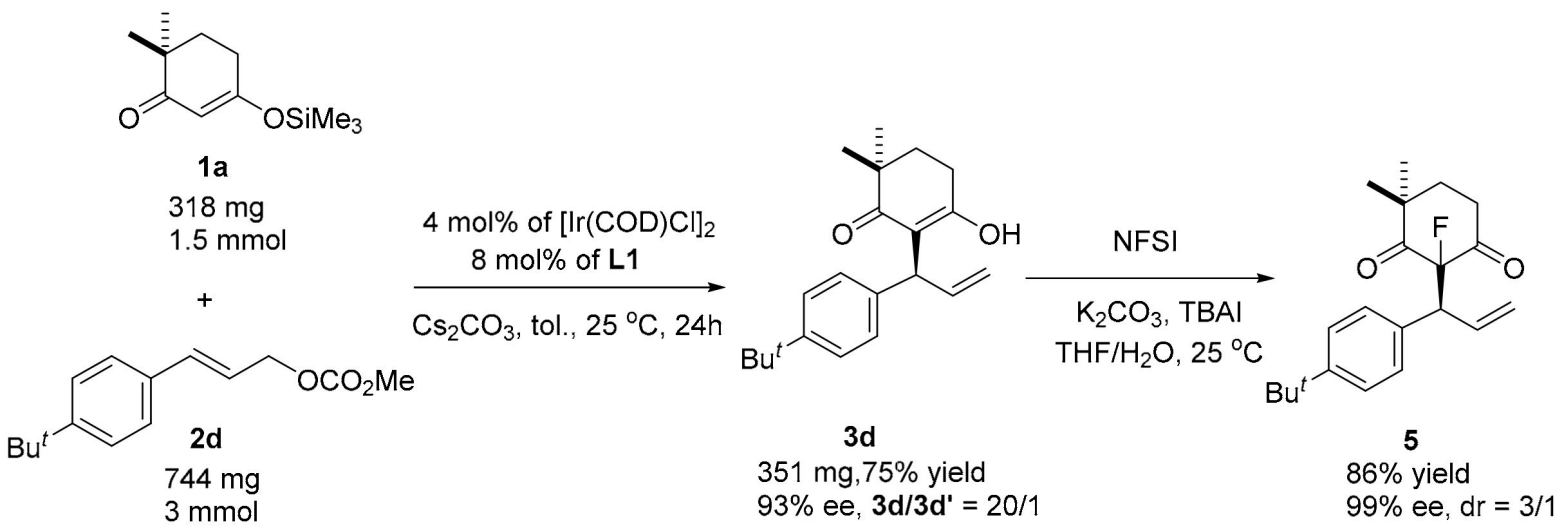
Scale-up synthesis of **3d** and its fluorination for the synthesis of **5** with a chiral C-F center.

The scale-up synthesis of enolated allyl product such as **3d** was conducted under the optimal conditions as shown in Scheme 3. **1a** (318 mg, 1.5 mmol) and the allylic substrate **2d** (744 mg, 3 mmol) were employed and **3d** (351 mg, 75% yield, 93% ee and **3d**/**3d′** = 20/1) was obtained (Scheme 4).

**Figure 2:**
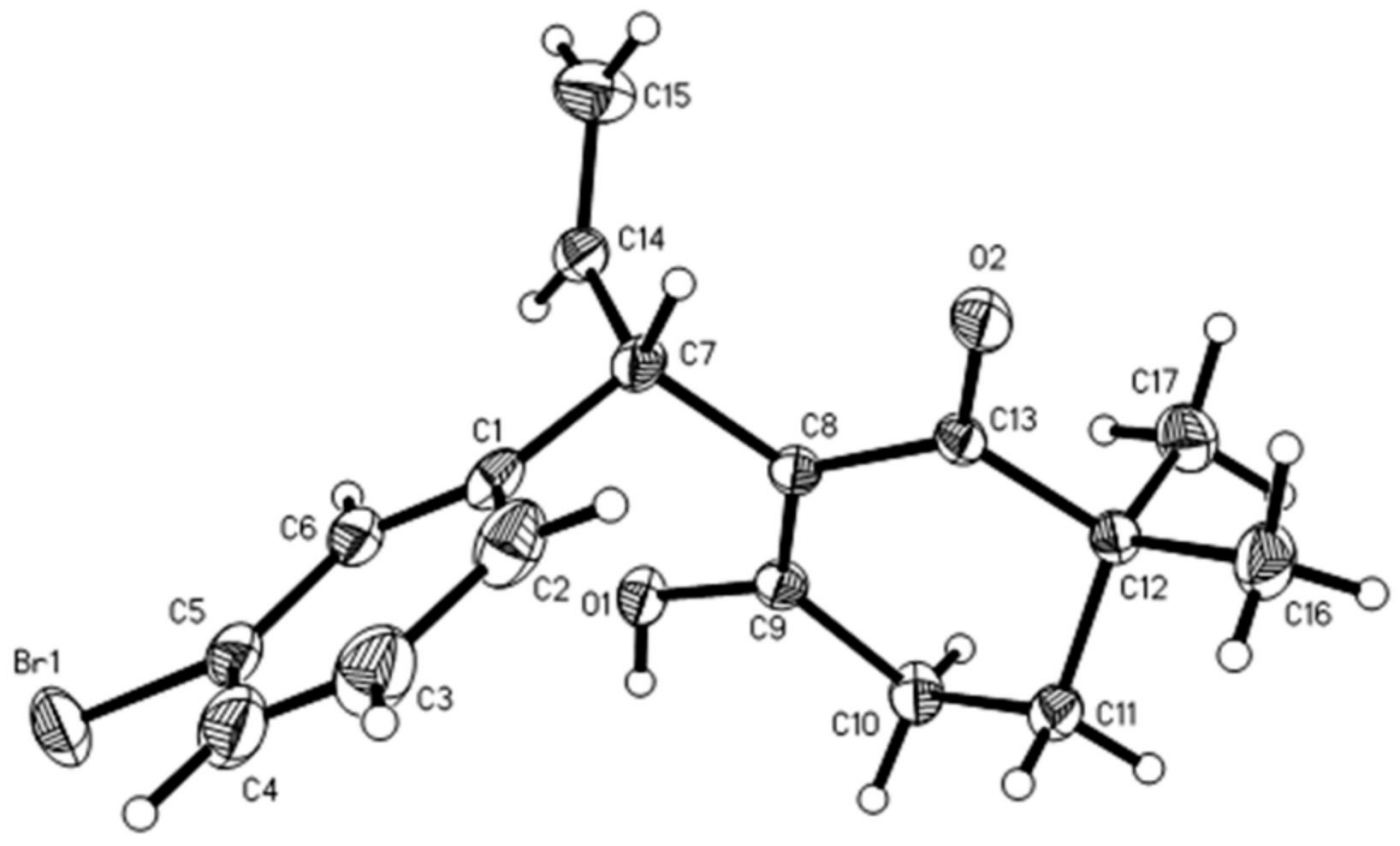
X-ray molecular structure of **3j**.

The application of the enolated product **3d** made by this method is shown in Scheme 3. The absolute configuration of **3j** was determined as *S* by its X-ray analysis [29] (Figure 2).

The enantioselective fluorination of **3d** was realized by a treatment of **3d** with *N*-fluorobenzenesulfonimide (NFSI, 0.15 mmol) in the presence of K_2_CO_3_ (0.25 mmol), tetrabutylammonium iodide (TBAI, 0.01 mmol) and THF/H_2_O (7/3) at room temperature and it gave the fluorinated **5** in an 86% yield with 99% ee and dr 3/1 (Scheme 4).

**Scheme 5:**
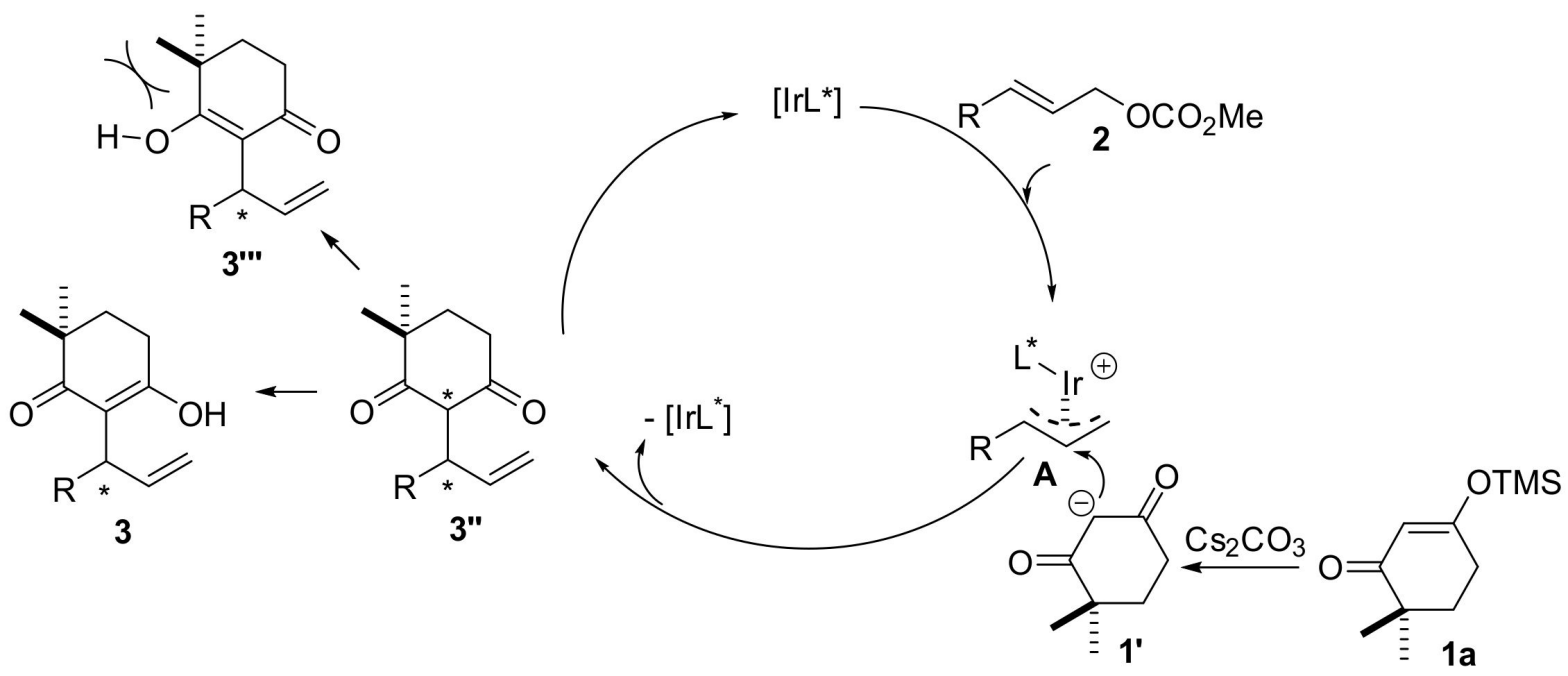
Possible mechanism.

For a possible mechanism, we outline that it begins by insertion of iridium into the allyl-oxygen bond in the presence of Cs_2_CO_3_ and toluene to yield an ionized (*π*-allyl)-Ir-complex (***Int A***). The treatment of **1** with Cs_2_CO_3_ liberates **1′**, the later attacks ***Int A*** to produce **3″**, which leads to enolization to provide **3**, regenerating the catalyst. The quaternary carbon center adjacent to the carbonyl group on the six-membered ring plays a significant role in the formation of enol **3** (or **3‴**) because of the resonance effect [19] and steric effect (Scheme 5).

## 3. Materials and Methods

### 3.1. Reagents and General Methods

All manipulations were carried out under the argon atmosphere using standard Schlenk techniques. All glassware were oven or flame dried immediately prior to use. All solvents were purified and dried according to standard methods prior to use, unless stated otherwise.

^1^H NMR spectra were obtained at 400 MHz or 600 MHz and recorded relative to the tetramethylsilane signal (0 ppm) or residual protio-solvent (7.26 ppm for CDCl_3_). ^13^C NMR spectra were obtained at 100 MHz or 150 MHz, and chemical shifts were recorded relative to the solvent resonance (CDCl_3_, 77.16 ppm). ^19^F NMR spectra were obtained at 376 MHz or 565 MHz. Data for NMR are recorded as follows: chemical shift (δ, ppm), multiplicity (s = singlet, d = doublet, t = triplet, m = multiplet or unresolved, br = broad singlet, coupling constant(s) in Hz, integration).

The phosphoramidite ligands [24,27,30], substituted allylic carbonates [31], were prepared according to the known procedures. Other chemicals were purchased from commercial suppliers and used without further purification, unless mentioned.

### 3.2. Synthetic Procedures

**General Procedure for the Synthesis of 3:** [Ir(COD)Cl]_2_ (0.004 mmol, 4 mol%), phosphoramidite ligand **L1** (0.008 mmol, 8 mol%) were dissolved in THF (0.5 mL) and *n*-propylamine (0.3 mL) in a dry Schlenk tube filled with argon. The reaction mixture was heated at 50 °C for 30 min and then the volatile solvents were removed under vacuum to give a yellow solid. After that, allylic carbonate **2** (0.20 mmol), cesium carbonate (Cs_2_CO_3_, 0.12 mmol), and toluene (1.0 mL) were added. In another dry Schlenk tube, 6,6-dimethyl-3-((trimethylsilyl) oxy) cyclohex-2-en-1-one **1a** was prepared from 4,4-dimethylcyclohexane-1,3-cyclohexanedione (0.10 mmol) and hexamethyldisilazane (HMDS) (0.15 mmol) in DCM (2.0 mL), under stirring for 2.5 h at room temperature, and the solvent was removed under vacuum to give a light-yellow liquid which was transferred through a syringe into the above mentioned Schlenk tube. The reaction was stirred at room temperature for 12 h. Then the mixture was washed with brine. After the organic phase was collected, the aqueous phase was extracted with DCM. The combined organic phase was dried over anhydrous Na_2_SO_4_ and concentrated on a rotary evaporator. The crude residue was purified by flash column chromatography (petroleum ether/ethyl acetate) to give the desired products **3**. 

**(*S*)-3-Hydroxy-6,6-dimethyl-2-(1-phenylallyl)cyclohex-2-en-1-one (3a),** white solid; **m.p.**: 103–105 °C; 75% yield (19.2 mg); **HPLC** *ee*: 91% [Daicel CHIRALPAK AD-H (0.46 cm × 25 cm); *n*-hexane/2-propanol = 90/10; flow rate = 1.0 mL/min; detection wavelength = 254 nm; t_R_ = 6.77 (minor), 8.22 (major) min]. **[α]_D_^20^** = +22.3 (c 1.0, CHCl_3_). **^1^H NMR** (600 MHz, CD_3_CN) δ 7.28–7.24 (m, 2H), 7.21 (d, *J* = 7.8 Hz, 2H), 7.17–7.13 (m, 1H), 6.49–6.43 (m, 1H), 5.11–5.05 (m, 2H), 4.88 (d, *J* = 7.8 Hz, 1H), 2.57–2.54 (m, 2H), 1.82 (t, *J* = 6.0 Hz, 2H), 1.06 (d, *J* = 6.0 Hz, 6H).**^13^C NMR** (150 MHz, CD_3_CN) δ 212.6, 172.2, 144.4, 140.2, 128.5, 127.9, 126.1, 115.9, 115.4, 44.2, 39.8, 34.7, 27.7, 24.8, 24.8. **IR** (KBr): ν_max_ (cm^−1^) = 3648, 3523, 3442, 1715, 1627, 1400, 1275, 1260, 764, 749. **HRMS** (ESI^+^) calcd for C_17_H_20_NaO_2_ [M+Na]^+^: 279.1356, Found: 279.1362. 

**(*S*)-3-Hydroxy-2-(1-(4-isopropylphenyl)allyl)-6,6-dimethyl-cyclohex-2-en-1-one (3c),** pale yellow solid; **m.p.**: 99–101 °C; 78% yield (23.2 mg); **HPLC** *ee*: 90% [Daicel CHIRALPAK AD-H (0.46 cm × 25 cm); *n*-hexane/2-propanol = 90/10; flow rate = 1.0 mL/min; detection wavelength = 254 nm; t_R_ = 6.56 (minor), 7.41 (major) min]. **[α]_D_^20^** = −5.5 (c 1.0, CHCl_3_). **^1^H NMR** (600 MHz, DMSO-*d*_6_) δ 10.45 (s, 1H), 7.10–7.00 (m, 4H), 6.44–6.38 (m, 1H), 5.02–4.93 (m, 2H), 4.76 (d, *J* = 9.0 Hz, 1H), 2.52–2.50 (m, 3H), 1.73 (t, *J* = 6.6 Hz, 2H), 1.17 (d, *J* = 7.2 Hz, 6H), 0.99 (d, *J* = 3.6 Hz, 6H). ^**13**^**C NMR** (150 MHz, DMSO-*d*_6_) δ 201.1, 170.6, 145.4, 141.5, 140.5, 127.3, 126.0, 115.0, 114.9, 43.5, 34.2, 33.4, 26.9, 25.3, 25.2, 24.5, 24.4. **IR** (KBr): ν_max_ (cm^−1^) = 3498, 3431, 3011, 1676, 1632, 1413, 1265, 1243, 743. **HRMS** (ESI^+^) calcd for C_20_H_26_NaO_2_ [M + Na]^+^: 321.1825, Found: 321.1823.

**(*S*)-3-Hydroxy-2-(1-(4-methoxyphenyl)allyl)-6,6-dimethyl-cyclohex-2-en-1-one (3e),** yellow solid; **m.p.**: 75–77 °C; 70% yield (20.1 mg); **HPLC** *ee*: 83% [Daicel CHIRALPAK AD-H (0.46 cm × 25 cm); *n*-hexane/2-propanol = 90/10; flow rate = 1.0 mL/min; detection wavelength = 254 nm; t_R_ = 10.52 (minor), 12.24 (major) min]. **[α]_D_^20^** = +6.8 (c 1.0, CHCl_3_). **^1^H NMR** (600 MHz, CD_3_CN) δ 7.01 (d, *J* = 9.0 Hz, 2H), 6.71 (d, *J* = 9.0 Hz, 2H), 6.36–6.30 (m, 1H), 5.00–4.89 (m, 2H), 4.71 (d, *J* = 7.8 Hz, 1H), 3.65 (s, 3H), 2.45–2.42 (m, 2H), 1.70 (t, *J* = 6.0 Hz, 2H), 0.95 (d, *J* = 4.2 Hz, 6H). **^13^C NMR** (150 MHz, CD_3_CN) δ 202.2, 176.0, 158.2, 140.6, 135.9, 128.8, 116.0, 115.0, 113.7, 55.3, 43.3, 39.7, 34.5, 27.6, 24.7, 24.7. **IR** (KBr): ν_max_ (cm^−1^) = 3523, 3129, 3006, 2990, 1607, 1509, 1400, 1275, 1260, 764, 749. **HRMS** (ESI^+^) calcd for C_18_H_22_NaO_3_ [M + Na] ^+^: 309.1461, Found: 309.1462.

**(*S*)-2-(1-(3-Ethoxyphenyl)allyl)-3-hydroxy-6,6-dimethyl-cyclohex-2-en-1-one (3h),** yellow solid; **m.p.**: 83–85 °C; 76% yield (22.8 mg); **HPLC** *ee*: 89% [Daicel CHIRALPAK AD-H (0.46 cm × 25 cm); *n*-hexane/2-propanol = 90/10; flow rate = 1.0 mL/min; detection wavelength = 254 nm; t_R_ = 8.08 (major), 8.64 (minor) min]. **[α]_D_^20^** = −21.5 (c 1.0, CHCl_3_). **^1^H NMR** (600 MHz, DMSO-*d*_6_) δ 10.45 (s, 1H), 7.12–7.07 (m, 1H), 6.68 (d, *J* = 7.8 Hz, 1H), 6.65–6.63 (m, 2H), 6.42–6.36 (m, 1H), 5.00–4.97 (m, 2H), 4.75 (d, *J* = 9.0 Hz, 1H), 3.93 (dd, *J* = 6.6, 2.4 Hz, 2H), 2.50–2.49 (m, 2H), 1.72 (t, *J* = 6.6 Hz, 2H), 1.29 (t, *J* = 6.6 Hz, 3H), 0.99 (d, *J* = 3.6 Hz, 6H). ^**13**^**C NMR** (150 MHz, DMSO-*d*_6_) δ 201.0, 172.3, 159.7, 146.3, 140.5, 129.7, 120.4, 116.1, 115.6, 114.4, 112.1, 63.9, 44.4, 40.0, 34.8, 27.8, 25.1, 15.1. **IR** (KBr): ν_max_ (cm^−1^) = 3498, 3465, 3009, 1665, 1620, 1342, 1298, 1213, 734. **HRMS** (ESI^+^) calcd for C_19_H_24_NaO_3_ [M + Na]^+^: 323.1618, Found: 323.1617.

## 4. Conclusions

In conclusion, we developed Ir-catalyzed allylic enolization of 6,6-dimethyl-3-((trimethylsilyl)oxy)cyclohex-2-en-1-one involving keto-enol isomerization, which afforded the enolated allyl products in good to high yields with high regio- and enantioselectivities. This method allows the use of 6,6-dimethyl-3-((trimethylsilyl)oxy)cyclohex-2-en-1-one, tolerates numerous functionalized groups, and provides a new way for the construction of a chiral carbon-fluorine center.

## Figures and Tables

**Table 1:** Screening reaction conditions for Ir-catalyzed reaction between 6,6-dimethyl-3-((trimethylsilyl)oxy)cyclohex-2-en-1-one **1a** and allylic substrate **2a** ^a^.

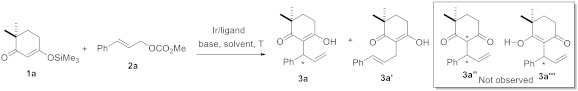								
Entry	Ir catalyst	Ligand	Base	Solvent	Temp (°C)	**3a**/**3a′**^b^	Yield ^b^ (%)	ee ^c^ (%)
1	[Ir(COD)Cl]_2_	L1	CsF	DCM	−20	>20/1	13	85
2	[Ir(COD)Cl]_2_	L1	CsF	THF	−20	-	-	-
3	[Ir(COD)Cl]_2_	L1	CsF	CH_3_CN	−20	>20/1	18	73
4	[Ir(COD)Cl]_2_	L1	CsF	Toluene	−20	>20/1	21	91
5	[Ir(COD)Cl]_2_	L1	CsCl	Toluene	−20	>20/1	19	70
6	[Ir(COD)Cl]_2_	L1	Cs_2_CO_3_	Toluene	−20	>20/1	27	91
7	[Ir(COD)Cl]_2_	L1	CsOH	Toluene	−20	>20/1	49	71
8	[Ir(COD)Cl]_2_	L1	K_2_CO_3_	Toluene	−20	>20/1	31	80
9	[Ir(COD)Cl]_2_	L1	DBU	Toluene	−20	-	nr	-
10	[Ir(COD)Cl]_2_	L1	Cs_2_CO_3_	Toluene	0	>20/1	52	87
11	[Ir(COD)Cl]_2_	L1	Cs_2_CO_3_	Toluene	10	>20/1	60	92
12	[Ir(COD)Cl]_2_	L1	Cs_2_CO_3_	Toluene	25	>20/1	75	91
13	[Ir(COD)Cl]_2_	L1	Cs_2_CO_3_	Toluene	35	>20/1	86	85
14	[Ir(Cp*)Cl_2_]_2_	L1	Cs_2_CO_3_	Toluene	25	-	nr	-
15	Ir(COD)(acac)	L1	Cs_2_CO_3_	Toluene	25	>20/1	19	93
16	[Ir(COD)Cl]_2_	L2	Cs_2_CO_3_	Toluene	25	>20/1	78	87
17	[Ir(COD)Cl]_2_	L3	Cs_2_CO_3_	Toluene	25	>20/1	30	78
18	[Ir(COD)Cl]_2_	L4	Cs_2_CO_3_	Toluene	25	-	-	-
19	[Ir(COD)Cl]_2_	L5	Cs_2_CO_3_	Toluene	25	-	-	-
20 ^d^	[Ir(COD)Cl]_2_	L1	Cs_2_CO_3_	Toluene	25	-	nr	-

^a^ Reaction conditions: Ir salt (0.004 mmol), ligand (0.008 mmol), **1a** (0.10 mmol), **2a** (0.20 mmol), base (0.12 mmol), and solvent (1 mL). ^b^ Isolated yield. ^c^ Determined by a chiral HPLC. ^d^ Either **1b** or **1c** instead of **1a** was used.

## Data Availability

Not applicable.

## Supplementary Materials

The following supporting information can be downloaded at: https://www.mdpi.com/article/10.3390/molecules27206981/s1, experimental details, NMR spectra, X-ray Crystallographic Information, HPLC Spectra, HRMS data for new products.
