# Supplementary material for: Ir-Catalyzed Chemo-, Regio-, and Enantioselective Allylic Enolization of 6,6-Dimethyl-3-((trimethylsilyl)oxy)cyclohex-2-en-1-one Involving Keto-Enol Isomerization
Source: Molecules. 2022 Oct 17;27(20):6981. doi: 10.3390/molecules27206981 (PMC9612087; doi:10.3390/molecules27206981)
Supplement: Supplementary file 1 [file molecules-27-06981-s001.zip › molecules-1934655-supplementary.pdf]

Supporting Information

Ir-Catalyzed chemo-, regio- and enantioselective allylic enolization of 6,6-dimethyl-3-((trimethylsilyl)oxy)cyclohex-2-en-1-one involving keto-enol isomerization

Xiao-Lin Wang, Ji-Teng Chen, Sheng-Cai Zheng* and Xiao-Ming Zhao*

School of Chemical Technology and Engineering, Tongji University, 1239 Siping Road, Shanghai, P.R. China, 200092

Table of Contents

1. General..S2

2. General Procedure for the Synthesis of **3**. S3

3. Procedure for the Synthesis of **5** S9

4. References..S10

5. X-ray Crystallographic Information of **3j** S11

6. Copies of NMR spectra of compounds **3** and **5** S13

7. HPLC Spectra of compounds **3** and **5**...S31

1. General

All manipulations were carried out under the argon atmosphere using standard Schlenk techniques. All glassware was oven or flame dried immediately prior to use. All solvents were purified and dried according to standard methods prior to use, unless stated otherwise.

^{1}H NMR spectra were obtained at 400 MHz or 600 MHz and recorded relative to the tetramethylsilane signal (0 ppm) or residual protio-solvent (7.26 ppm for $CDCl_3$, 1.94 ppm for CD_3CN, 2.50 ppm for DMSO-d_6). ^{13}C NMR spectra were obtained at 100 MHz or 150 MHz, and chemical shifts were recorded relative to the solvent resonance ($CDCl_3$, 77.16 ppm, CD_3CN, 1.32 ppm, 39.52 ppm for DMSO-d_6). ^{19}F NMR spectra were obtained at 376 MHz or 565 MHz. Data for NMR are recorded as follows: chemical shift (δ, ppm), multiplicity (s = singlet, d = doublet, t = triplet, m = multiplet or unresolved, br = broad singlet, coupling constant(s) in Hz, integration).

The phosphoramidite ligands[1], substituted allylic carbonates[2], were prepared according to the known procedures. Other chemicals were purchased from commercial suppliers and used without further purification, unless mentioned.

2. General Procedure for the Synthesis of 3

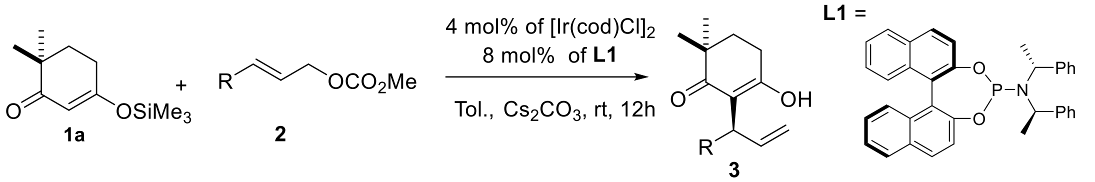

[Ir(COD)Cl]$_2$ (0.004 mmol, 4 mol%), phosphoramidite ligand **L1** (0.008 mmol, 8 mol%) were dissolved in THF (0.5 mL) and *n*-propylamine (0.3 mL) in a dry Schlenk tube filled with argon. The reaction mixture was heated at 50 °C for 30 min and then the volatile solvents were removed under vacuum to give a yellow solid. After that, allylic carbonate **2** (0.20 mmol), cesium carbonate (Cs_2CO_3, 0.12 mmol), and toluene (1.0 mL) were added. In another dry Schlenk tube, 6, 6-dimethyl-3-((trimethylsilyl) oxy) cyclohex-2-en-1-one **1a**[3] was prepared from 4, 4-dimethylcyclohexane-1, 3-cyclohexanedione (0.10 mmol) and hexamethyldisilazane (HMDS) (0.15 mmol) in DCM (2.0 mL) stirring for 2.5 h at room temperature, and the solvent was removed under vacuum to give a light-yellow liquid which was transferred through syringe into the above mentioned Schlenk tube. The reaction was stirring at room temperature for 12 h. Then the mixture was washed with brine. After the organic phase was collected, the aqueous phase was extracted with DCM. The combined organic phase was dried over anhydrous Na_2SO_4 and concentrated on a rotary evaporator. The crude residue was purified by flash column chromatography (petroleum ether/ethyl acetate) to give the desired products **3**.

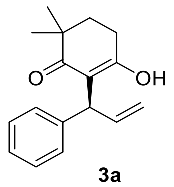

3a

(*S*)-3-Hydroxy-6,6-dimethyl-2-(1-phenylallyl)cyclohex-2-en-1-one (3a), white solid; **m.p.:** 103-105 °C; 75% yield (19.2 mg); **HPLC** *ee*: 91% [Daicel CHIRALPAK AD-H (0.46 cm × 25 cm); *n*-hexane/2-propanol = 90/10; flow rate = 1.0 mL/min; detection wavelength = 254 nm; t_R = 6.77 (minor), 8.22 (major) min]. **$[\alpha]_D^{20}$** = +22.3 (c 1.0, $CHCl_3$). **^{1}H NMR** (600 MHz, CD_3CN) δ 7.28 – 7.24 (m, 2H), 7.21 (d, *J* = 7.8 Hz, 2H), 7.17 – 7.13 (m, 1H), 6.49 – 6.43 (m, 1H), 5.11 – 5.05 (m, 2H), 4.88 (d, *J* = 7.8 Hz, 1H), 2.57 – 2.54 (m, 2H), 1.82 (t, *J* = 6.0 Hz, 2H), 1.06 (d, *J* = 6.0 Hz, 6H).**^{13}C NMR** (150 MHz, CD_3CN) δ 212.6, 172.2, 144.4, 140.2, 128.5, 127.9, 126.1, 115.9, 115.4, 44.2, 39.8, 34.7, 27.7, 24.8, 24.8. **IR** (KBr): ν_{max} (cm^{-1}) = 3648, 3523, 3442, 1715, 1627, 1400, 1275, 1260, 764, 749. **HRMS** (ESI$^+$) calcd for $C_{17}H_{20}NaO_2$ [M+Na]$^+$: 279.1356, Found: 279.1362.

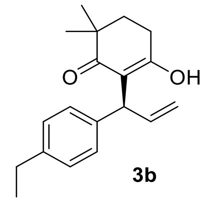

3b

(*S*)-2-(1-(4-Ethylphenyl)allyl)-3-hydroxy-6,6-dimethylcyclohex-2-en-1-one (3b), pale yellow wax; 71% yield (20.2 mg); **HPLC** *ee*: 90% [Daicel CHIRALCEL OJ-H (0.46 cm × 25 cm); *n*-hexane/2-propanol = 90/10; flow rate = 1.0 mL/min; detection wavelength = 254 nm; t_R = 8.45 (major), 12.06 (minor) min]. $[\alpha]_D^{20}$ = +12.7 (c 1.0, $CHCl_3$). **^{1}H NMR** (600 MHz, CD_3CN) δ 7.11 (d, *J* = 2.4 Hz, 4H), 6.49 – 6.42 (m, 1H), 5.10 – 5.04 (m, 2H), 4.85 (d, *J* = 8.4 Hz, 1H), 2.63 – 2.59 (m, 2H), 2.58 – 2.53 (m, 2H), 1.82 (t, *J* = 6.6 Hz, 2H), 1.21 (t, *J* = 7.2 Hz, 3H), 1.06 (d, *J* = 3.2 Hz, 6H). **^{13}C NMR** (150 MHz, CD_3CN) δ 202.3, 170.1, 141.9, 141.3, 140.4, 127.9, 127.8, 115.9, 115.1, 43.7, 39.7, 34.5, 28.5, 27.1, 24.7, 24.7, 15.8. **IR** (KBr): ν_{max} (cm^{-1}) = 3443, 3418, 3012, 1655, 1621, 1412, 1234, 1231, 745. **HRMS** (ESI^+) calcd for $C_{19}H_{24}NaO_2$ $[M+Na]^+$: 307.1669, Found: 307.1680.

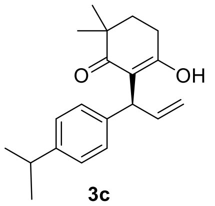

3c

(*S*)-3-Hydroxy-2-(1-(4-isopropylphenyl)allyl)-6,6-dimethylcyclohex-2-en-1-one (3c), pale yellow solid; **m.p.:** 99-101°C; 78% yield (23.2 mg); **HPLC** *ee*: 90% [Daicel CHIRALPAK AD-H (0.46 cm × 25 cm); *n*-hexane/2-propanol = 90/10; flow rate = 1.0 mL/min; detection wavelength = 254 nm; t_R = 6.56 (minor), 7.41 (major) min]. $[\alpha]_D^{20}$ = -5.5 (c 1.0, $CHCl_3$). **^{1}H NMR** (600 MHz, DMSO-d_6) δ 10.45 (s, 1H), 7.10 – 7.00 (m, 4H), 6.44 – 6.38 (m, 1H), 5.02 – 4.93 (m, 2H), 4.76 (d, *J* = 9.0 Hz, 1H), 2.52 – 2.50 (m, 3H), 1.73 (t, *J* = 6.6 Hz, 2H), 1.17 (d, *J* = 7.2 Hz, 6H), 0.99 (d, *J* = 3.6 Hz, 6H). **^{13}C NMR** (150 MHz, DMSO-d_6) δ 201.1, 170.6, 145.4, 141.5, 140.5, 127.3, 126.0, 115.0, 114.9, 43.5, 34.2, 33.4, 26.9, 25.3, 25.2, 24.5, 24.4. **IR** (KBr): ν_{max} (cm^{-1}) = 3498, 3431, 3011, 1676, 1632, 1413, 1265, 1243, 743. **HRMS** (ESI^+) calcd for $C_{20}H_{26}NaO_2$ $[M+Na]^+$: 321.1825, Found: 321.1823.

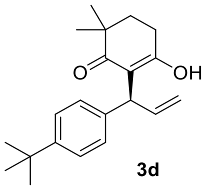

3d

(*S*)-2-(1-(4-(Tert-butyl)phenyl)allyl)-3-hydroxy-6,6-dimethylcyclohex-2-en-1-one (3d), pale yellow solid; **m.p.:** 91-94°C; 90% yield (28.1 mg); **HPLC** *ee*: 94% [Daicel CHIRALCEL OJ-H (0.46 cm × 25 cm); *n*-hexane/2-propanol = 95/5; flow rate = 0.5 mL/min; detection wavelength = 254 nm; t_R = 16.86 (minor), 19.18 (major) min]. $[\alpha]_D^{20}$ = +15.8 (c 1.0, $CHCl_3$). **^{1}H NMR** (600 MHz, DMSO-d_6) δ 10.45 (s, 1H), 7.22 (d, *J* = 8.4 Hz, 2H), 7.03 (d, *J* = 7.8 Hz, 2H), 6.43 – 6.37 (m, 1H),

5.02 – 4.94 (m, 2H), 4.75 (d, *J* = 8.4 Hz, 1H), 2.50 – 2.49 (m, 2H), 1.72 (t, *J* = 6.0 Hz, 2H), 1.24 (s, 9H), 0.99 (s, 6H). **^{13}C NMR** (150 MHz, DMSO-d_6) δ 201.1, 170.6, 147.6, 141.1, 140.5, 127.1, 124.9, 114.9, 114.8, 43.4, 34.4, 34.2, 31.7, 26.9, 25.3, 25.2. **IR** (KBr): ν_{max} (cm^{-1}) = 3586, 3523, 3441, 3129, 1650, 1400, 1275, 1269, 764, 752. **HRMS** (ESI$^+$) calcd for $C_{21}H_{27}NaO_2$ [M+Na]$^+$: 353.1887, Found: 353.1908.

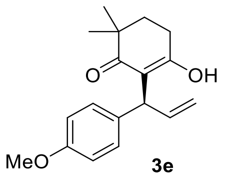
3e

(*S*)-3-Hydroxy-2-(1-(4-methoxyphenyl)allyl)-6,6-dimethylcyclohex-2-en-1-one (3e), yellow solid; **m.p.:** 75-77°C; 70% yield (20.1 mg); **HPLC** *ee*: 83% [Daicel CHIRALPAK AD-H (0.46 cm × 25 cm); *n*-hexane/2-propanol = 90/10; flow rate = 1.0 mL/min; detection wavelength = 254 nm; t_R = 10.52 (minor), 12.24 (major) min]. **$[\alpha]_D^{20}$** = +6.8 (c 1.0, $CHCl_3$). **^{1}H NMR** (600 MHz, CD_3CN) δ 7.01 (d, *J* = 9.0 Hz, 2H), 6.71 (d, *J* = 9.0 Hz, 2H), 6.36 -6.30 (m, 1H), 5.00 – 4.89 (m, 2H), 4.71 (d, *J* = 7.8 Hz, 1H), 3.65 (s, 3H), 2.45 – 2.42 (m, 2H), 1.70 (t, *J* = 6.0 Hz, 2H), 0.95 (d, *J* = 4.2 Hz, 6H). **^{13}C NMR** (150 MHz, CD_3CN) δ 202.2, 176.0, 158.2, 140.6, 135.9, 128.8, 116.0, 115.0, 113.7, 55.3, 43.3, 39.7, 34.5, 27.6, 24.7, 24.7. **IR** (KBr): ν_{max} (cm^{-1}) = 3523, 3129, 3006, 2990, 1607, 1509, 1400, 1275, 1260, 764, 749. **HRMS** (ESI$^+$) calcd for $C_{18}H_{22}NaO_3$ [M+Na] $^+$: 309.1461, Found: 309.1462.

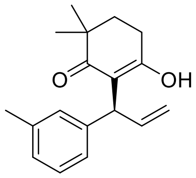
3f

(*S*)-3-Hydroxy-6,6-dimethyl-2-(1-(m-tolyl)allyl)cyclohex-2-en-1-one (3f), yellow solid; **m.p.:** 87-89°C; 70% yield (21.1 mg); **HPLC** *ee*: 82% [Daicel CHIRALPAK IC (0.46 cm × 25 cm); *n*-hexane/2-propanol = 95/5; flow rate = 0.5 mL/min; detection wavelength = 254 nm; t_R = 22.16 (minor), 23.36 (major) min]. **$[\alpha]_D^{20}$** = +7.9 (c 1.0, $CHCl_3$). **^{1}H NMR** (600 MHz, DMSO-*d6)* δ 10.46 (s, 1H), 7.08 (t, *J* = 7.8 Hz, 1H), 6.93 (s, 1H), 6.90 (d, *J* = 7.8 Hz, 2H), 6.44 – 6.37 (m, 1H), 5.03 – 4.96 (m, 2H), 4.75 (d, *J* = 8.4 Hz, 1H), 2.51 – 2.50 (m, 2H), 2.23 (s, 3H), 1.73 (t, *J* = 6.6 Hz, 2H), 0.99 (d, *J* = 3.0 Hz, 6H). **^{13}C NMR** (150 MHz, DMSO) δ 201.0, 170.7, 144.2, 140.4, 136.9, 128.1, 128.0, 126.2, 124.5, 115.1, 114.9, 43.7, 34.2, 26.9, 25.2, 25.1, 21.6. **IR** (KBr): ν_{max} (cm^{-1}) = 3509, 3440, 3127, 1628, 1607, 1440, 1275, 1234, 765. **HRMS** (ESI$^+$) calcd for $C_{18}H_{22}NaO_2$ [M+Na]$^+$: 293.1512, Found: 293.1513.

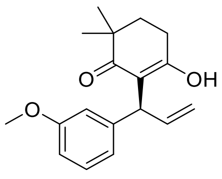

3g

(*S*)-3-Hydroxy-2-(1-(3-methoxyphenyl)allyl)-6,6-dimethylcyclohex-2-en-1-one (3g), yellow solid; **m.p.:** 83-85°C; 60% yield (20.0 mg); **HPLC** *ee*: 92% [Daicel CHIRALCEL OJ-H (0.46 cm × 25 cm); *n*-hexane/2-propanol = 95/5; flow rate = 1.0 mL/min; detection wavelength = 254 nm; t_R = 23.39 (major), 26.21 (minor) min]. **$[\alpha]_D^{20}$** = +16.9 (c 1.0, $CHCl_3$). **^{1}H NMR** (600 MHz, CD_3CN) δ 7.87 (s, 1H), 7.17 (t, *J* = 7.8 Hz, 1H), 6.80 (d, *J* = 9.0 Hz, 1H), 6.77 – 6.70 (m, 2H), 6.47 – 6.41 (m, 1H), 5.09 – 5.05 (m, 2H), 4.85 (d, *J* = 8.4 Hz, 1H), 3.75 (s, 3H), 2.57 – 2.54 (m, 2H), 1.82 (t, *J* = 6.0 Hz, 2H), 1.07 (d, *J* = 3.0 Hz, 6H). **^{13}C NMR** (150 MHz, CD_3CN) δ 202.2, 170.1, 160.1, 145.9, 140.1, 129.4, 120.1, 115.8, 115.4, 113.5, 111.2, 55.2, 44.0, 40.0, 34.4, 27.0, 24.7. **IR** (KBr): ν_{max} (cm^{-1}) = 3651, 3511, 3112, 1677, 1609, 1412, 1212, 1205, 722. **HRMS** (ESI^+) calcd for $C_{18}H_{22}NaO_3$ $[M+Na]^+$: 309.1461, Found: 309.1469.

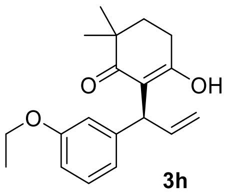

3h

(*S*)-2-(1-(3-Ethoxyphenyl)allyl)-3-hydroxy-6,6-dimethylcyclohex-2-en-1-one (3h), yellow solid; **m.p.:** 83-85°C; 76% yield (22.8 mg); **HPLC** *ee*: 89% [Daicel CHIRALPAK AD-H (0.46 cm × 25 cm); *n*-hexane/2-propanol = 90/10; flow rate = 1.0 mL/min; detection wavelength = 254 nm; t_R = 8.08 (major), 8.64 (minor) min]. **$[\alpha]_D^{20}$** = -21.5 (c 1.0, $CHCl_3$). **^{1}H NMR** (600 MHz, DMSO-*d*$_6$) δ 10.45 (s, 1H), 7.12 – 7.07 (m, 1H), 6.68 (d, *J* = 7.8 Hz, 1H), 6.65 – 6.63 (m, 2H), 6.42 – 6.36 (m, 1H), 5.00 – 4.97 (m, 2H), 4.75 (d, *J* = 9.0 Hz, 1H), 3.93 (dd, *J* = 6.6, 2.4 Hz, 2H), 2.50 – 2.49 (m, 2H), 1.72 (t, *J* = 6.6 Hz, 2H), 1.29 (t, *J* = 6.6 Hz, 3H), 0.99 (d, *J* = 3.6 Hz, 6H). **^{13}C NMR** (150 MHz, DMSO-*d*$_6$) δ 201.0, 172.3, 159.7, 146.3, 140.5, 129.7, 120.4, 116.1, 115.6, 114.4, 112.1, 63.9, 44.4, 40.0, 34.8, 27.8, 25.1, 15.1. **IR** (KBr): ν_{max} (cm^{-1}) = 3498, 3465, 3009, 1665, 1620, 1342, 1298, 1213, 734. **HRMS** (ESI^+) calcd for $C_{19}H_{24}NaO_3$ $[M+Na]^+$: 323.1618, Found: 323.1617.

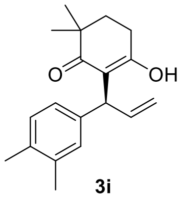

3i

(*S*)-2-(1-(3,4-Dimethylphenyl)allyl)-3-hydroxy-6,6-dimethylcyclohex-2-en-1-one (3i), yellow solid; **m.p.:** 108-110°C; 70% yield (19.9 mg); **HPLC** *ee*: 91% [Daicel CHIRALPAK AD-H (0.46 cm × 25 cm); *n*-hexane/2-propanol = 90/10; flow rate = 1.0 mL/min; detection wavelength = 254 nm; t_R = 6.07 (minor), 6.71 (major) min]. **$[\alpha]_D^{20}$** = -30.2 (c 1.0, $CHCl_3$). **^{1}H NMR** (600 MHz, CD_3CN) δ 7.75 (s, 1H), 7.01 (d, *J* = 7.8 Hz, 1H), 6.96 (s, 1H), 6.92 (d, *J* = 7.8 Hz, 1H), 6.47 – 6.41

(m, 1H), 5.09 – 5.02 (m, 2H), 4.82 (d, *J* = 8.4 Hz, 1H), 2.55 – 2.52 (m, 2H), 2.21 (s, 6H), 1.81 (t, *J* = 6.0 Hz, 2H), 1.06 (s, 6H). **^{13}C NMR** (150 MHz, CD_3CN) δ 200.6, 172.4, 141.5, 140.5, 136.4, 129.6, 129.0, 125.2, 116.0, 115.0, 43.6, 39.7, 34.5, 27.5, 24.8, 24.7, 19.5, 18.9. **IR** (KBr): ν_{max} (cm^{-1}) = 3521, 3422, 3123, 1710, 1676, 1423, 1211, 1201, 725. **HRMS** (ESI^+) calcd for $C_{19}H_{24}NaO_2$ $[M+Na]^+$: 307.1669, Found: 307.1661.

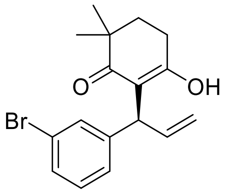

3j

(*S*)-2-(1-(3-Bromophenyl)allyl)-3-hydroxy-6,6-dimethylcyclohex-2-en-1-one (3j), white solid; **m.p.:** 90-92°C; 70% yield (23.4 mg); **HPLC** *ee*: 81% [Daicel CHIRALPAK AD-H (0.46 cm × 25 cm); *n*-hexane/2-propanol = 90/10; flow rate = 1.0 mL/min; detection wavelength = 254 nm; t_R = 6.42 (minor), 7.12 (major) min]. **$[\alpha]_D^{20}$** = +15.8 (c 1.0, $CHCl_3$). **^{1}H NMR** (600 MHz, DMSO-d_6) δ 10.73 (s, 1H), 7.30 (d, *J* = 7.8 Hz, 1H), 7.25 (s, 1H), 7.18 (t, *J* = 7.2 Hz, 1H), 7.10 (d, *J* = 7.8 Hz, 1H), 6.38 – 6.32 (m, 1H), 5.09 – 4.99 (m, 2H), 4.78 (d, *J* = 8.4 Hz, 1H), 2.53 – 2.50 (m, 2H), 1.72 (t, *J* = 6.6 Hz, 2H), 0.98 (d, *J* = 5.4 Hz, 6H). **^{13}C NMR** (150 MHz, DMSO-d_6) δ 201.0, 171.2, 147.3, 139.2, 130.4, 130.1, 128.5, 126.5, 121.7, 116.1, 114.2, 43.5, 34.1, 26.8, 25.2, 25.1. **IR** (KBr):ν_{max} (cm^{-1}) = 3508, 3441, 3116, 1670, 1628, 1400, 1273, 1261, 763, 748. **HRMS** (ESI^+) calcd for $C_{17}H_{19}BrNaO_2$ $[M+Na]^+$: 357.0461, Found: 357.0457.

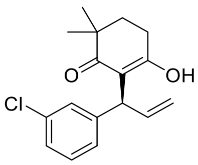

3k

(*S*)-2-(1-(3-Chlorophenyl)allyl)-3-hydroxy-6,6-dimethylcyclohex-2-en-1-one (3k), pale yellow wax; 69% yield (20.0 mg); **HPLC** *ee*: 87% [Daicel CHIRALPAK AD-H (0.46 cm × 25 cm); *n*-hexane/2-propanol = 90/10; flow rate = 1.0 mL/min; detection wavelength = 254 nm; t_R = 5.89 (minor), 6.56 (major) min]. **$[\alpha]_D^{20}$** = +8.9 (c 1.0, $CHCl_3$). **^{1}H NMR** (600 MHz, CD_3CN) δ 7.27 – 7.20 (m, 2H), 7.19 – 7.11 (m, 2H), 6.43 – 6.38 (m, 1H), 5.13 – 5.07 (m, 2H), 4.85 (d, *J* = 8.4 Hz, 1H), 2.59 – 2.55 (m, 2H), 1.82 (t, *J* = 6.6 Hz, 2H), 1.05 (d, *J* = 8.4 Hz, 6H). **^{13}C NMR** (150 MHz, CD_3CN) δ 201.9, 178.5, 147.1, 139.3, 133.7, 130.0, 127.7, 126.3, 125.9, 115.9, 115.4, 43.8, 39.7, 34.4, 27.3, 24.6, 24.5. **IR** (KBr): ν_{max} (cm^{-1}) = 3613, 3524, 34426, 3009, 1660, 1392, 1275, 1260, 765, 746. **HRMS** (ESI^+) calcd for $C_{17}H_{19}ClNaO_2$ $[M+Na]^+$: 313.0966, Found: 313.0996.

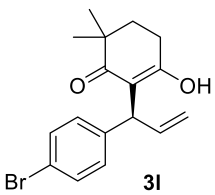

3l

(*S*)-2-(1-(4-Bromophenyl)allyl)-3-hydroxy-6,6-dimethylcyclohex-2-en-1-one (3l), yellow solid; **m.p.:** 93-95°C; 62% yield (20.7 mg); **HPLC** *ee*: 86% [Daicel CHIRALPAK AD-H (0.46 cm × 25 cm); *n*-hexane/2-propanol = 90/10; flow rate = 1.0 mL/min; detection wavelength = 254 nm; t_R = 7.09 (minor), 8.95 (major) min]. **$[\alpha]_D^{20}$** = +39.9 (c 1.0, $CHCl_3$). **^{1}H NMR** (600 MHz, DMSO-d_6) δ 10.61 (s, 1H), 7.39 (d, *J* = 8.4 Hz, 2H), 7.06 (d, *J* = 8.4 Hz, 2H), 6.39 – 6.32 (m, 1H), 5.03 – 4.99 (m, 2H), 4.75 (d, *J* = 8.4 Hz, 1H), 2.51 (t, *J* = 1.8 Hz, 2H), 1.72 (t, *J* = 6.0 Hz, 2H), 0.97 (d, *J* = 7.8 Hz, 6H). **^{13}C NMR** (150 MHz, DMSO-d_6) δ 201.0, 171.0, 143.9, 139.5, 131.0, 129.7, 118.6, 115.8, 114.5, 43.2, 34.2, 26.8, 25.2, 25.1. **IR** (KBr): ν_{max} (cm^{-1}) = 3648, 3526, 3442, 3207, 1704, 1628, 1416, 1386, 1270, 1010, 655. **HRMS** (ESI^+) calcd for $C_{17}H_{19}BrNaO_2$ $[M+Na]^+$: 357.0461, Found: 357.0448.

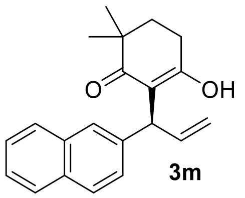

3m

(*S*)-3-Hydroxy-6,6-dimethyl-2-(1-(naphthalen-2-yl)allyl)cyclohex-2-en-1-one (3m), white solid; **m.p.:** 139-141°C; 90% yield (27.5 mg); **HPLC** *ee*: 90% [Daicel CHIRALPAK AD-H (0.46 cm × 25 cm); *n*-hexane/2-propanol = 90/10; flow rate = 1.0 mL/min; detection wavelength = 254 nm; t_R = 10.39 (major), 9.68 (minor) min]. **$[\alpha]_D^{20}$** = -46.6 (c 1.0, $CHCl_3$). **^{1}H NMR** (600 MHz, DMSO-d_6) δ 10.59 (s, 1H), 7.80 (t, *J* = 7.9 Hz, 2H), 7.74 (d, *J* = 8.5 Hz, 1H), 7.59 (s, 1H), 7.46 – 7.39 (m, 2H), 7.29 (dd, *J* = 8.5, 1.8 Hz, 1H), 6.55 – 6.48 (m, 1H), 5.08 (dq, *J* = 13.6, 2.5 Hz, 2H), 4.96 (d, *J* = 8.5 Hz, 1H), 2.51 (t, *J* = 1.9 Hz, 2H), 1.75 (t, *J* = 6.4 Hz, 2H), 0.99 (d, *J* = 7.6 Hz, 6H). **^{13}C NMR** (150 MHz, DMSO-d_6) δ 201.2, 170.9, 142.1, 140.0, 133.4, 127.9, 127.7, 127.5, 126.8, 126.2, 125.4, 125.1, 115.6, 114.8, 43.9, 34.2, 26.8, 25.3, 25.1. **IR** (KBr): ν_{max} (cm^{-1}) = 3523, 3440, 3127, 3007, 1641, 1625, 1605, 1400, 1276, 1268, 769, 752. **HRMS** (ESI^+) calcd for $C_{21}H_{22}NaO_2$ $[M+Na]^+$: 329.1512, Found: 329.1515.

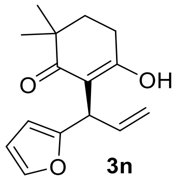

3n

(*R*)-2-(1-(Furan-2-yl)allyl)-3-hydroxy-6,6-dimethylcyclohex-2-en-1-one (3n), brown solid; **m.p.:** 100-102°C; 61% yield (15.0 mg); **HPLC** *ee*: 92% [Daicel CHIRALPAK IG (0.46 cm × 25 cm); *n*-hexane/2-propanol = 90/10; flow rate = 1.0 mL/min; detection wavelength = 220 nm; t_R = 7.28 (minor), 8.92 (major) min]. **$[\alpha]_D^{20}$** = -4.6 (c 1.0, $CHCl_3$). **^{1}H NMR** (600 MHz, CD_3CN) δ 7.23 (s, 1H), 6.26 – 6.21 (m, 1H), 6.20 – 6.19 (m, 1H), 5.88 (d, *J* = 3.2 Hz, 1H), 5.00 – 4.94 (m, 2H), 4.82 (d, *J* = 7.8 Hz, 1H), 2.43 (td, *J* = 6.6, 1.8 Hz, 2H), 1.70 (t, *J* = 6.6 Hz, 2H), 0.96 (s, 6H). **^{13}C NMR** (150 MHz, CD_3CN) δ 200.3, 173.5, 157.1, 141.2, 138.0, 115.3, 113.3, 110.8, 105.3, 39.6, 38.5, 34.5, 27.6, 24.7. **IR** (KBr): ν_{max} (cm^{-1}) = 3524, 3442, 3129, 1627, 1590, 1400, 1277, 1260, 764, 747. **HRMS** (ESI^+) calcd for $C_{15}H_{18}NaO_3$ $[M+Na]^+$: 269.1148, Found: 269.1168.

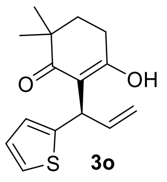

3o

(*R*)-3-Hydroxy-6,6-dimethyl-2-(1-(thiophen-2-yl)allyl)cyclohex-2-en-1-one 3o, yellow solid; **m.p.:** 99-101°C; 63% yield (16.5 mg); **HPLC** *ee*: 93% [Daicel CHIRALPAK IG (0.46 cm × 25 cm); *n*-hexane/2-propanol = 90/10; flow rate = 1.0 mL/min; detection wavelength = 220 nm; t_R = 7.62 (minor), 10.17 (major) min]. **$[\alpha]_D^{20}$** = +4.8 (c 1.0, $CHCl_3$). **^{1}H NMR** (600 MHz, CD_3CN) δ 7.16 (d, *J* = 5.4 Hz, 1H), 6.90 (dd, *J* = 4.8, 3.6 Hz, 1H), 6.75 (dd, *J* = 3.6, 1.8 Hz, 1H), 6.51 – 6.45 (m, 1H), 5.12 (dt, *J* = 15.6, 1.2 Hz, 1H), 5.08 – 5.03 (m, 2H), 2.56 (td, *J* = 6.6, 1.8 Hz, 2H), 1.82 (t, *J* = 6.6 Hz, 2H), 1.08 (d, *J* = 3.0 Hz, 6H). **^{13}C NMR** (150 MHz, CD_3CN) δ 148.7, 140.0, 127.0, 123.9, 123.7, 116.0, 115.2, 40.3, 39.7, 34.4, 27.2, 24.6. **IR** (KBr): ν_{max} (cm^{-1}) = 3510, 3442, 3128, 3002, 1647, 1400, 1275, 1261, 761, 742. **HRMS** (ESI^+) calcd for $C_{15}H_{18}NaO_2S$ $[M+Na]^+$: 285.0920, Found: 285.0913.

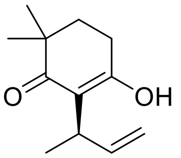

3p

(*R*)-2-(But-3-en-2-yl)-3-hydroxy-6,6-dimethylcyclohex-2-en-1-one (3p), white wax; 60% yield (11.6 mg); **HPLC** *ee*: 91% [Daicel CHIRALCEL OJ-H (0.46 cm × 25 cm); *n*-hexane/2-propanol = 90/10; flow rate = 1.0 mL/min; detection wavelength = 254 nm; t_R = 6.56 (major), 7.71 (minor) min]. **$[\alpha]_D^{20}$** = +24.6 (c 1.0, $CHCl_3$). **^{1}H NMR** (600 MHz, DMSO-d_6) δ 10.28 (s, 1H), 6.06 – 6.01 (m, 1H), 4.84 (dt, *J* = 17.4, 1.8 Hz, 1H), 4.76 (dt, *J* = 10.2, 1.8 Hz, 1H), 3.62 – 3.57 (m, 1H), 2.44 (t, *J* = 6.6 Hz, 2H), 1.67 (t, *J* = 6.6 Hz, 2H), 1.11 (d, *J* = 7.2 Hz, 3H), 0.97 (s, 6H). **^{13}C NMR** (150 MHz, DMSO-d_6) δ 201.4, 169.7, 143.5, 115.8, 112.1, 34.3, 32.9, 25.3, 25.3, 18.8. **IR** (KBr): ν_{max} (cm^{-1}) = 3586, 3523, 3442, 3209, 1665, 1608, 1409, 1311, 1215, 1112, 756. HRMS (ESI^+) calcd for $C_{12}H_{18}NaO_2$ $[M+Na]^+$: 217.1199, Found: 217.1195.

3. Procedure for the Synthesis of 5[4]

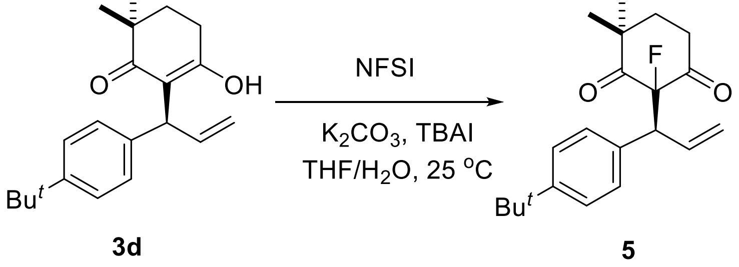

A round bottom flask equipped with a magnetic stir bar was charged with 2-(1-(4-(tert-butyl)phenyl)allyl)-3-hydroxy-6,6-dimethylcyclohex-2-en-1-one **3d** (31.2 mg, 0.1 mmol), and then a mixure of tetrahydrofuran and water (2.0 mL) in 7/3 proportion was added via a glass syringe and followed by the addition of TBAI (3.7 mg, 0.01 mmol) and K_2CO_3(34.5 mg, 0.25 mmol). The resulting reaction mixture was stirred for 30 min at room temperature and then NFSI (34.7 mg, 0.11 mmol) was added. The reaction was allowed to stir for another 2 h, then quenched with water (5.0 mL) and extracted with ethyl acetate (3 x 5.0 mL). The combined organic layers were washed with brine and dried over anhydrous Na_2SO_4, filtered and concentrated. The crude product was purified by flash column chromatography over silica gel using 5% ethyl acetate in petroleum ether as eluent to afford the pure product **5** in 86% yield.

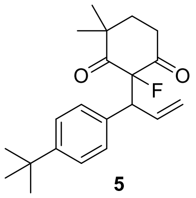

5

2-(1-(4-(Tert-butyl)phenyl)allyl)-2-fluoro-4,4-dimethylcyclohexane-1,3-dione (5), white solid; **m.p.:** 102-104°C; 86% yield (28.4 mg); **HPLC** *ee*: 99% [Daicel CHIRALPAK AD-H (0.46 cm × 25 cm); *n*-hexane/2-propanol = 90/10; flow rate = 1.0 mL/min; detection wavelength = 254 nm; t_R = 5.478 (major), 7.106 (minor) min]. $[\alpha]_D^{20}$ = +32.5 (c 1.0, $CHCl_3$). **^{1}H NMR** (400 MHz, Chloroform-*d*) δ 7.32 (d, *J* = 8.4 Hz, 2H), 7.15 (d, *J* = 8.0 Hz, 2H), 6.24 – 6.15 (m, 1H), 5.22 (d, *J* = 10.0 Hz, 1H), 5.08 (d, *J* = 17.2 Hz, 1H), 4.12 (dd, *J* = 28.0, 8.8 Hz, 1H), 2.65 (t, *J* = 8.0 Hz, 2H), 2.12 – 2.04 (m, 1H), 1.78 – 1.72 (m, 1H), 1.32 (s, 3H), 1.28 (s, 9H), 1.13 (s, 3H). **^{13}C NMR** (100 MHz, Chloroform-*d*) δ 205.3 (d, *J* = 14.8 Hz), 200.6 (d, *J* = 15.8 Hz), 151.1, 133.3 (d, *J* = 6.0 Hz), 132.6, 128.2 (d, *J* = 2.1 Hz), 125.9, 119.0, 103.7 (d, *J* = 208.3 Hz), 53.9 (d, *J* = 21.3 Hz), 44.4, 34.8, 34.5, 31.2, 30.8, 25.9, 25.4. **^{19}F NMR** (376 MHz, Chloroform-*d*) δ -174.1. **IR** (KBr): $_{vmax}$ (cm^{-1}) = 3652, 3542, 3212, 3098, 1675, 1623, 1521, 1476, 1298, 1279, 765, 749. **HRMS** (ESI^+) calcd for $C_{21}H_{27}FNaO_2$ $[M+Na]^+$: 353.1887, Found: 353.1908.

4. References

1. a) A. Alexakis, S. Rosset, J. Allamand, S. March, F. Guillen, C. Benhaim, *Synlett.* **2001**, *9*, 1375. b) R. Naasz, L. A. Arnold, A. J. Minnaard, B. L. Feringa, *Angew. Chem. Int. Ed.* **2001**, *40*, 927. c) D. Polet, A. Alexakis, *Synthesis.* **2004**, *15*, 2586.

3. P. G. M. Wuts, S. W. Ashford, A. M. Anderson, J. R. Atkins, *Org. Lett.* **2003**, *5*,1483.

4. D. T. W. Chu, S. N. Huckin, *Can. J. Chem.* **1980**, *58*, 138.

5. K. Jain, K. Das. *Synthetic Communications*. **2018**, *48*, 1.

5. X-ray Crystallographic Information of 3j

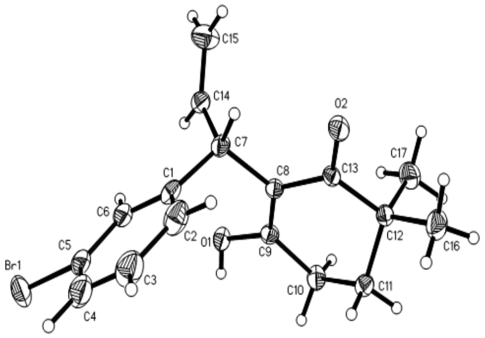

Table 1. Crystal data and structure refinement for compound 3j. (CCDC : 2098319)

CCDC 2098319 (**3j**) contains the supplementary crystallographic data for this paper. These data can be obtained free of charge from The Cambridge Crystallographic Data Centre via www.ccdc.cam.ac.uk/data_request/cif.

Empirical formula	$C_{17}H_{19}BrO_2$
Formula weight	335.23
Temperature [K]	288(2)
Crystal system	monoclinic
Space group (number)	$P2_1$ (4)
a [Å]	10.4035(3)
b [Å]	13.6608(4)
c [Å]	11.3206(4)
α [°]	90
β [°]	97.2470(10)
γ [°]	90
Volume [Å^3]	1596.03(9)
Z	4
ρ_{calc} [gcm^{-3}]	1.395
μ [mm^{-1}]	2.574
F(000)	688
Crystal size [mm^3]	0.220×0.210×0.080
Crystal colour	colourless
Crystal shape	block
Radiation	MoK_α (λ=0.71073 Å)
2Θ range [°]	5.69 to 50.04 (0.84 Å)
Index ranges	$-12 \leq h \leq 12$
	$-16 \leq k \leq 16$
	$-13 \leq l \leq 13$
Reflections collected	26284

Independent reflections	5605<br>R_{int} = 0.0286<br>R_{sigma} = 0.0452
Completeness to<br>ϴ = 25.019°	99.7 %
Data / Restraints / Parameters	5605/395/431
Goodness-of-fit on F^2	1.065
Final *R* indexes<br>[$I \geq 2\sigma(I)$]	R_1 = 0.0553<br>wR_2 = 0.1612
Final *R* indexes<br>[all data]	R_1 = 0.0674<br>wR_2 = 0.1715
Largest peak/hole [eÅ^{-3}]	1.18/-0.68
Flack X parameter	0.020(5)

6. Copies of NMR spectra of compounds 3 and 5

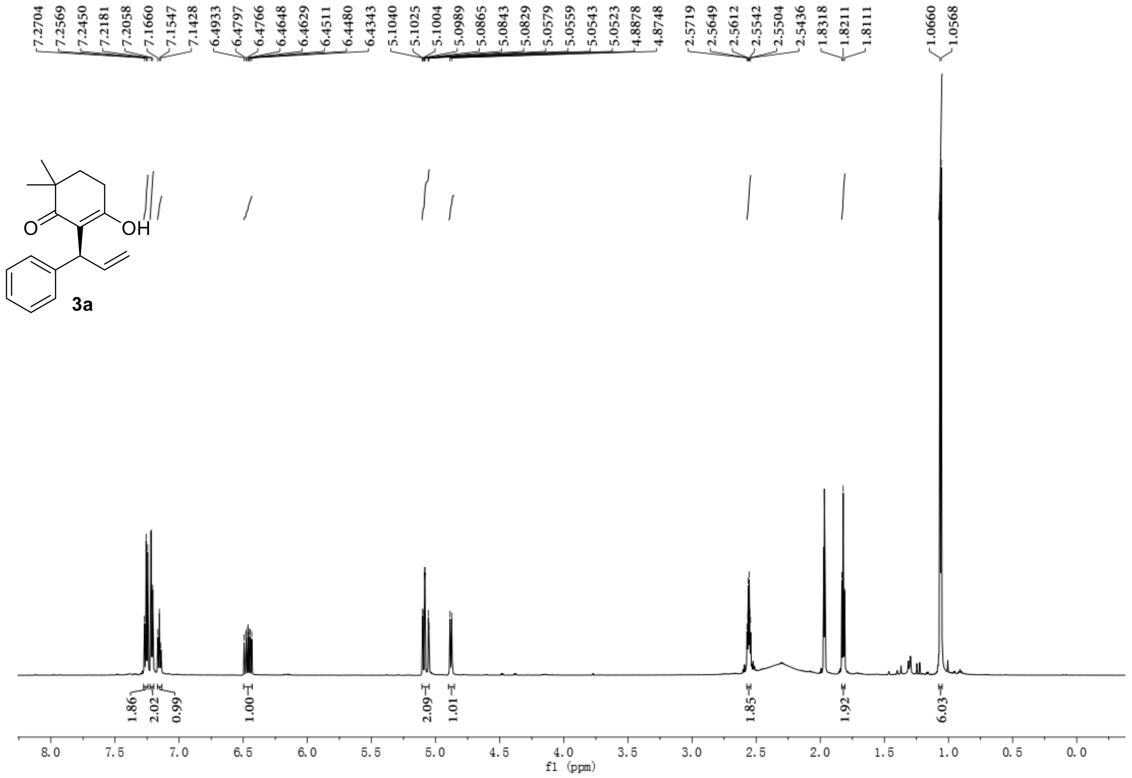

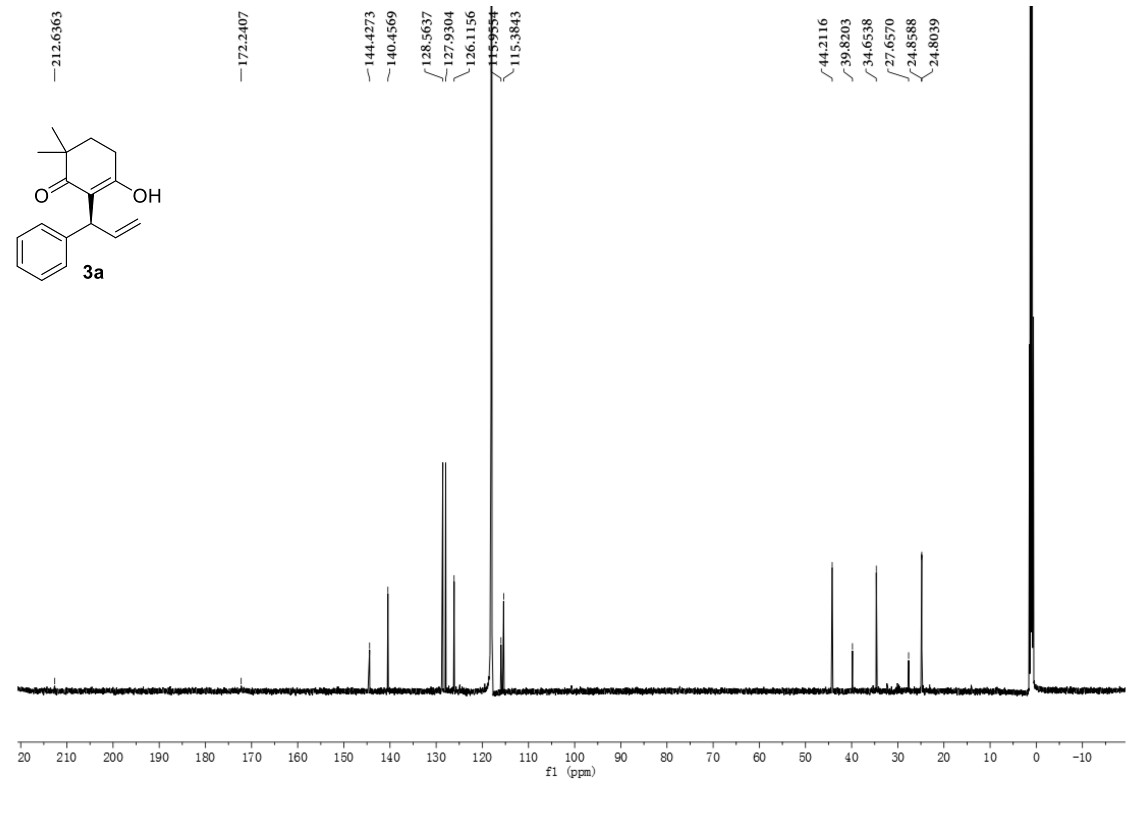

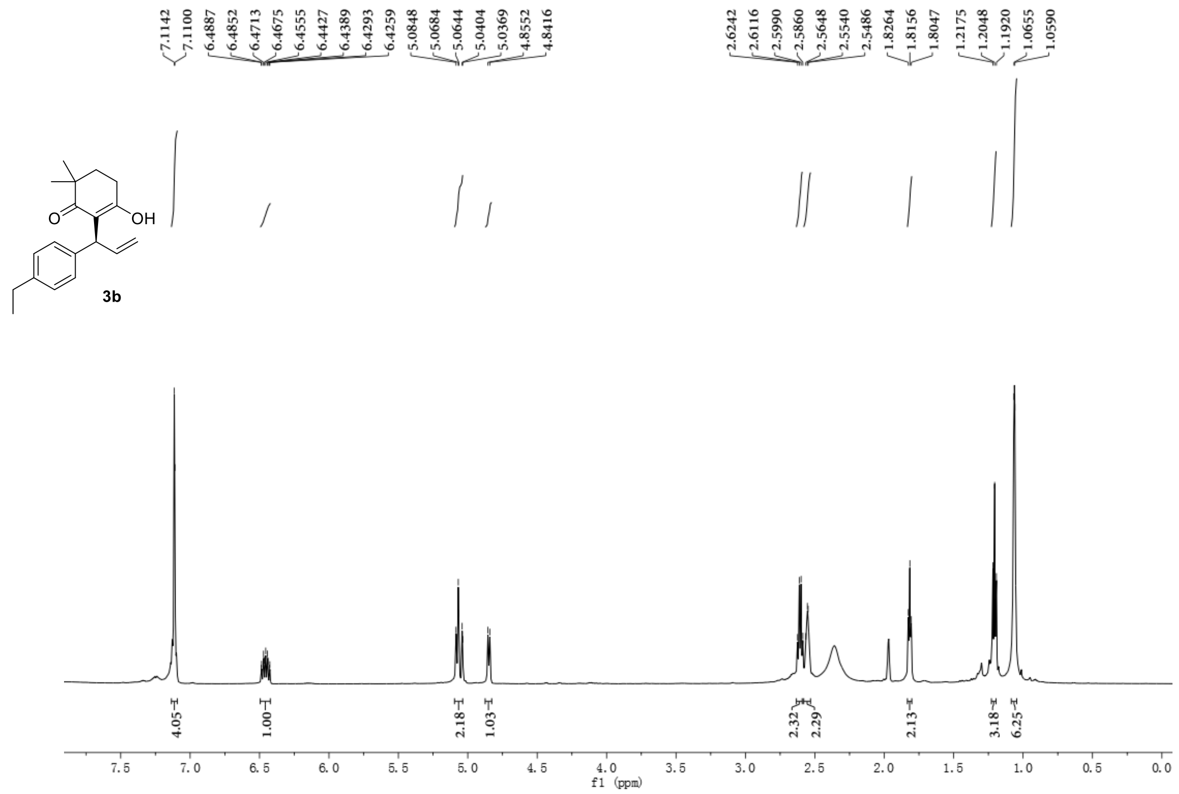
7.1142
7.1100
6.4887
6.4852
6.4713
6.4675
6.4555
6.4427
6.4389
6.4293
6.4259
5.0848
5.0684
5.0644
5.0404
5.0369
4.8552
4.8416
2.6242
2.6116
2.5990
2.5860
2.5648
2.5540
2.5486
1.8264
1.8156
1.8047
1.2175
1.2048
1.1920
1.0655
1.0590
O
OH
3b
4.05
1.00
2.18
1.03
2.32
2.29
2.13
3.18
6.25
f1 (ppm)

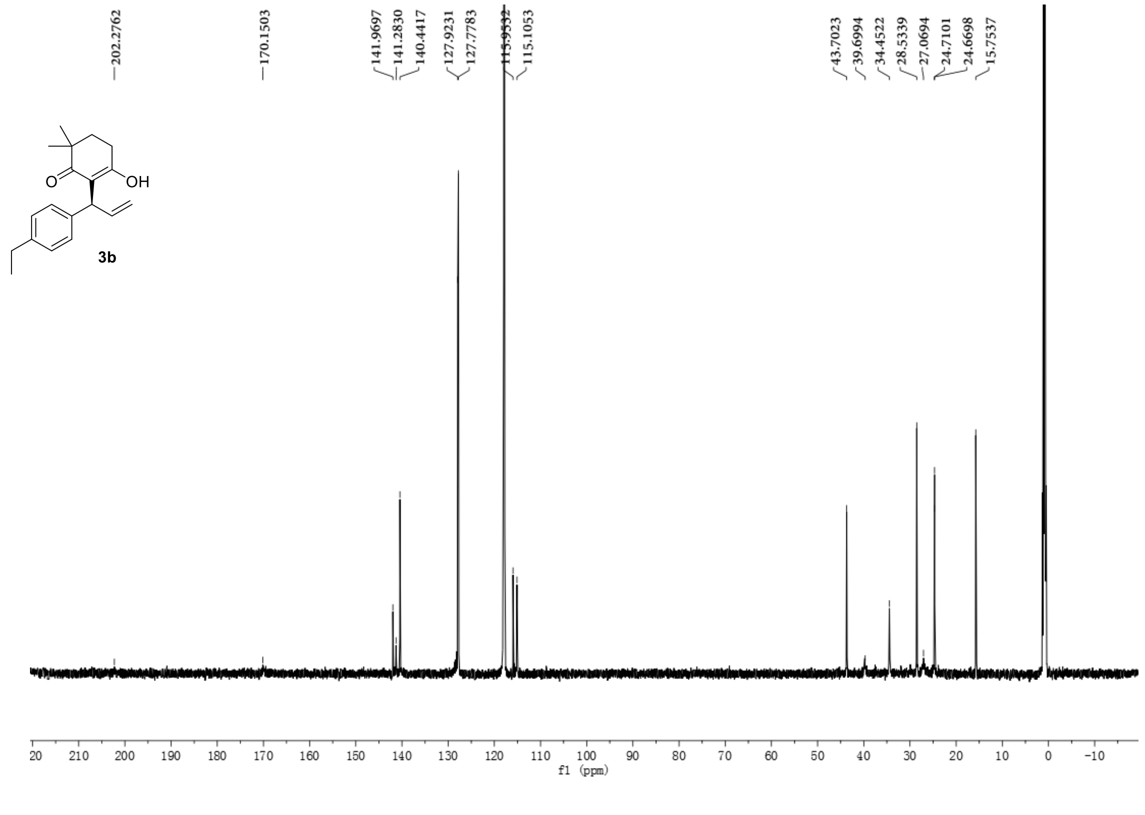
202.2762
170.1503
141.9697
141.2830
140.4417
127.9231
127.7783
115.9532
115.1053
43.7023
39.6994
34.4522
28.5339
27.0694
24.7101
24.6698
15.7537
O
OH
3b
f1 (ppm)

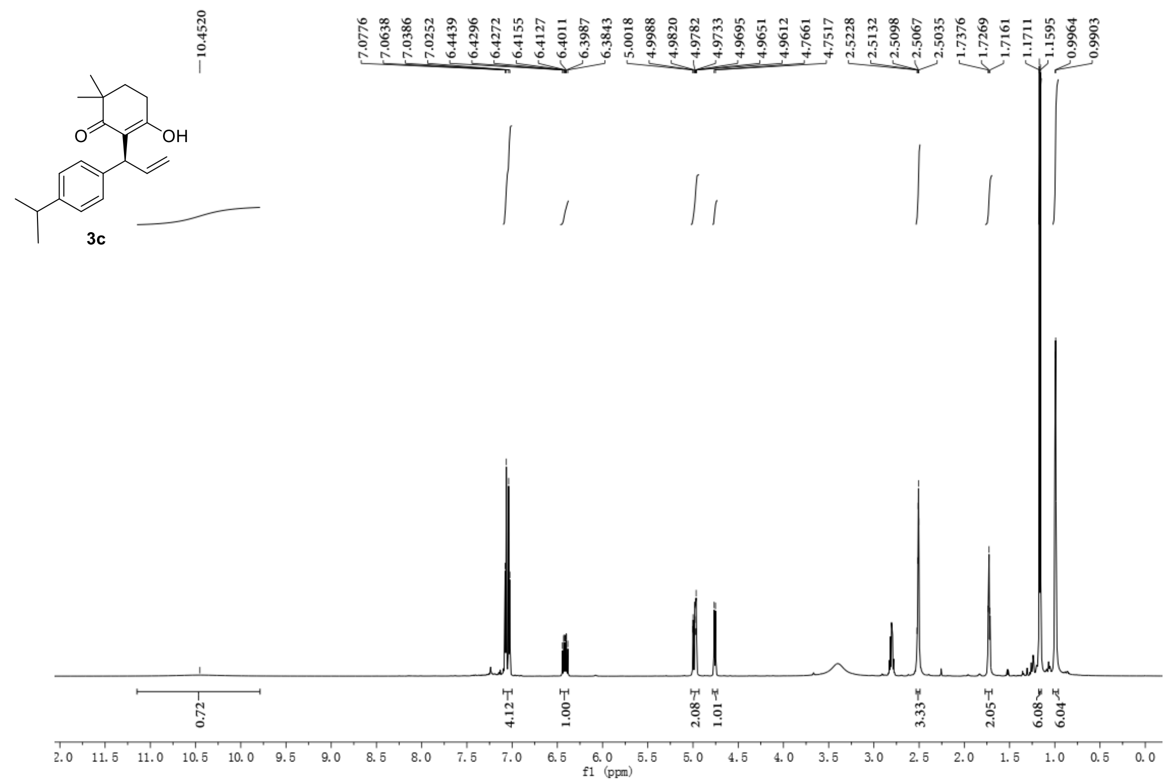
10.4520
7.0776
7.0638
7.0386
7.0252
6.4439
6.4296
6.4272
6.4155
6.4127
6.4011
6.3987
6.3843
5.0018
4.9988
4.9820
4.9782
4.9733
4.9695
4.9651
4.9612
4.7661
4.7517
2.5228
2.5132
2.5098
2.5067
2.5035
1.7376
1.7269
1.7161
1.1711
1.1595
0.9964
0.9903
O
OH
3c
0.72
4.12
1.00
2.08
1.01
3.33
2.05
6.08
6.04
f1 (ppm)

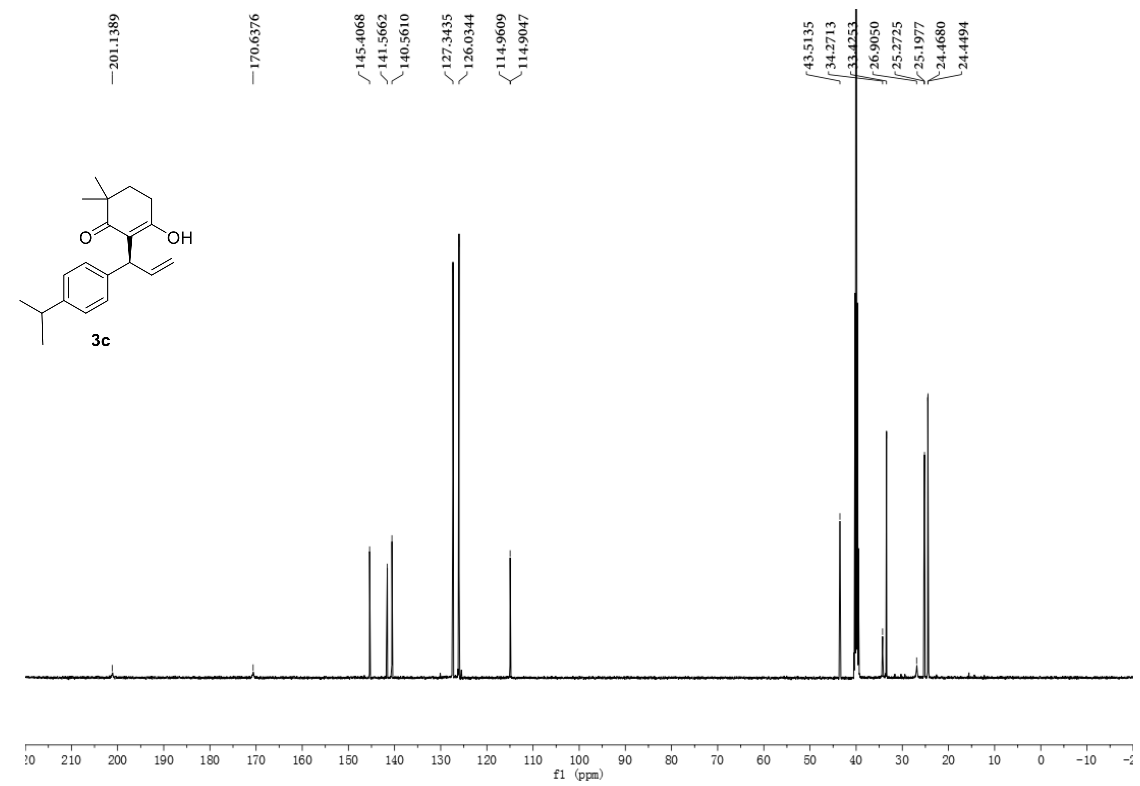
201.1389
170.6376
145.4068
141.5662
140.5610
127.3435
126.0344
114.9609
114.9047
43.5135
34.2713
33.4253
26.9050
25.2725
25.1977
24.4680
24.4494
O
OH
3c
f1 (ppm)

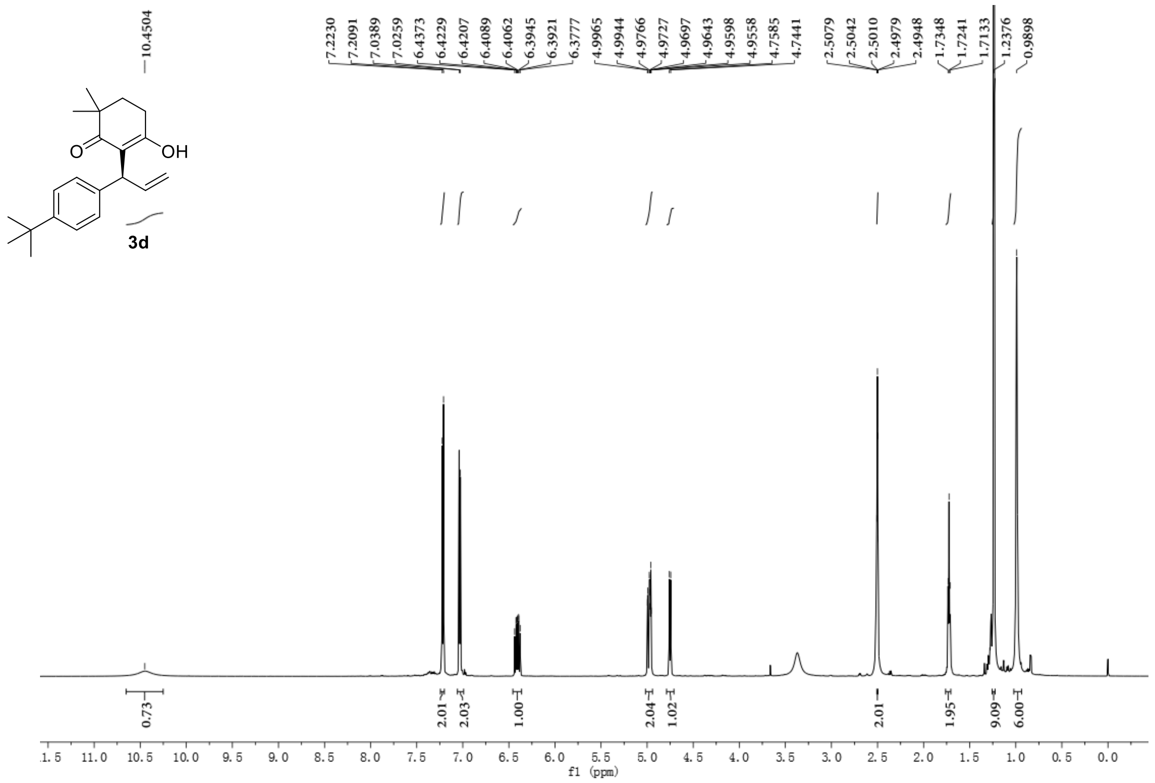
10.4504
7.2230 7.2091 7.0389 7.0259 6.4373 6.4229 6.4207 6.4089 6.4062 6.3945 6.3921 6.3777
4.9965 4.9944 4.9766 4.9727 4.9697 4.9643 4.9598 4.9558 4.7585 4.7441
2.5079 2.5042 2.5010 2.4979 2.4948 1.7348 1.7241 1.7133 1.2376 0.9898
O
OH
3d
0.73
2.01
2.03
1.00
2.04
1.02
2.01
1.95
9.09
6.00
f1 (ppm)

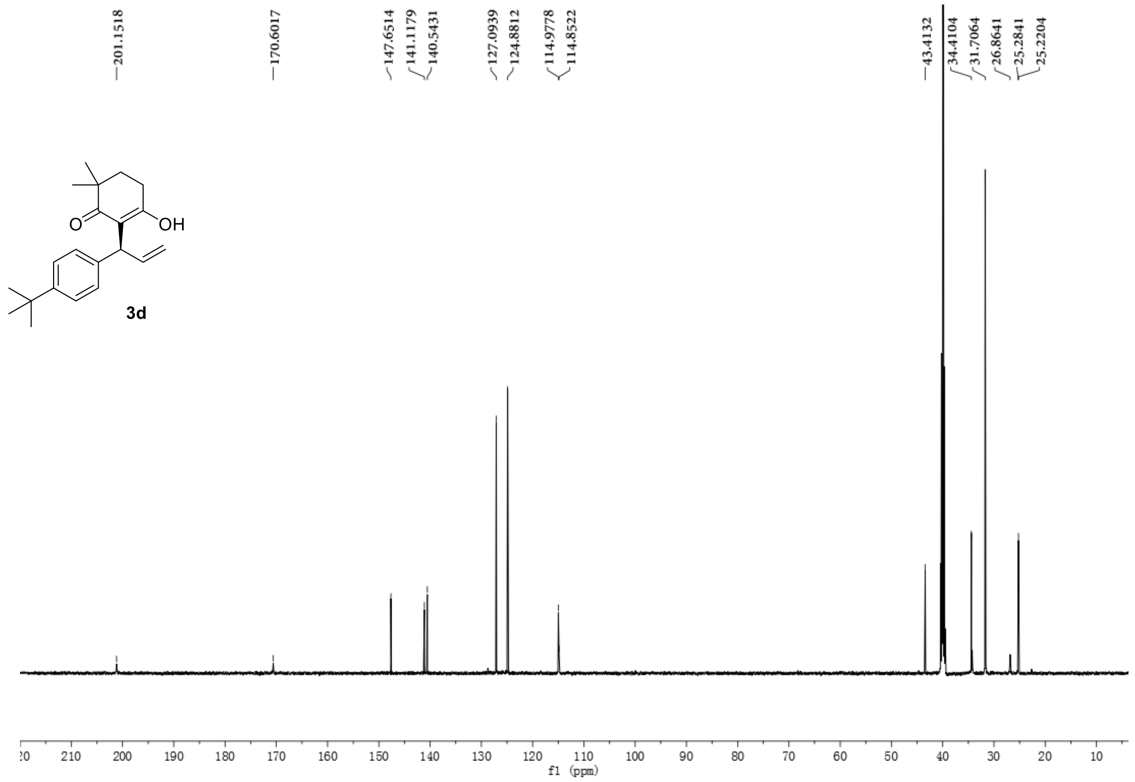
201.1518
170.6017
147.6514 141.1179 140.5431
127.0939 124.8812
114.9778 114.8522
43.4132 34.4104 31.7064 26.8641 25.2841 25.2204
O
OH
3d
f1 (ppm)

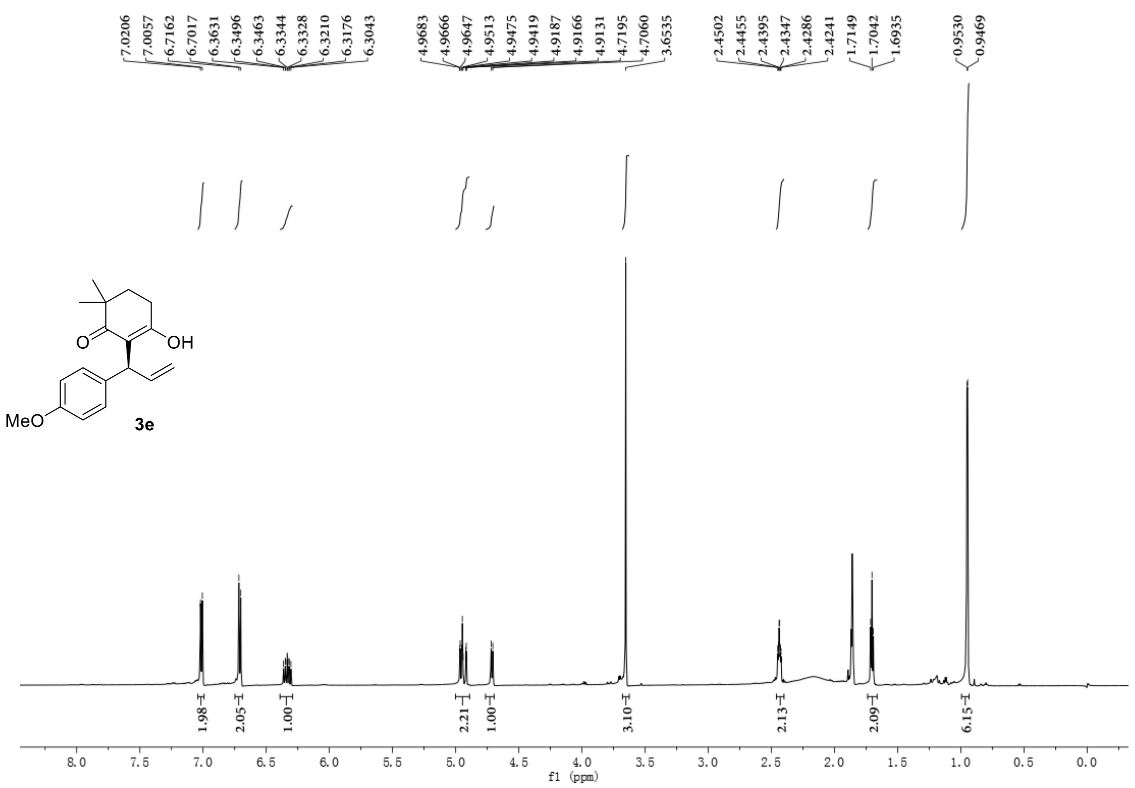
7.0206 7.0057 6.7162 6.7017 6.3631 6.3496 6.3463 6.3344 6.3328 6.3210 6.3176 6.3043
4.9683 4.9666 4.9647 4.9513 4.9475 4.9419 4.9187 4.9166 4.9131 4.7195 4.7060 3.6535
2.4502 2.4455 2.4395 2.4347 2.4286 2.4241 1.7149 1.7042 1.6935
0.9530 0.9469
O
OH
MeO
3e
1.98 2.05 1.00 2.21 1.00 3.10 2.13 2.09 6.15
8.0 7.5 7.0 6.5 6.0 5.5 5.0 4.5 4.0 3.5 3.0 2.5 2.0 1.5 1.0 0.5 0.0
f1 (ppm)

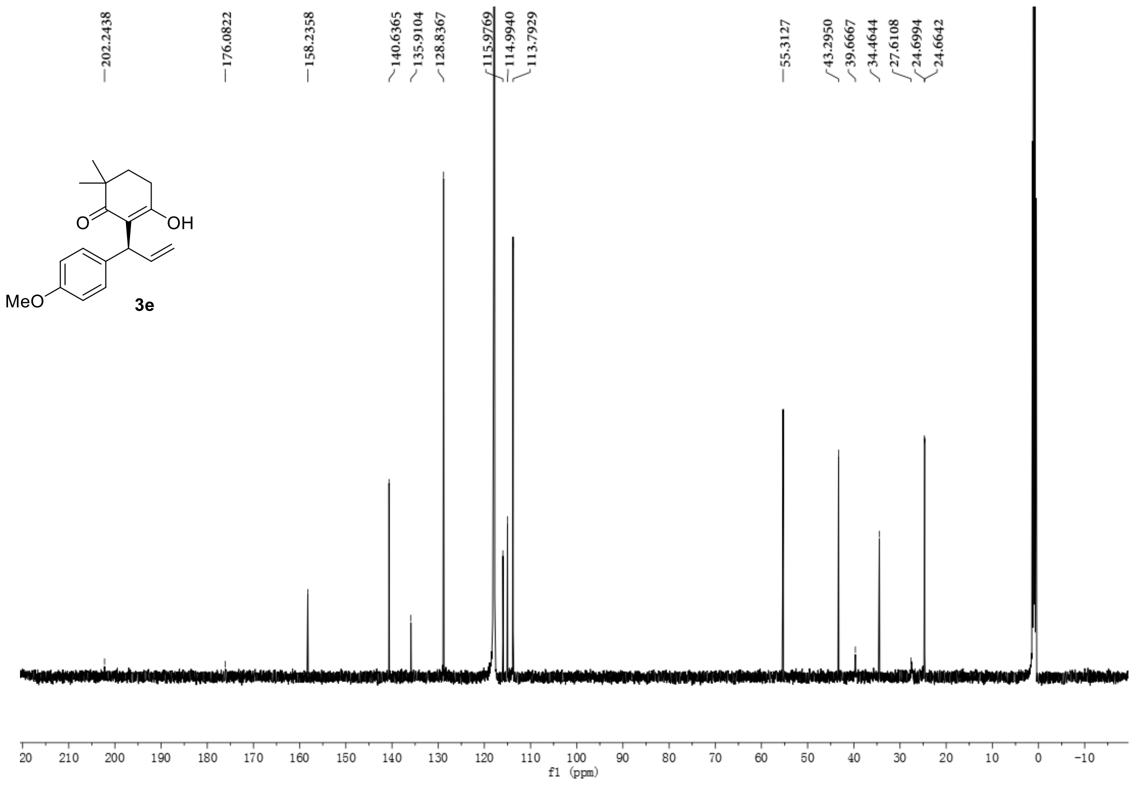
202.2438
176.0822
158.2358
140.6365 135.9104 128.8367
115.9769 114.9940 113.7929
55.3127
43.2950 39.6667 34.4644 27.6108 24.6994 24.6642
O
OH
MeO
3e
20 210 200 190 180 170 160 150 140 130 120 110 100 90 80 70 60 50 40 30 20 10 0 -10
f1 (ppm)

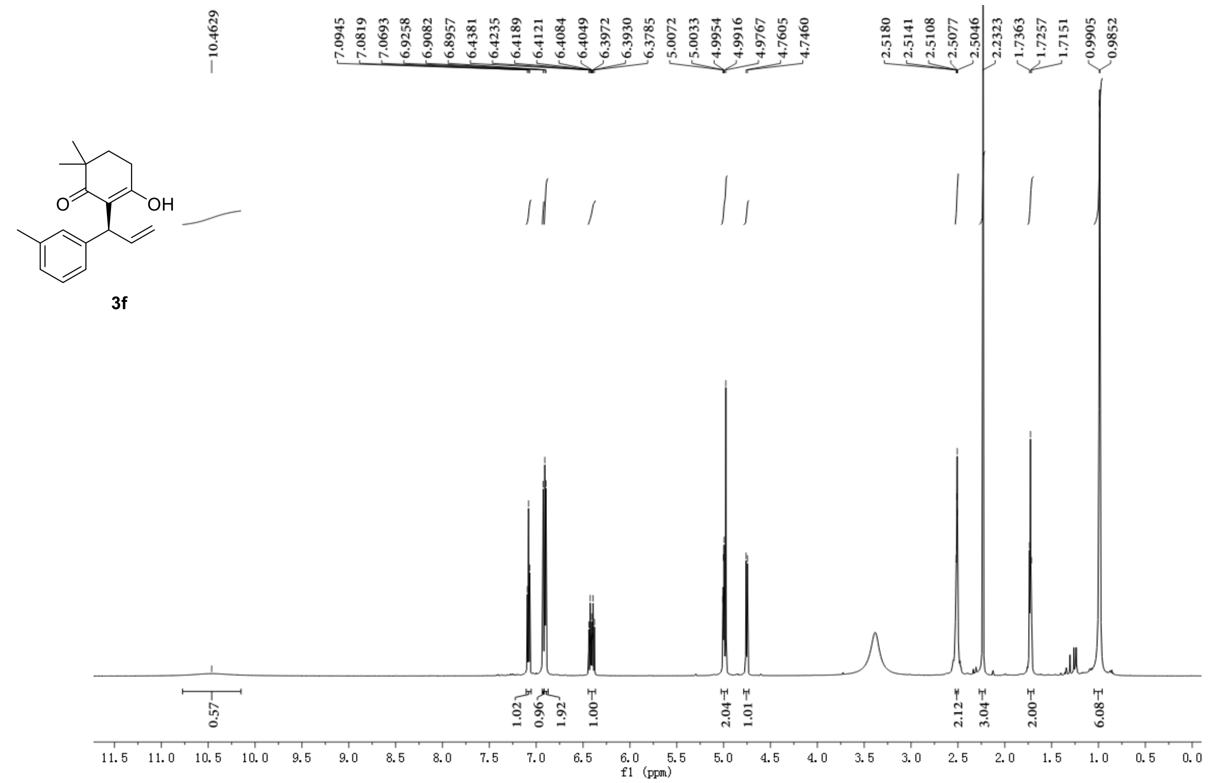

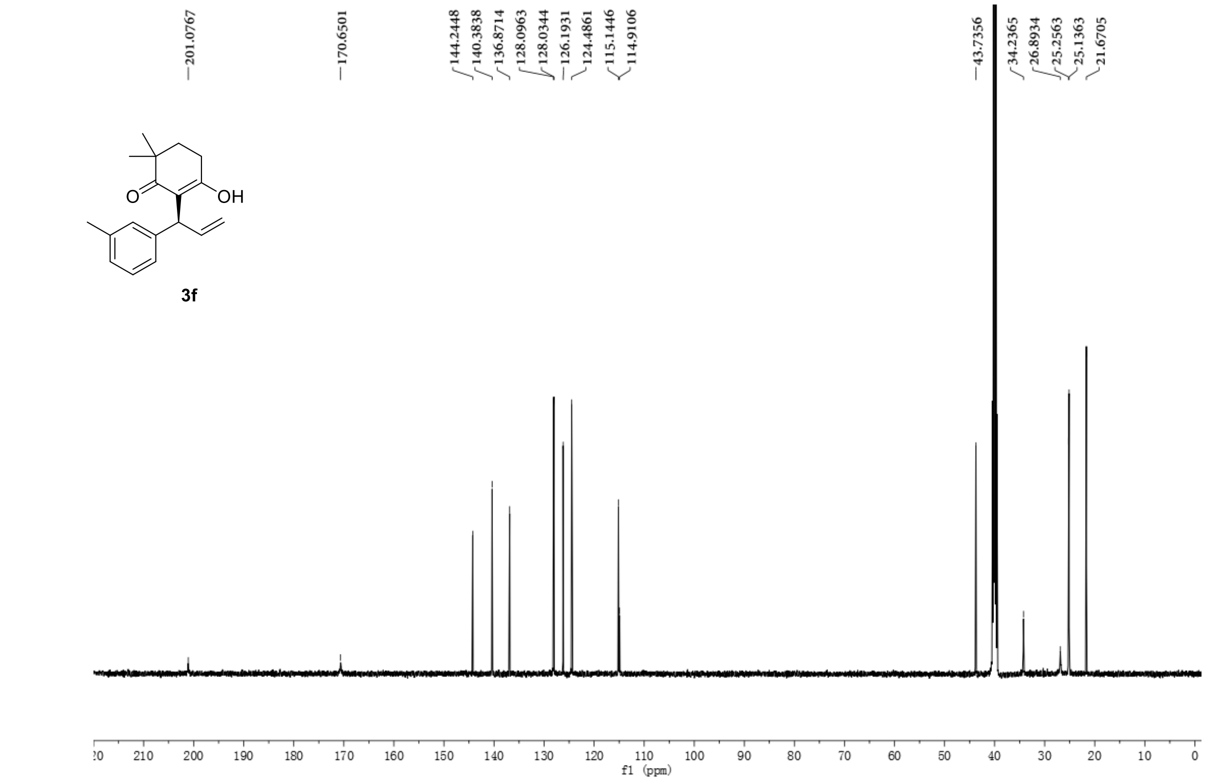

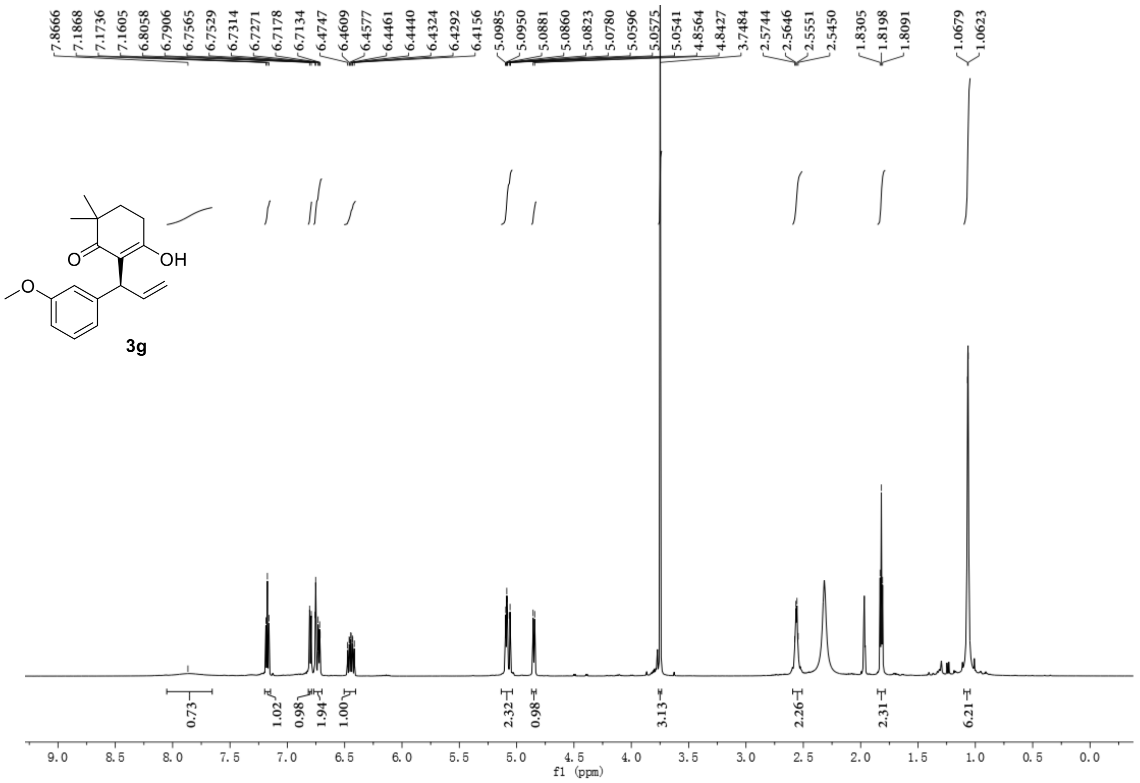

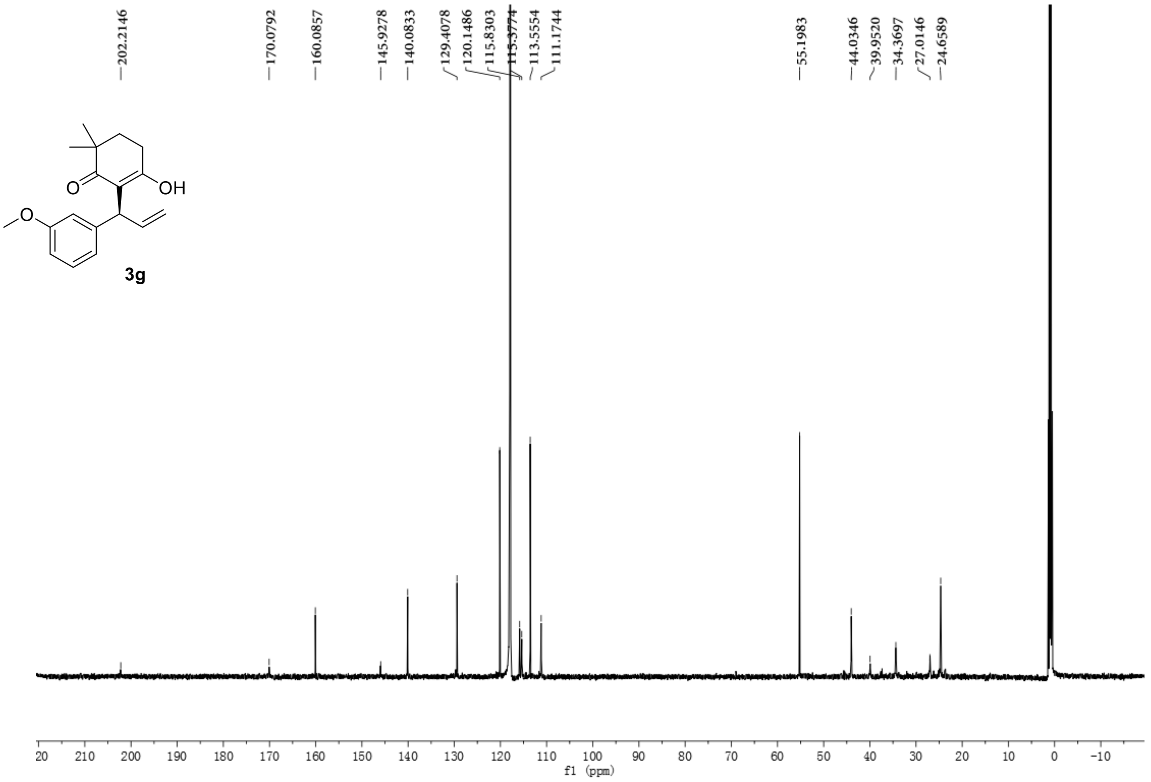

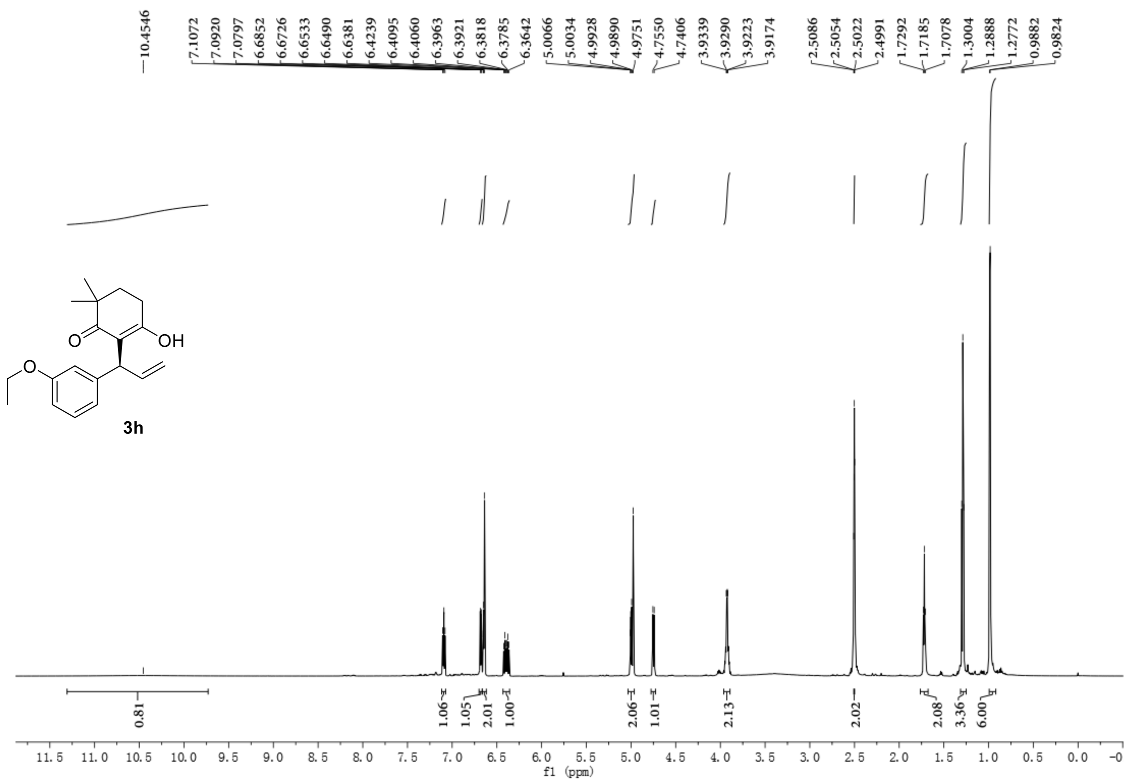

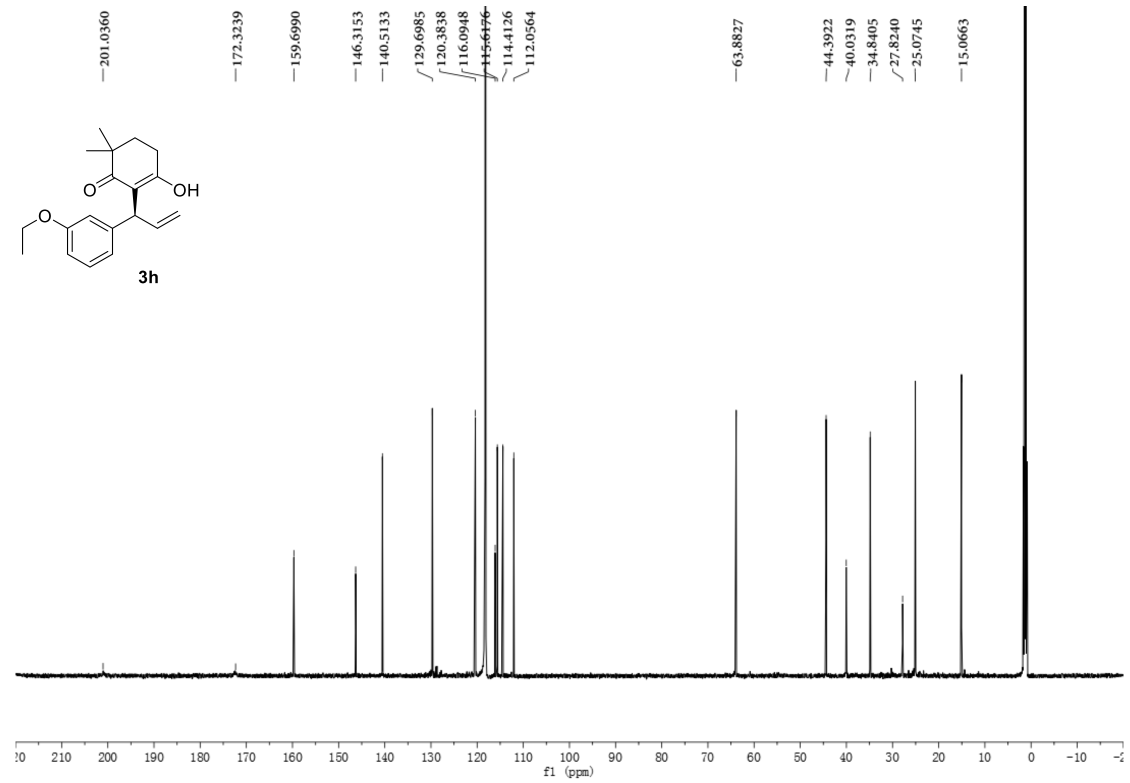

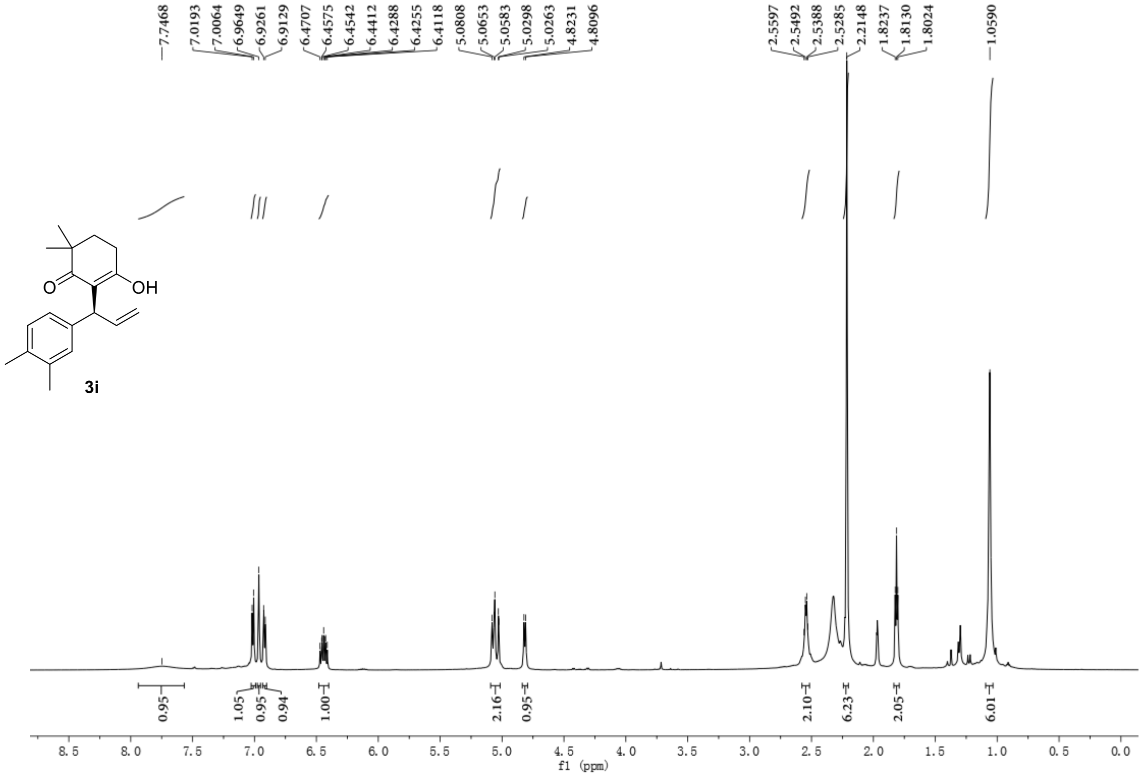
7.7468
7.0193
7.0064
6.9649
6.9261
6.9129
6.4707
6.4575
6.4542
6.4412
6.4288
6.4255
6.4118
5.0808
5.0653
5.0583
5.0298
5.0263
4.8231
4.8096
2.5597
2.5492
2.5388
2.5285
2.2148
1.8237
1.8130
1.8024
1.0590
OH
O
3i
0.95
1.05
0.95
0.94
1.00
2.16
0.95
2.10
6.23
2.05
6.01
f1 (ppm)

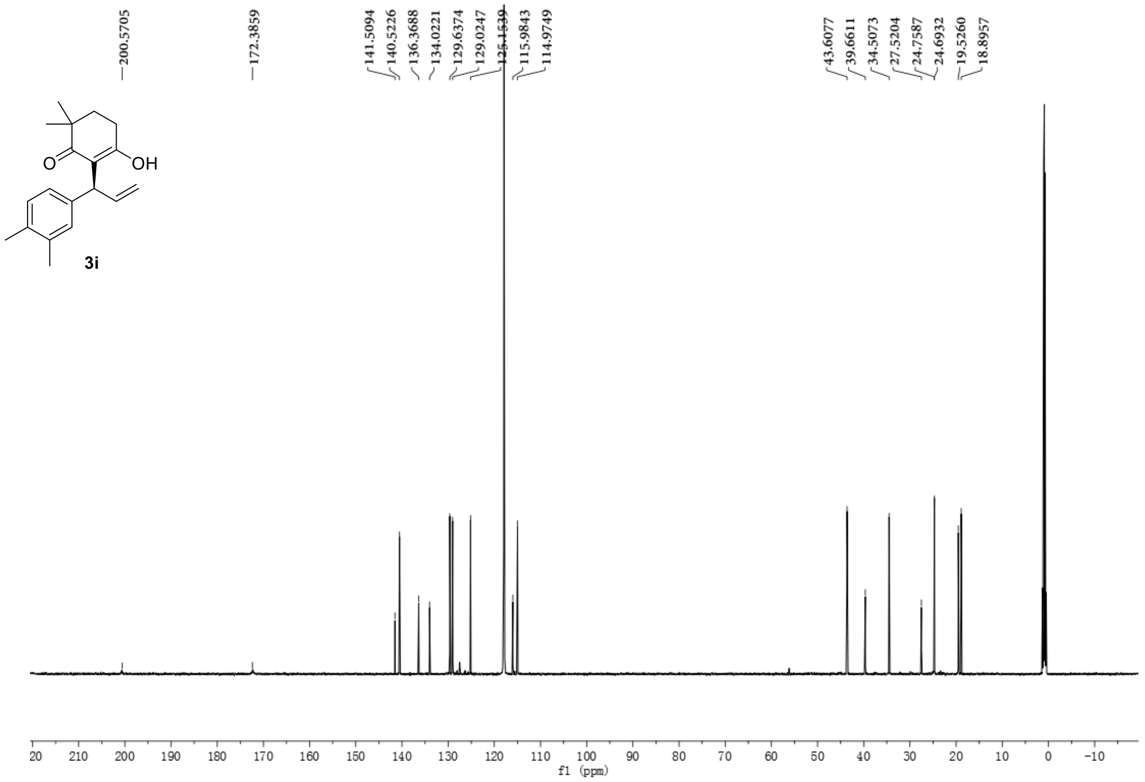
200.5705
172.3859
141.5094
140.5226
136.3688
134.0221
129.6374
129.0247
125.1539
115.9843
114.9749
43.6077
39.6611
34.5073
27.5204
24.7587
24.6932
19.5260
18.8957
OH
O
3i
f1 (ppm)

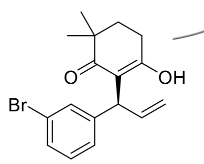

3j

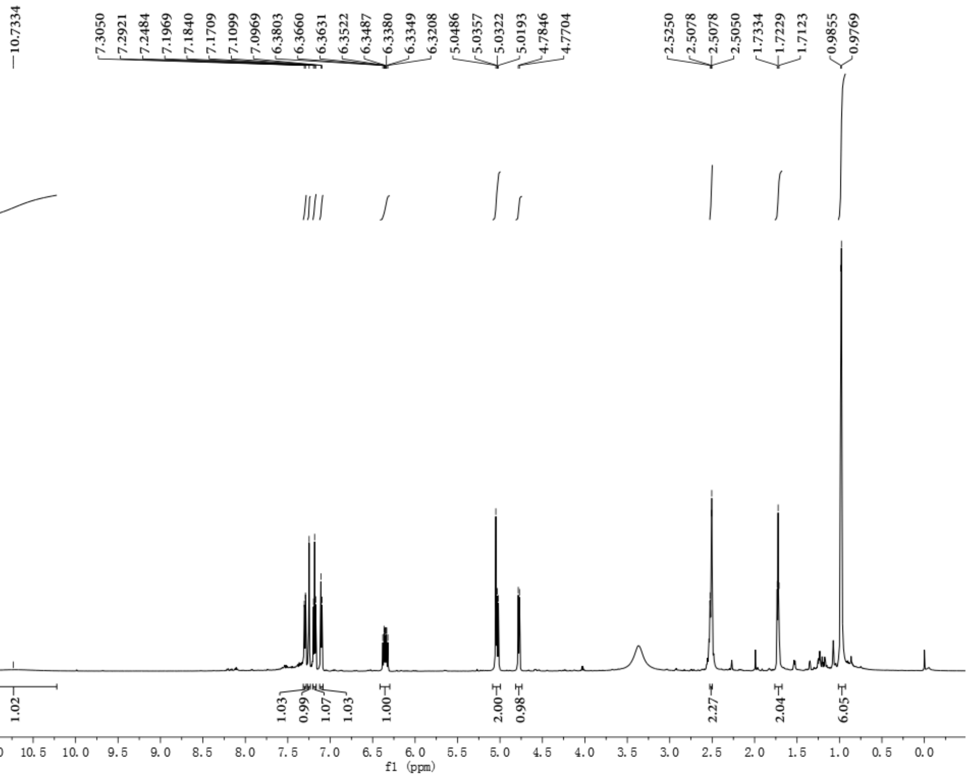

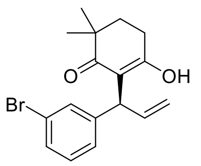

3j

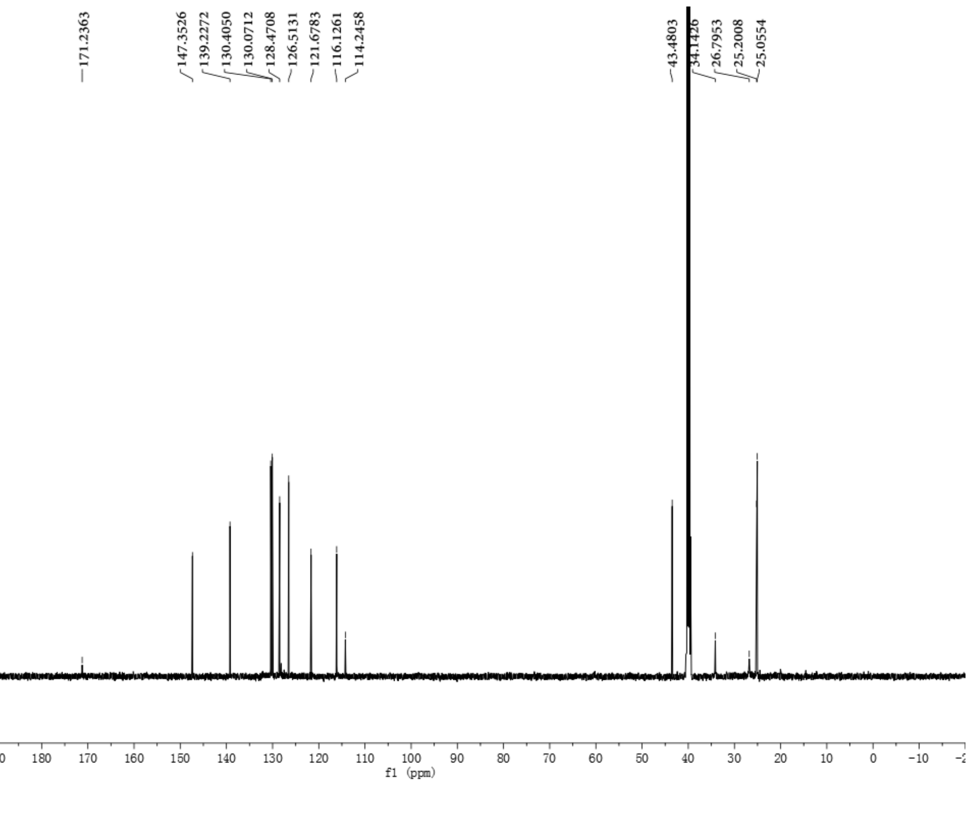

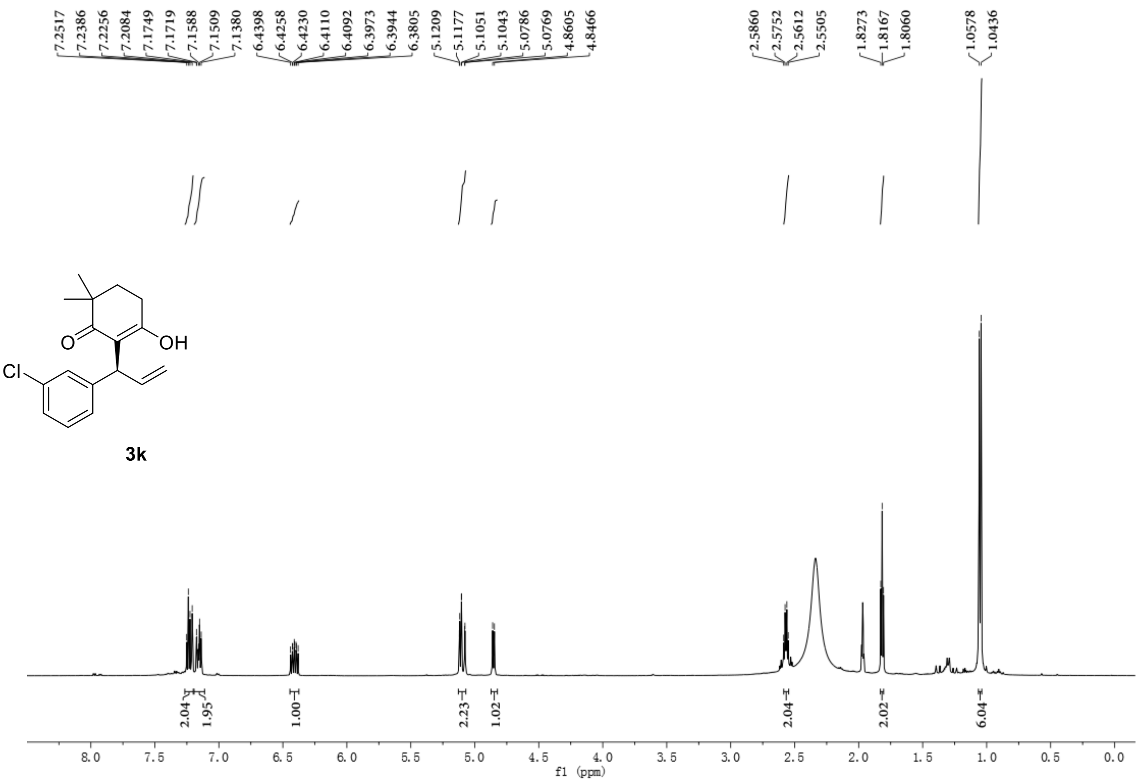

7.2517
7.2386
7.2256
7.2084
7.1749
7.1719
7.1588
7.1509
7.1380
6.4398
6.4258
6.4230
6.4110
6.4092
6.3973
6.3944
6.3805
5.1209
5.1177
5.1051
5.1043
5.0786
5.0769
4.8605
4.8466
2.5860
2.5752
2.5612
2.5505
1.8273
1.8167
1.8060
1.0578
1.0436
O
OH
Cl
3k
2.04
1.95
1.00
2.23
1.02
2.04
2.02
6.04
8.0 7.5 7.0 6.5 6.0 5.5 5.0 4.5 4.0 3.5 3.0 2.5 2.0 1.5 1.0 0.5 0.0
f1 (ppm)

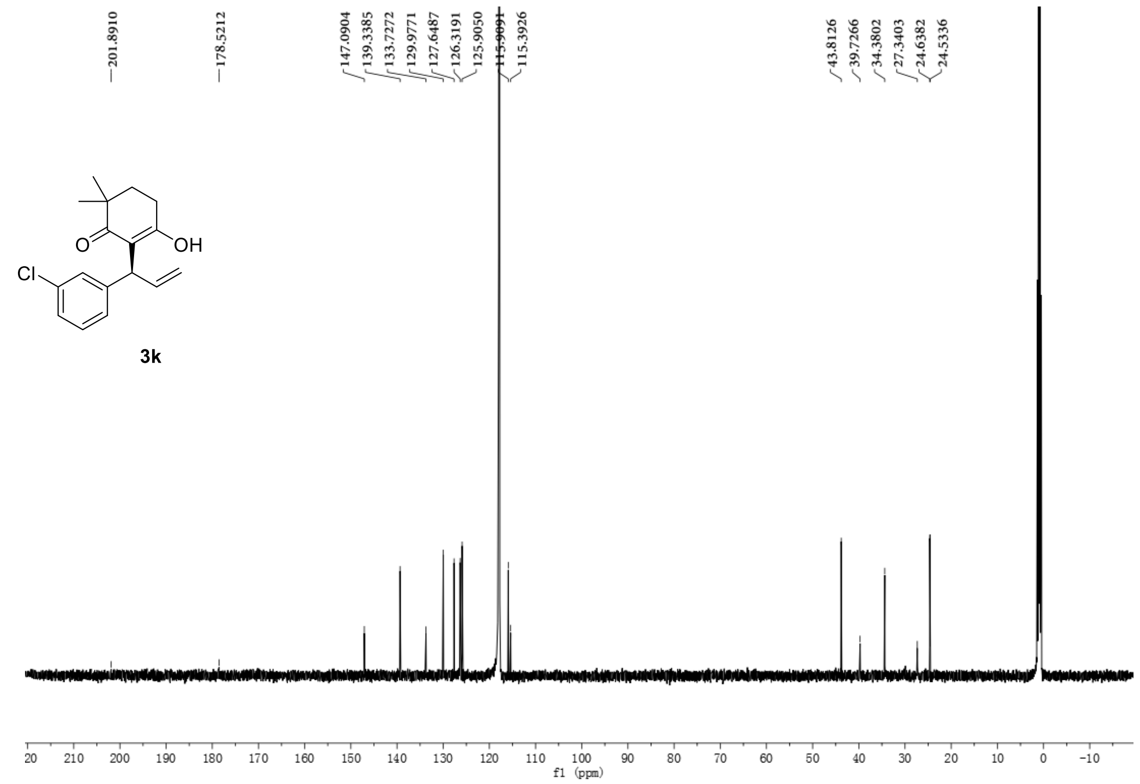

201.8910
178.5212
147.0904
139.3385
133.7272
129.9771
127.6487
126.3191
125.9050
115.9891
115.3926
43.8126
39.7266
34.3802
27.3403
24.6382
24.5336
O
OH
Cl
3k
20 210 200 190 180 170 160 150 140 130 120 110 100 90 80 70 60 50 40 30 20 10 0 -10
f1 (ppm)

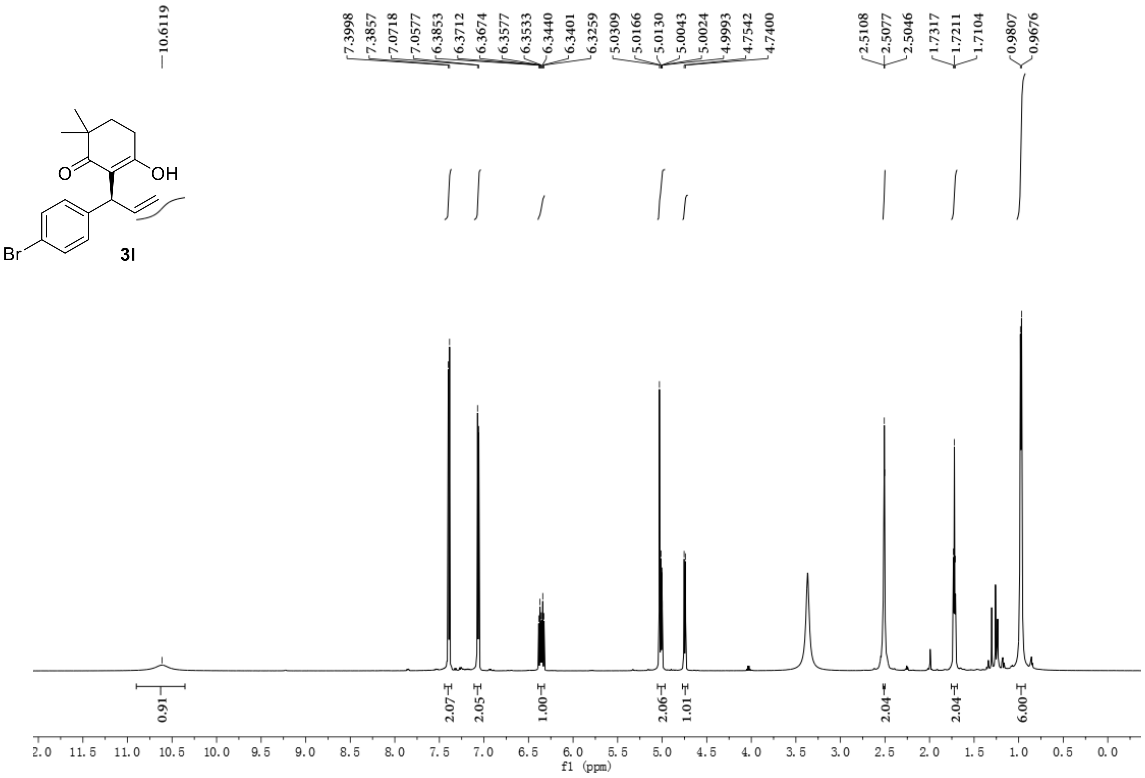
10.6119
7.3998
7.3857
7.0718
7.0577
6.3853
6.3712
6.3674
6.3577
6.3533
6.3440
6.3401
6.3259
5.0309
5.0166
5.0130
5.0043
5.0024
4.9993
4.7542
4.7400
2.5108
2.5077
2.5046
1.7317
1.7211
1.7104
0.9807
0.9676
O
OH
Br
3l
0.91
2.07
2.05
1.00
2.06
1.01
2.04
2.04
6.00
f1 (ppm)

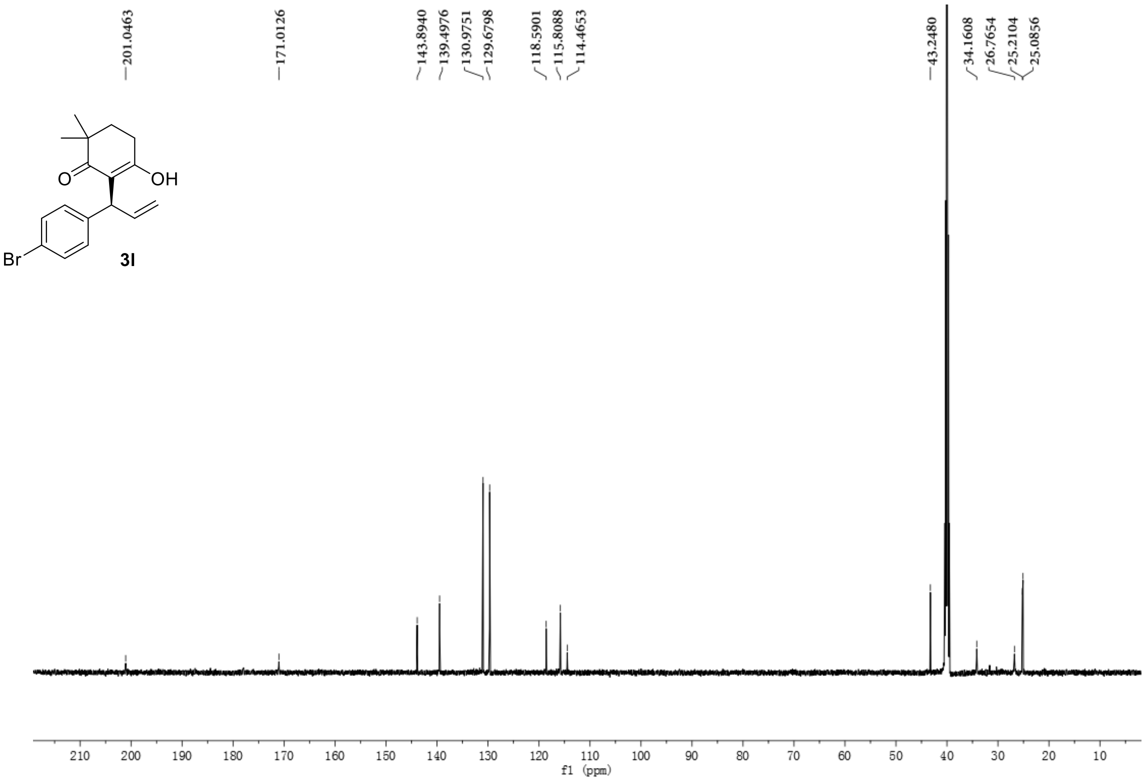
201.0463
171.0126
143.8940
139.4976
130.9751
129.6798
118.5901
115.8088
114.4653
43.2480
34.1608
26.7654
25.2104
25.0856
O
OH
Br
3l
f1 (ppm)

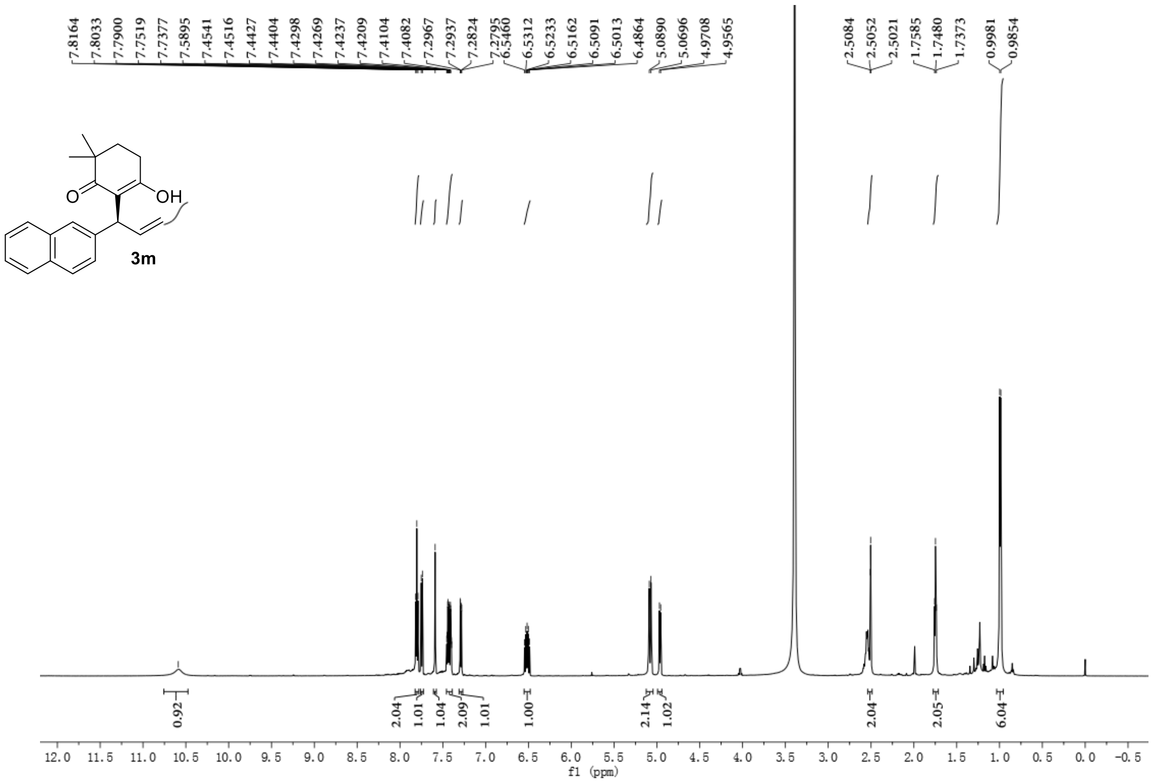
3m
f1 (ppm)

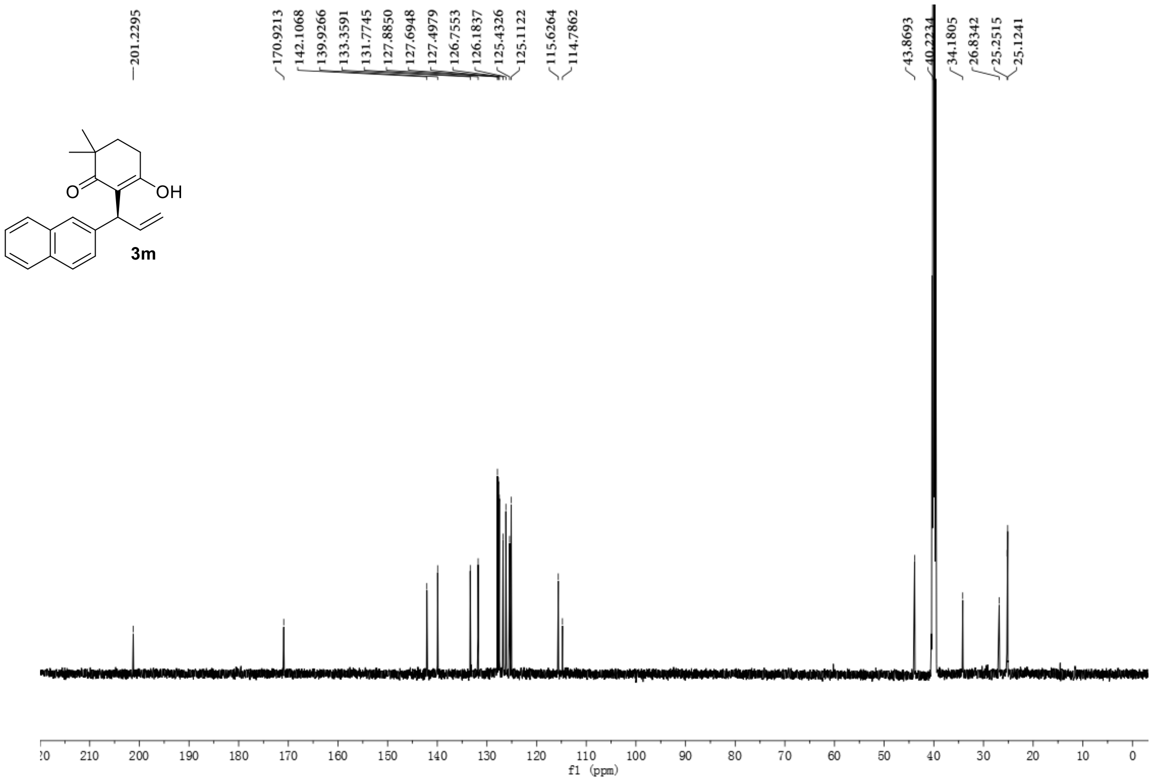
3m
f1 (ppm)

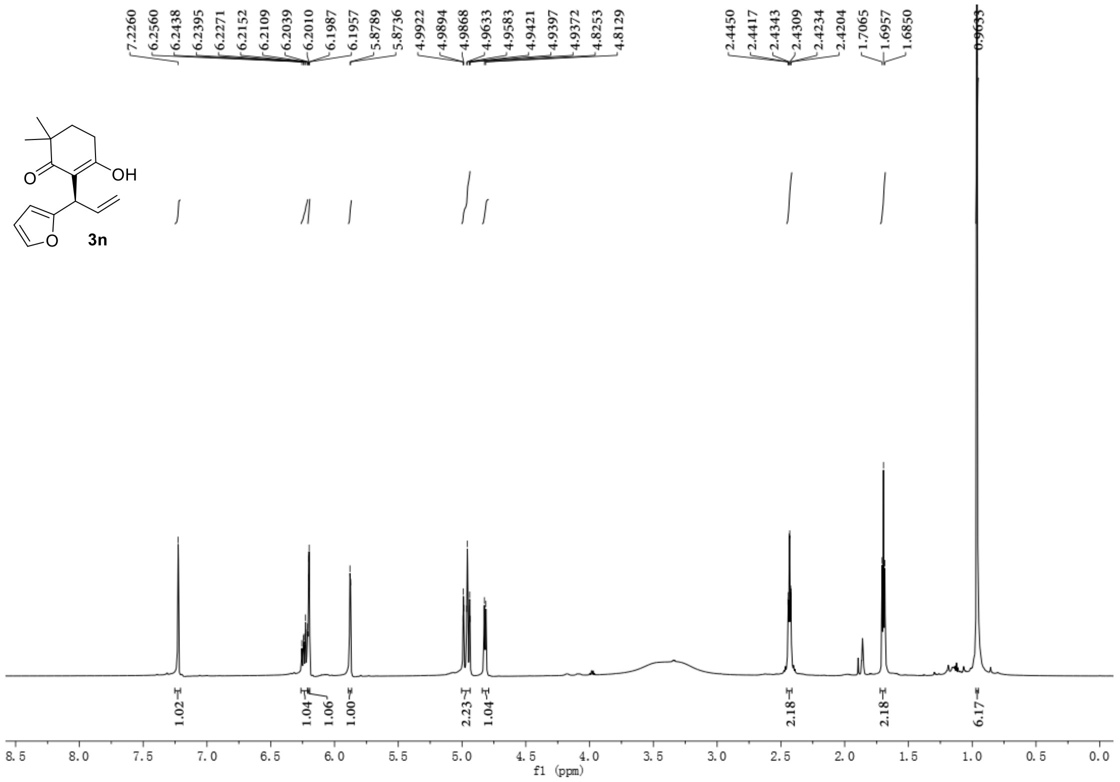
7.2260 6.2560 6.2438 6.2395 6.2271 6.2152 6.2109 6.2039 6.2010 6.1987 6.1957 5.8789 5.8736 4.9922 4.9894 4.9868 4.9633 4.9583 4.9421 4.9397 4.9372 4.8253 4.8129
2.4450 2.4417 2.4343 2.4309 2.4234 2.4204 1.7065 1.6957 1.6850 0.9633
O
OH
O
3n
1.02 1.04 1.06 1.00 2.23 1.04 2.18 2.18 6.17
8.5 8.0 7.5 7.0 6.5 6.0 5.5 5.0 4.5 4.0 3.5 3.0 2.5 2.0 1.5 1.0 0.5 0.0
f1 (ppm)

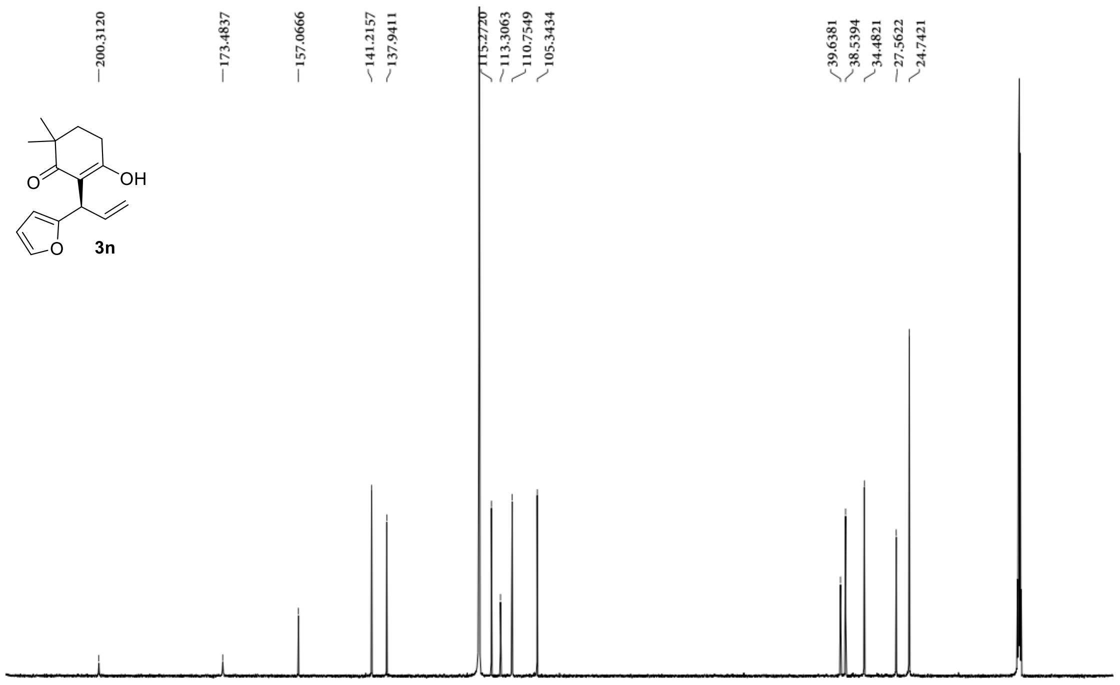
200.3120 173.4837 157.0666 141.2157 137.9411 115.2720 113.3063 110.7549 105.3434
39.6381 38.5394 34.4821 27.5622 24.7421
O
OH
O
3n
20 210 200 190 180 170 160 150 140 130 120 110 100 90 80 70 60 50 40 30 20 10 0 -10
f1 (ppm)

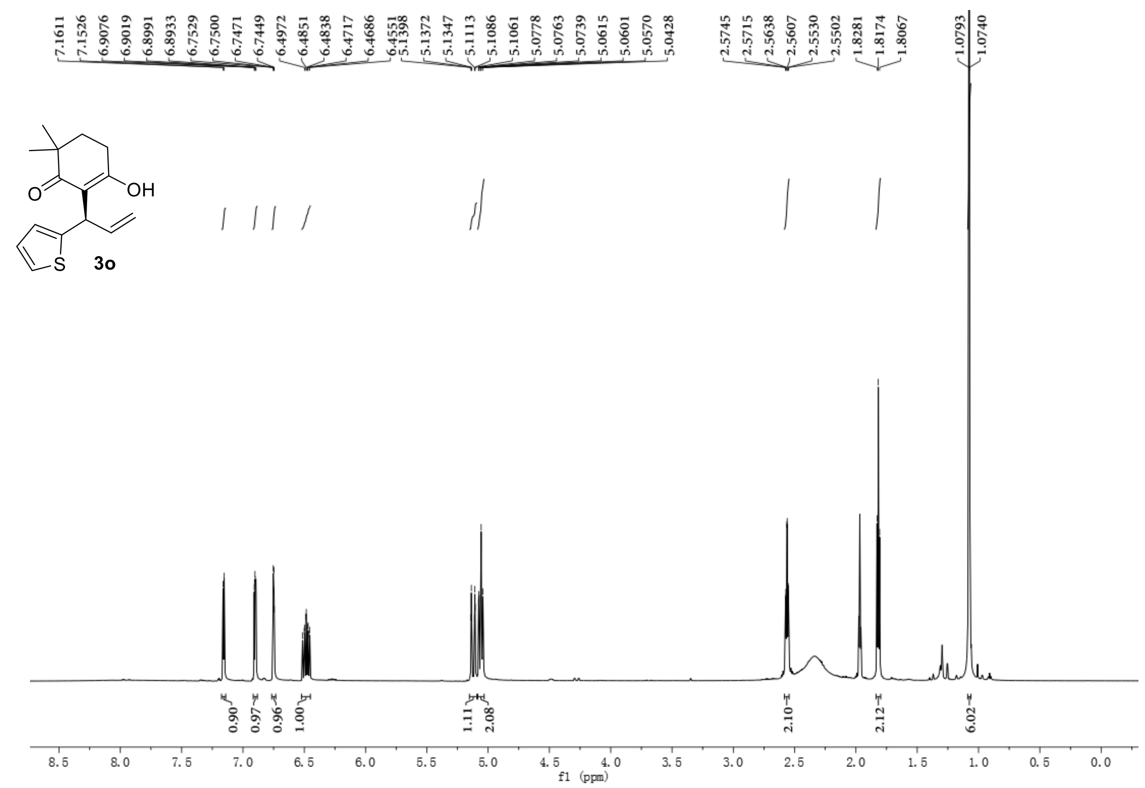
7.1611
7.1526
6.9076
6.9019
6.8991
6.8933
6.7529
6.7500
6.7471
6.7449
6.4972
6.4851
6.4838
6.4717
6.4686
6.4551
5.1398
5.1372
5.1347
5.1113
5.1086
5.1061
5.0778
5.0763
5.0739
5.0615
5.0601
5.0570
5.0428
2.5745
2.5715
2.5638
2.5607
2.5530
2.5502
1.8281
1.8174
1.8067
1.0793
1.0740
OH
O
S
3o
0.90
0.97
0.96
1.00
1.11
2.08
2.10
2.12
6.02
f1 (ppm)

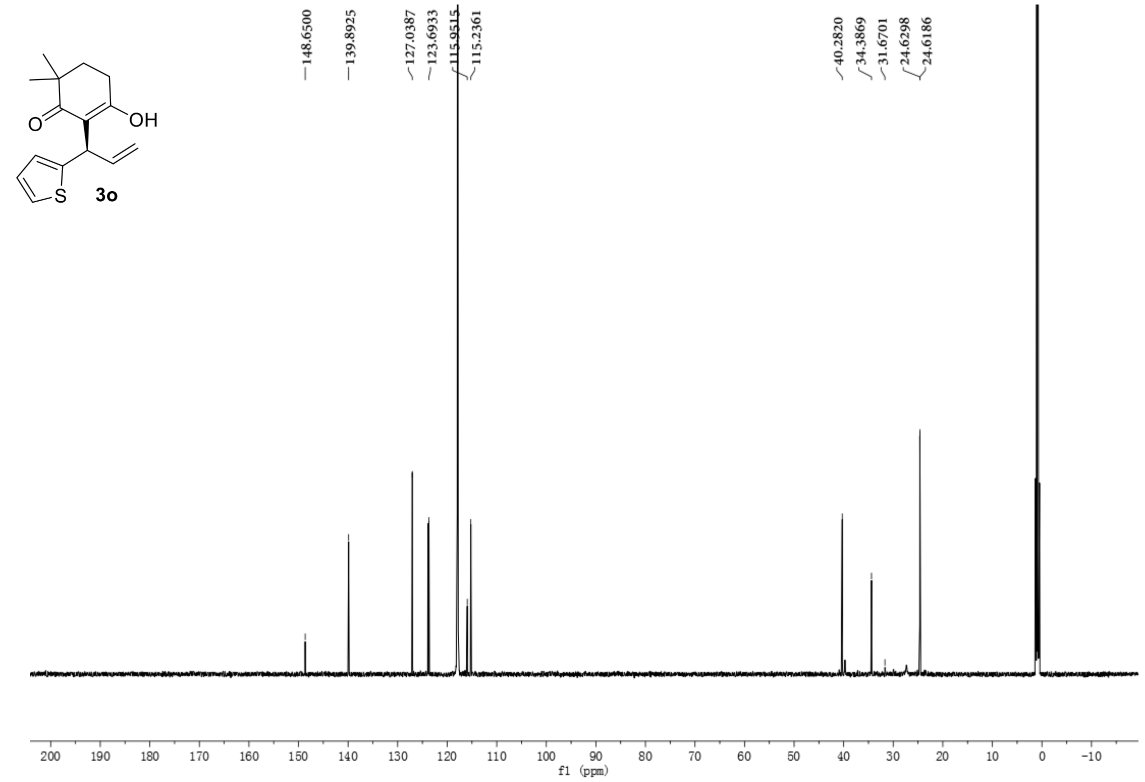
148.6500
139.8925
127.0387
123.6933
115.9515
115.2361
40.2820
34.3869
31.6701
24.6298
24.6186
OH
O
S
3o
f1 (ppm)

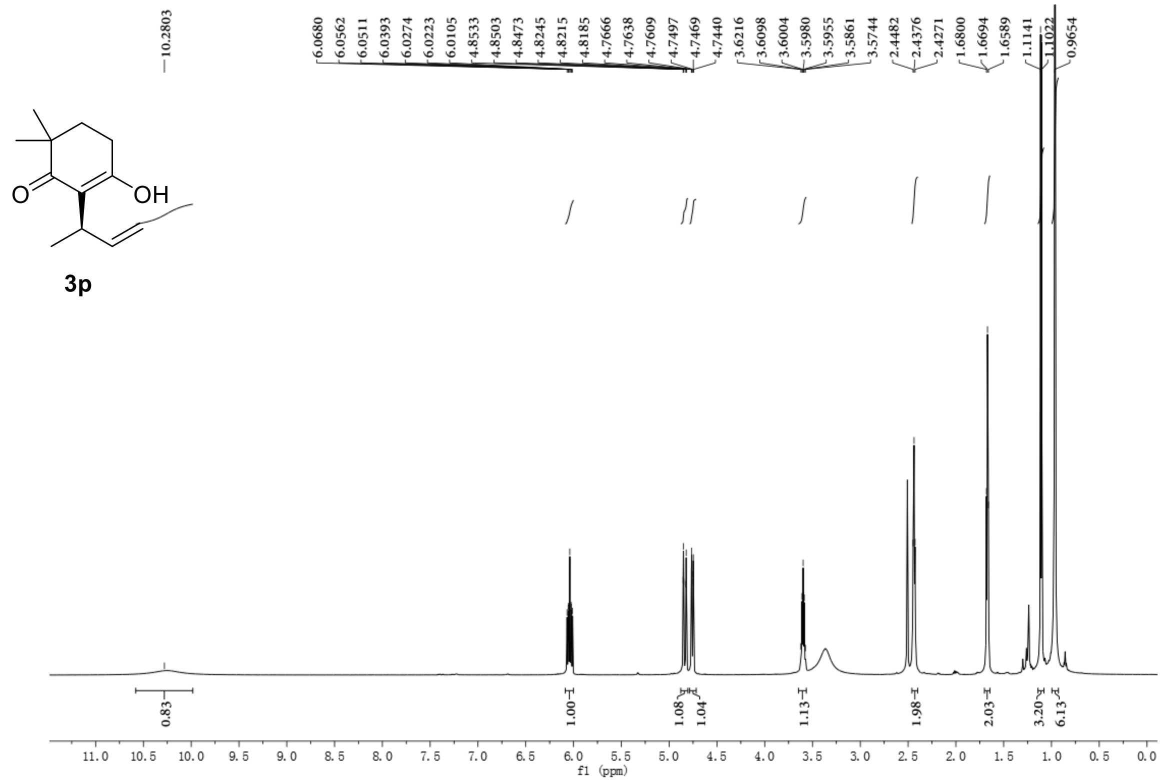
3p
O
OH
f1 (ppm)

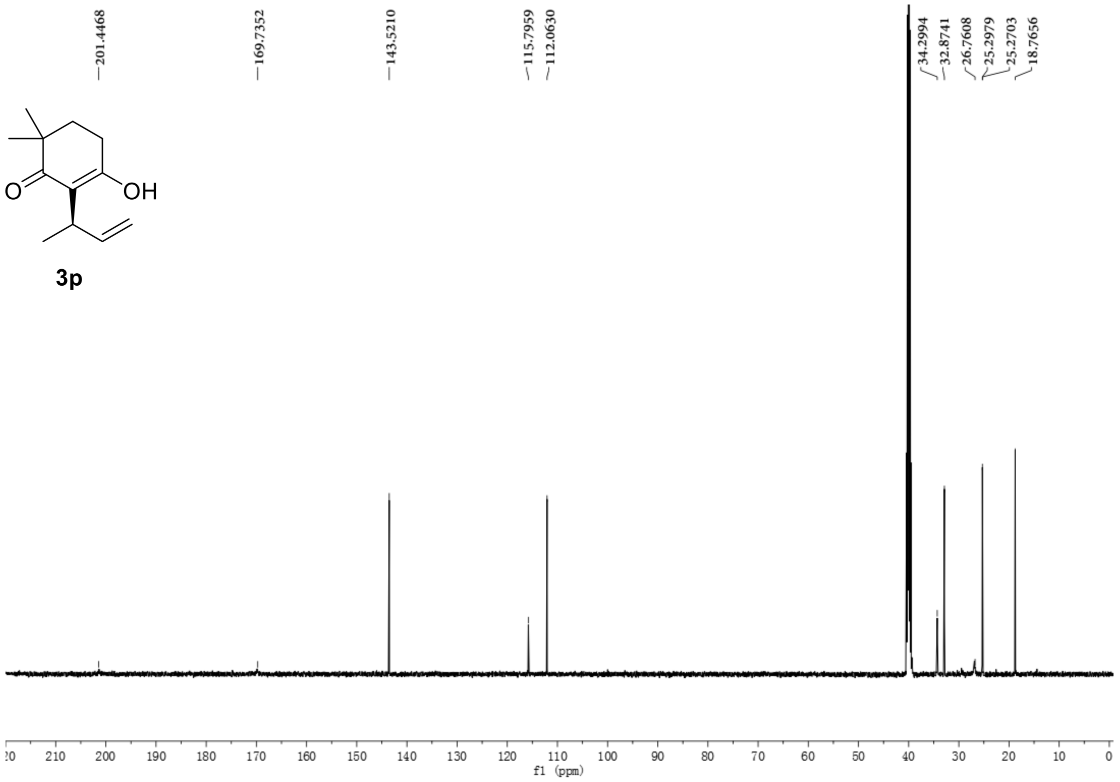
3p
O
OH
f1 (ppm)

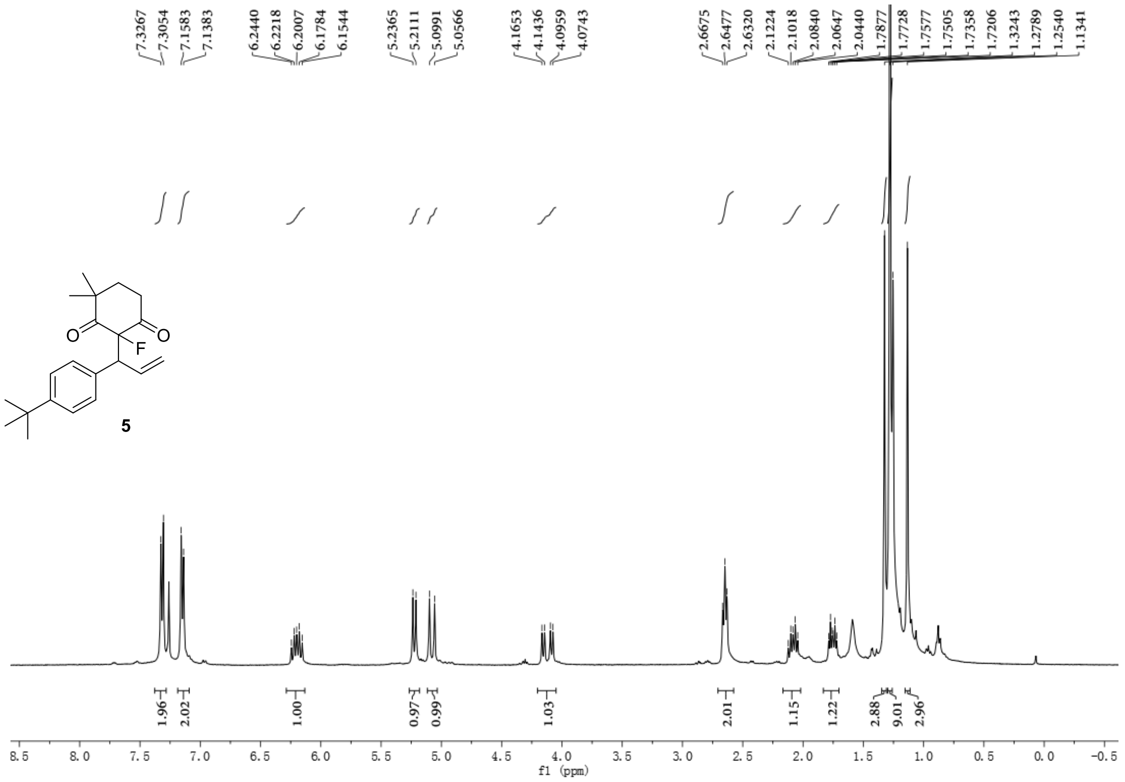
7.3267
7.3054
7.1583
7.1383
6.2440
6.2218
6.2007
6.1784
6.1544
5.2365
5.2111
5.0991
5.0566
4.1653
4.1436
4.0959
4.0743
2.6675
2.6477
2.6320
2.1224
2.1018
2.0840
2.0647
2.0440
1.7877
1.7728
1.7577
1.7505
1.7358
1.7206
1.3243
1.2789
1.2540
1.1341
O
F
O
5
1.96
2.02
1.00
0.97
0.99
1.03
2.01
1.15
1.22
2.88
9.01
2.96
8.5 8.0 7.5 7.0 6.5 6.0 5.5 5.0 4.5 4.0 3.5 3.0 2.5 2.0 1.5 1.0 0.5 0.0 -0.5
f1 (ppm)

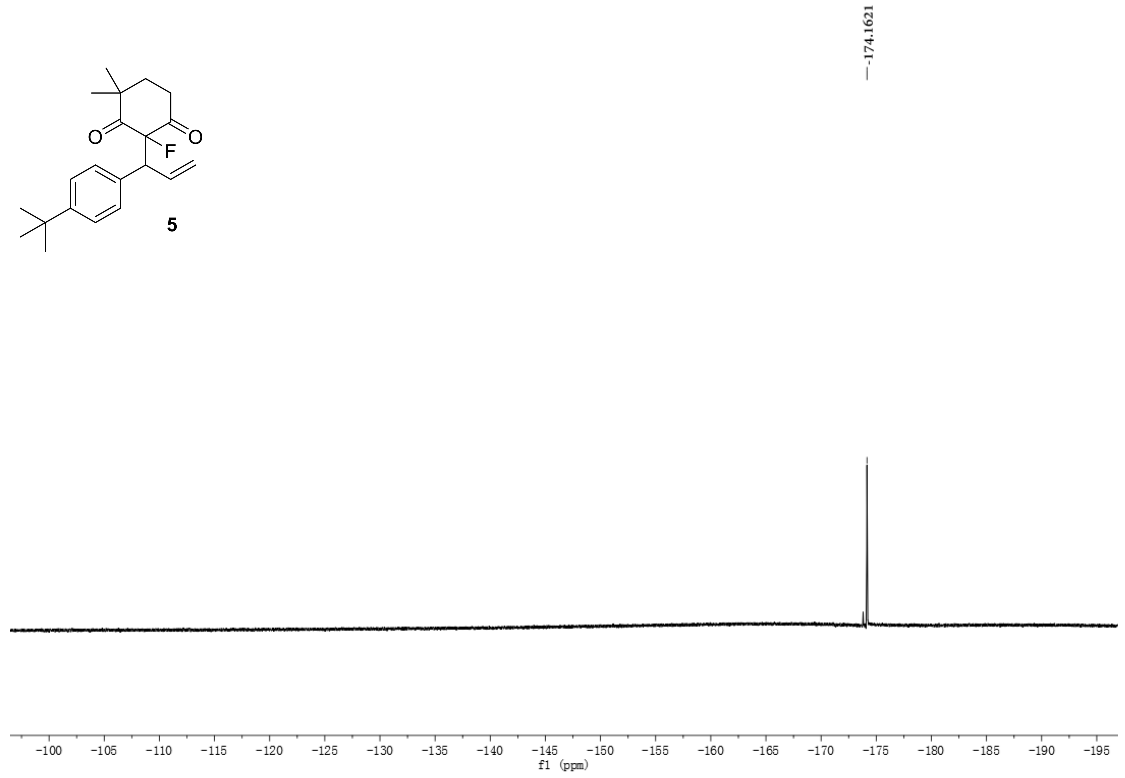
-174.1621
O
F
O
5
-100 -105 -110 -115 -120 -125 -130 -135 -140 -145 -150 -155 -160 -165 -170 -175 -180 -185 -190 -195
f1 (ppm)

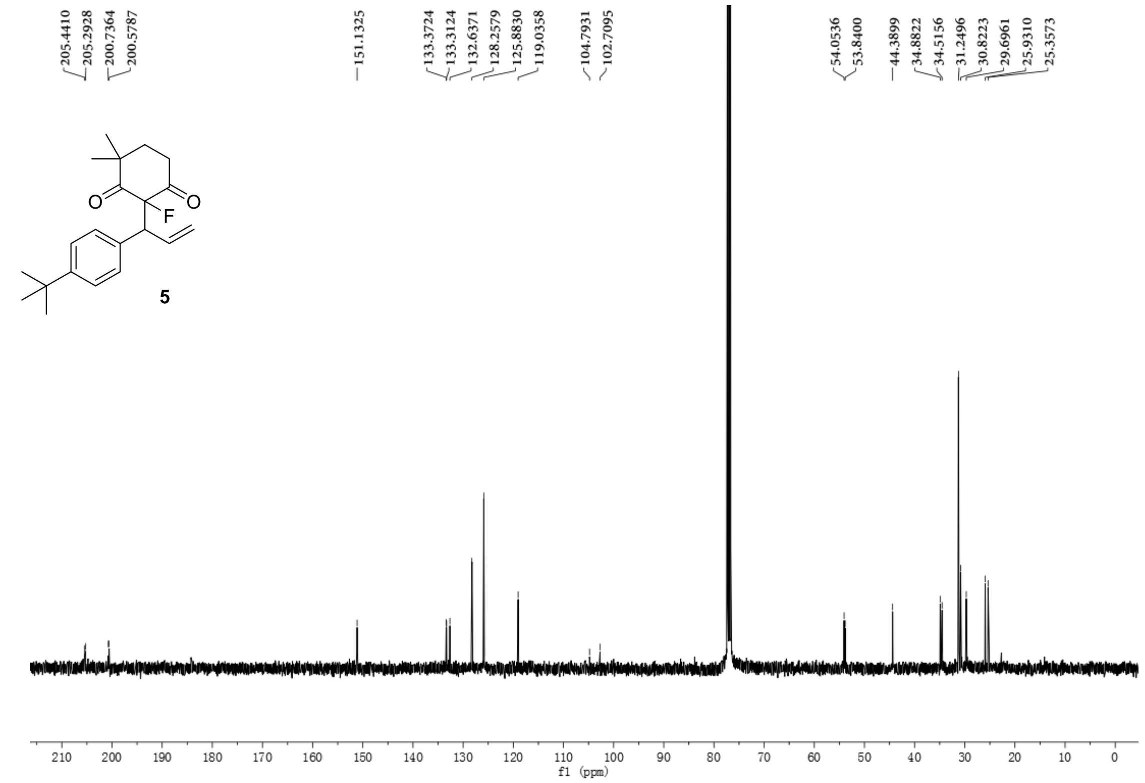
205.4410
205.2928
200.7364
200.5787
151.1325
133.3724
133.3124
132.6371
128.2579
125.8830
119.0358
104.7931
102.7095
54.0536
53.8400
44.3899
34.8822
34.5156
31.2496
30.8223
29.6961
25.9310
25.3573
O
O
F
5
210 200 190 180 170 160 150 140 130 120 110 100 90 80 70 60 50 40 30 20 10 0
f1 (ppm)

7. HPLC Spectra of compound 3 and 5

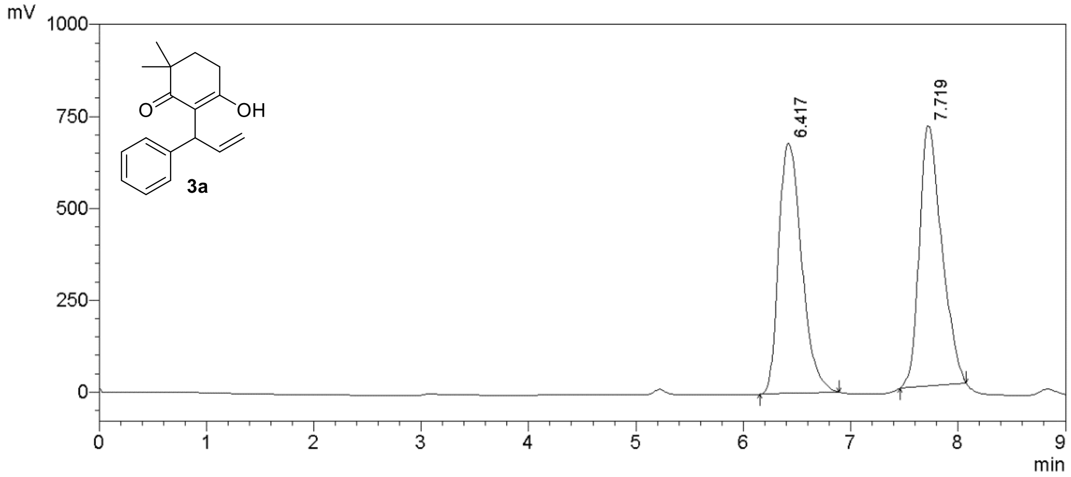

Peak No.	R. Time(min)	Peak Area(mV*min)	Percent (%)
1	6.417	10142311	49.649
2	7.719	10285810	50.351
**Total**		20428121	100.000

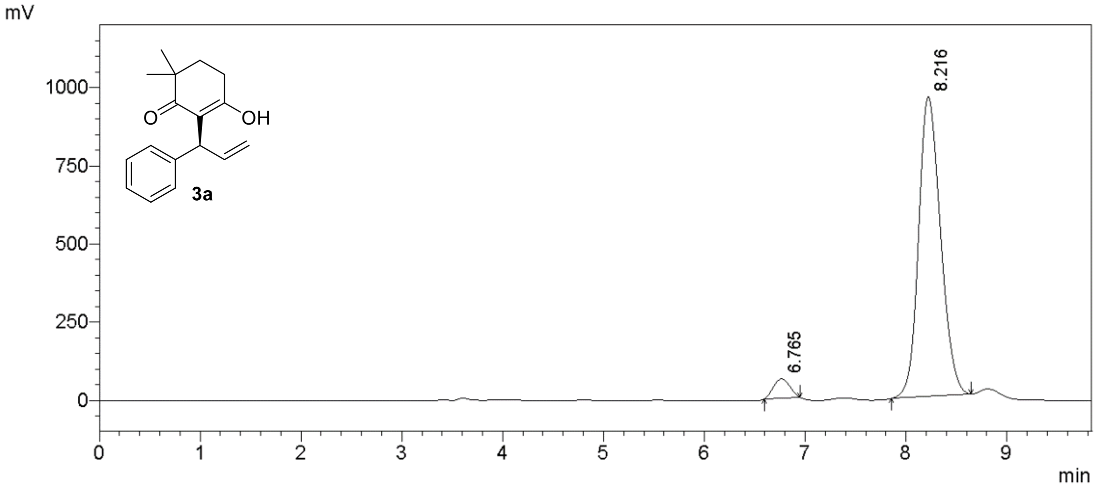

Peak No.	R. Time(min)	Peak Area(mV*min)	Percent (%)
1	6.765	682551	4.520
2	8.216	14417491	95.480
**Total**		15100041	100.000

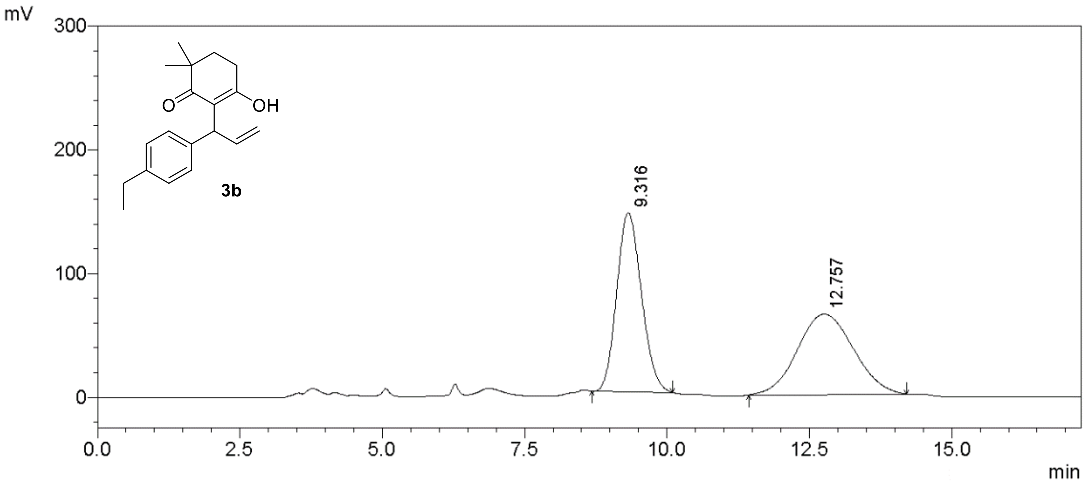

Peak No.	R. Time(min)	Peak Area(mV*min)	Percent (%)
1	9.316	4389402	49.566
2	12.757	4466251	50.434
**Total**		8855653	100.000

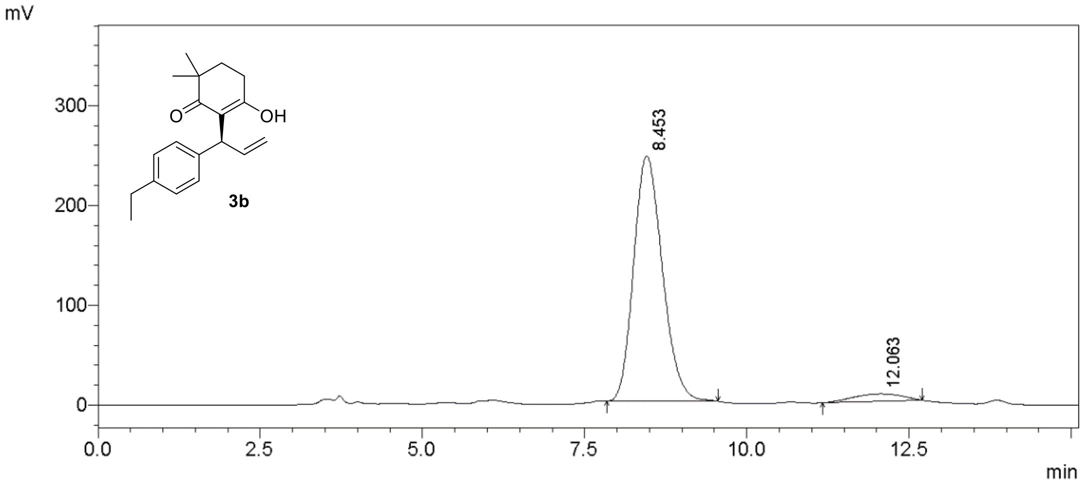

Peak No.	R. Time(min)	Peak Area(mV*min)	Percent (%)
1	8.453	7576165	95.075
2	12.063	392446	4.925
**Total**		7968611	100.000

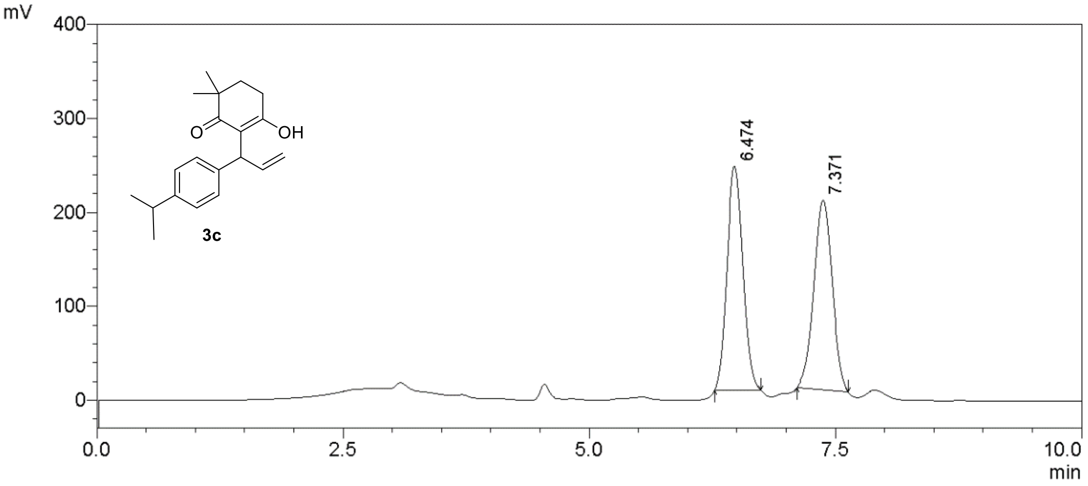

Peak No.	R. Time(min)	Peak Area(mV*min)	Percent (%)
1	6.474	2698768	50.918
2	7.371	2601480	49.082
**Total**		5300248	100.000

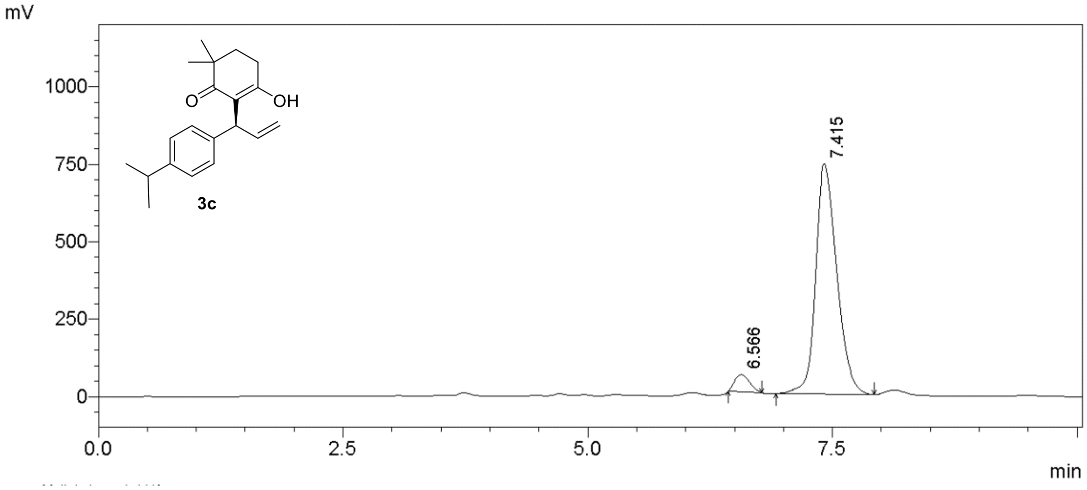

Peak No.	R. Time(min)	Peak Area(mV*min)	Percent (%)
1	6.564	584142	4.926
2	7.413	11275340	95.074
**Total**		11859483	100.000

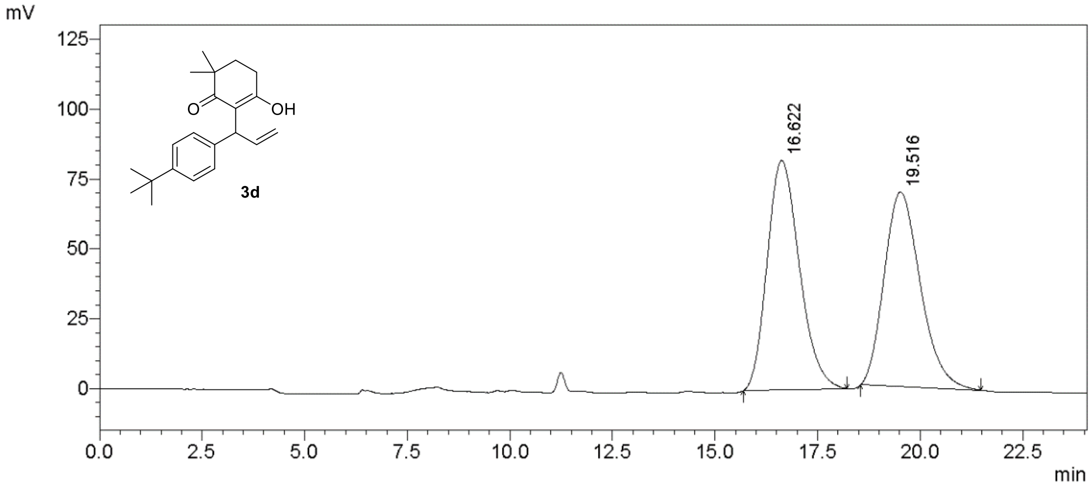

Peak No.	R. Time(min)	Peak Area(mV*min)	Percent (%)
1	16.622	4496572	51.216
2	19.516	4283118	48.784
**Total**		8779690	100.000

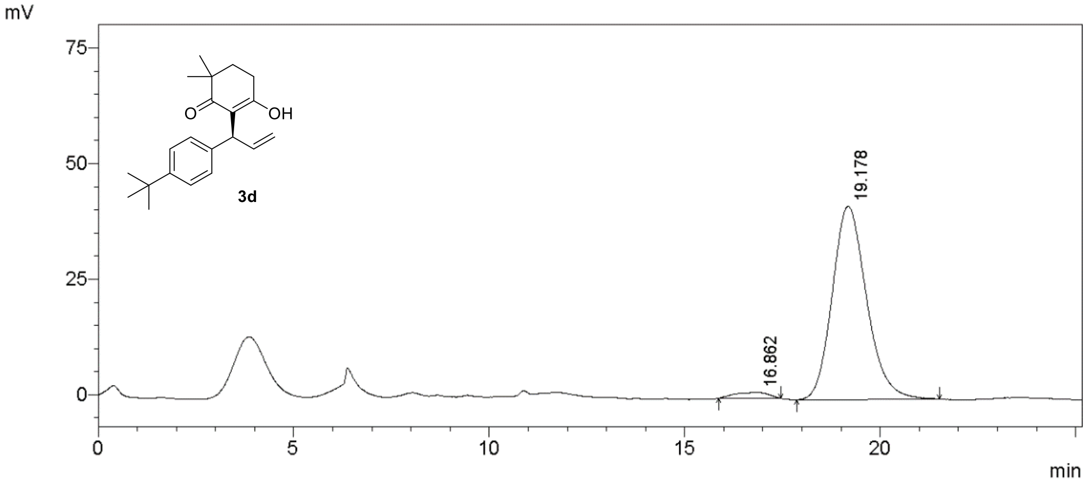

Peak No.	R. Time(min)	Peak Area(mV*min)	Percent (%)
1	16.862	75612	2.875
2	19.178	2553918	97.125
**Total**		2629530	100.000

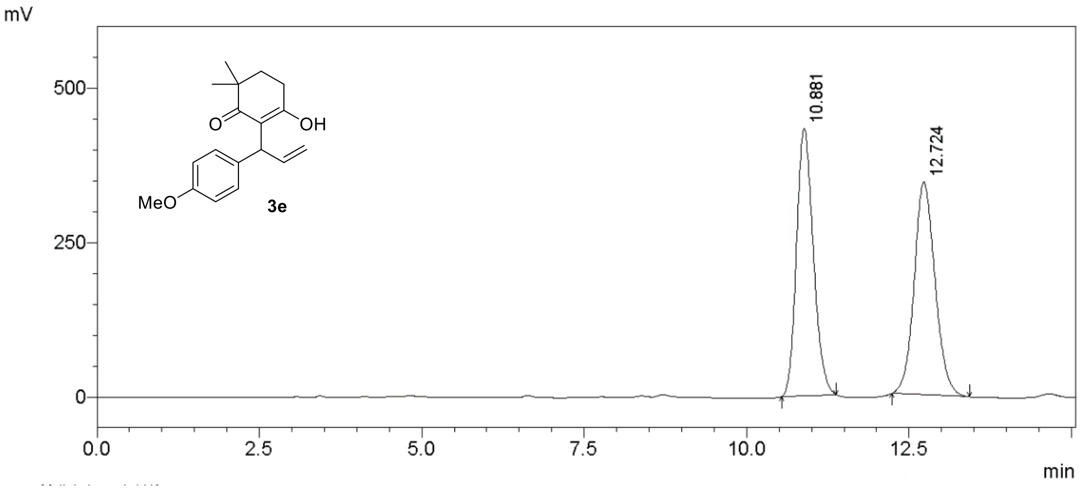

Peak No.	R. Time(min)	Peak Area(mV*min)	Percent (%)
1	10.881	7784212	50.236
2	12.724	7710951	49.764
**Total**		15495163	100.000

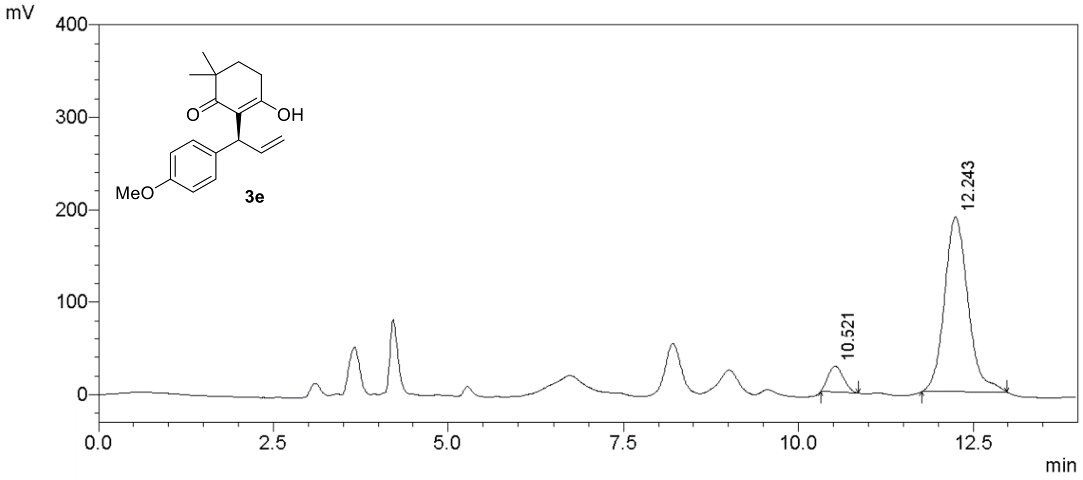

Peak No.	R. Time(min)	Peak Area(mV*min)	Percent (%)
1	10.521	427376	8.799
2	12.243	4429667	91.201
**Total**		4857043	100.000

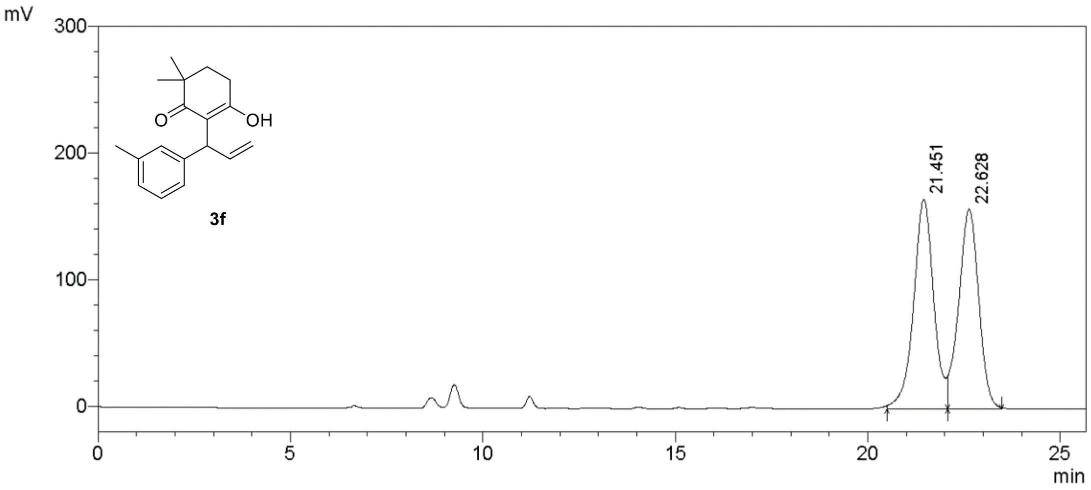

Peak No.	R. Time(min)	Peak Area(mV*min)	Percent (%)
1	21.451	6037062	51.478
2	22.628	5690366	48.522
**Total**		11727427	100.000

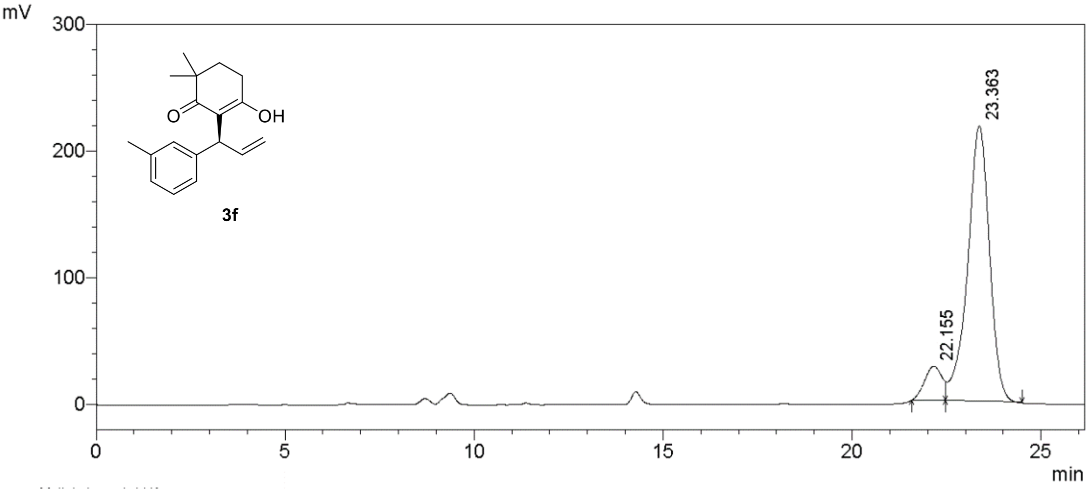

Peak No.	R. Time(min)	Peak Area(mV*min)	Percent (%)
1	22.155	858654	8.949
2	23.363	8736033	91.051
**Total**		9594687	100.000

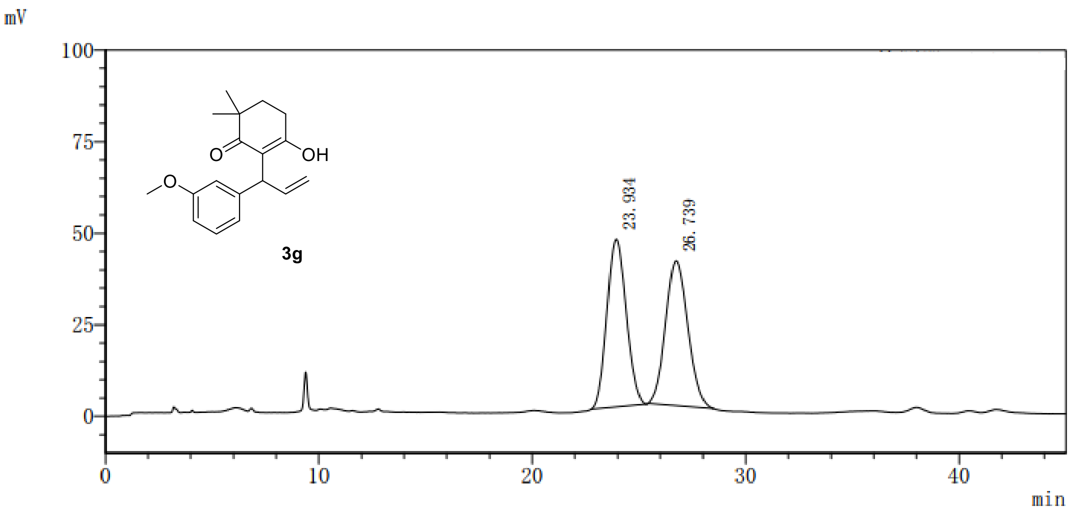

Peak No.	R. Time(min)	Peak Area(mV*min)	Percent (%)
1	23.934	2879674	50.095
2	26.739	2868756	49.905
**Total**		5748430	100.000

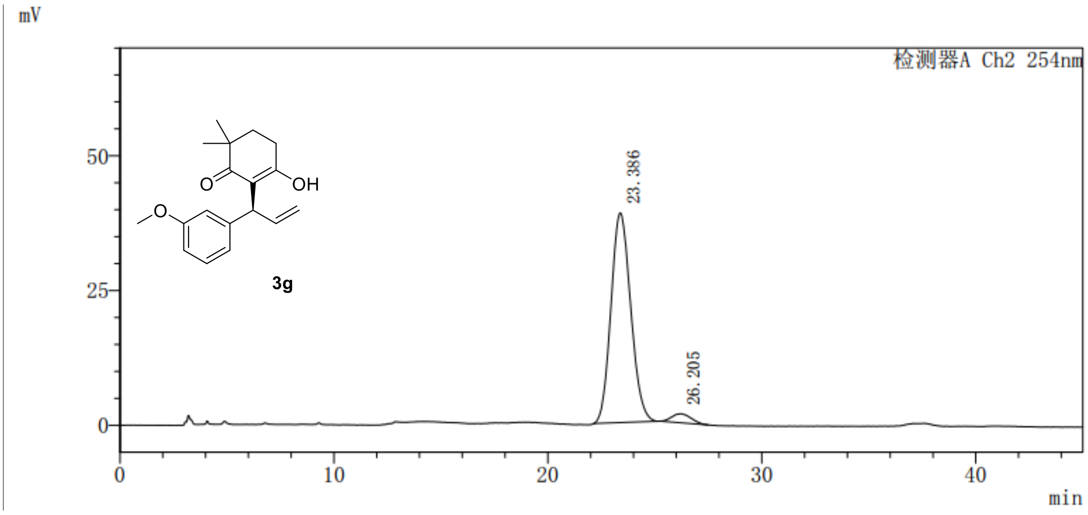

Peak No.	R. Time(min)	Peak Area(mV*min)	Percent (%)
1	23.386	2492475	96.005
2	26.205	103720	3.995
**Total**		2596195	100.000

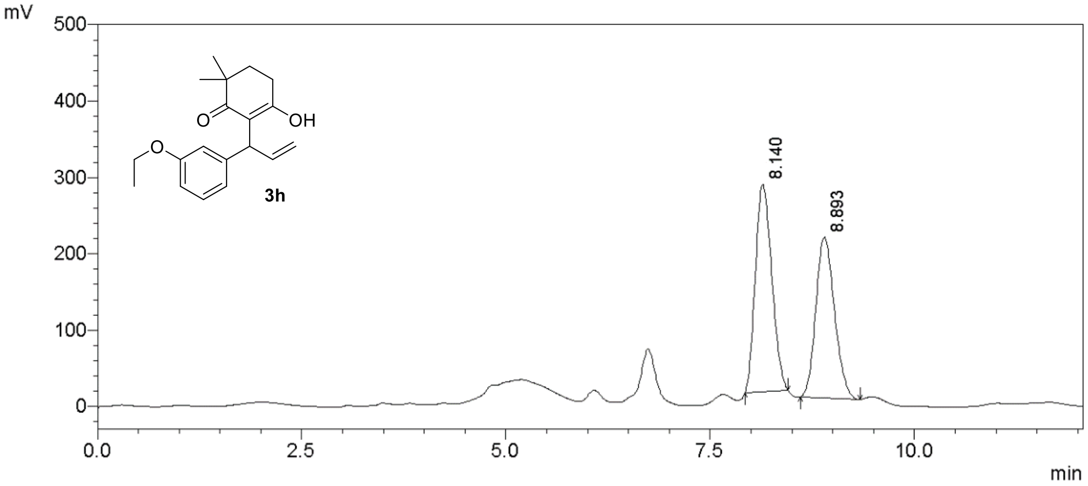

Peak No.	R. Time(min)	Peak Area(mV*min)	Percent (%)
1	8.136	4261033	51.901
2	8.892	3948929	48.099
**Total**		9594687	100.000

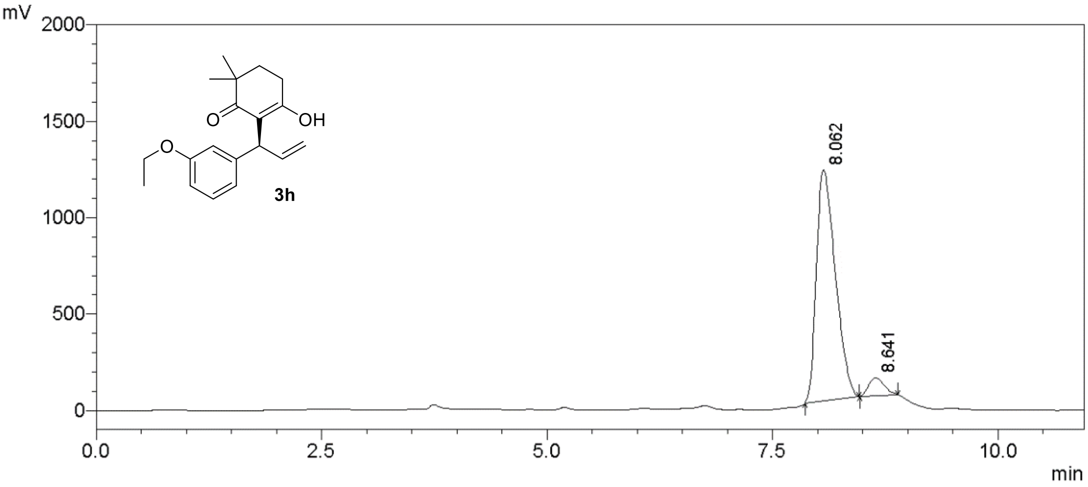

Peak No.	R. Time(min)	Peak Area(mV*min)	Percent (%)
1	8.082	17516935	94.935
2	8.641	1111160	5.065
**Total**		18628095	100.000

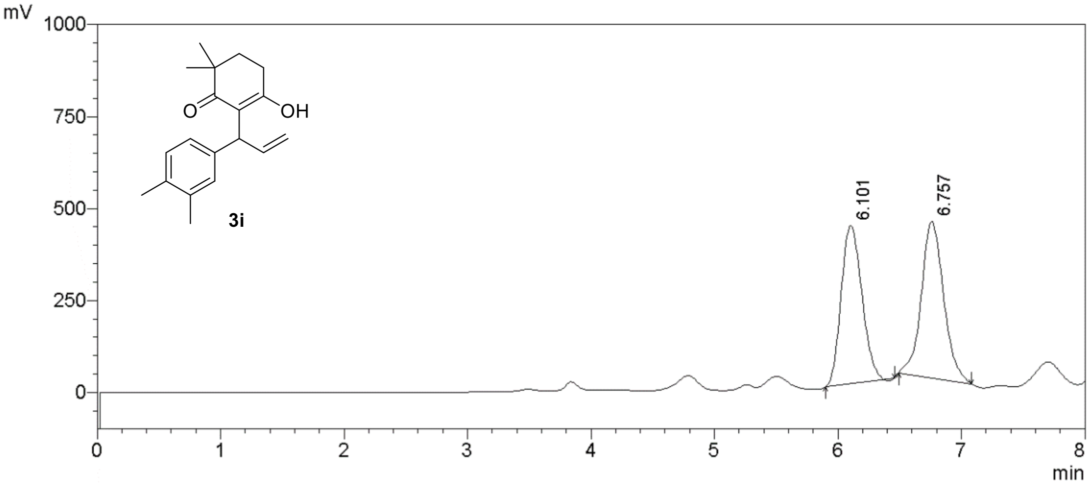

Peak No.	R. Time(min)	Peak Area(mV*min)	Percent (%)
1	6.098	5948449	47.420
2	6.756	6595691	52.580
**Total**		12544141	100.000

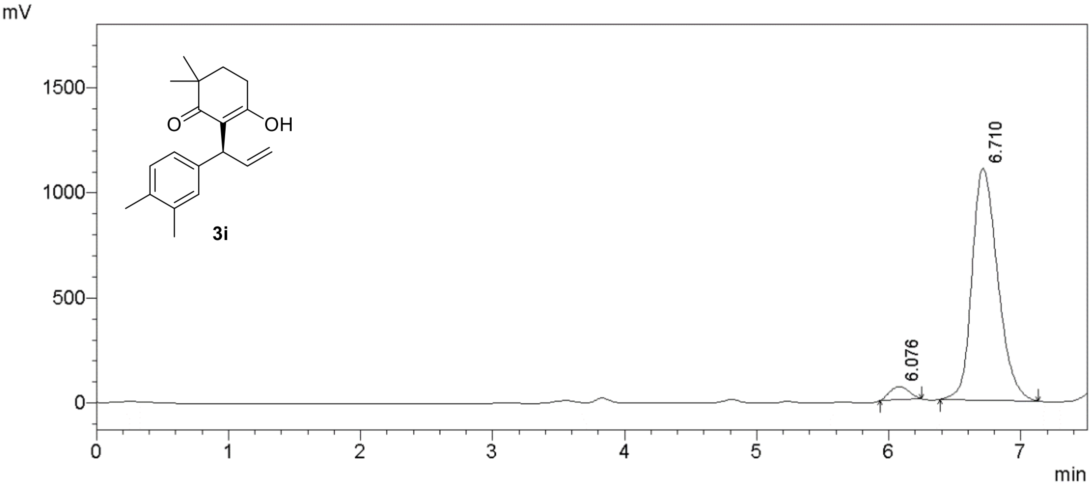

Peak No.	R. Time(min)	Peak Area(mV*min)	Percent (%)
1	6.073	765687	4.165
2	6.708	17618645	95.835
**Total**		18384332	100.000

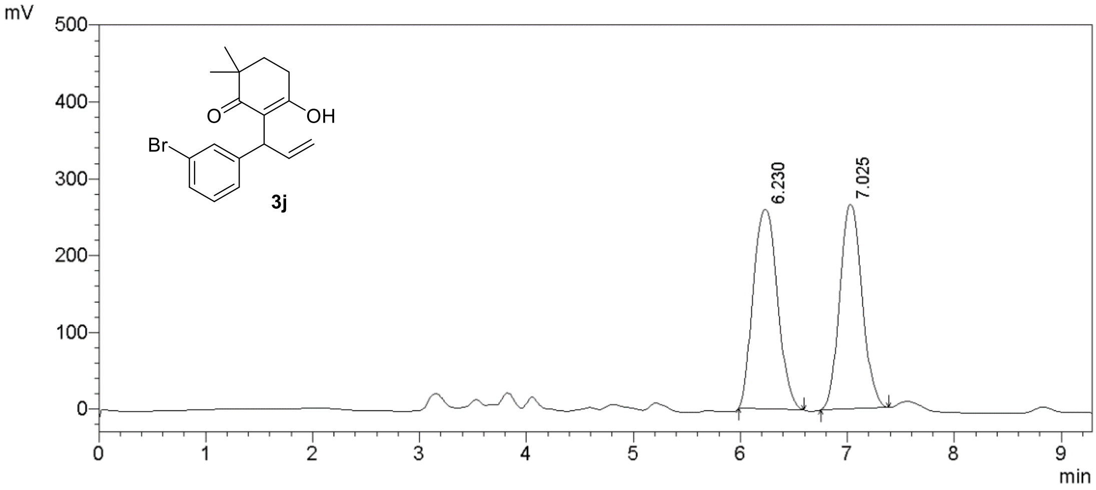

Peak No.	R. Time(min)	Peak Area(mV*min)	Percent (%)
1	6.232	3728664	51.712
2	7.027	3481768	48.288
**Total**		7210432	100.000

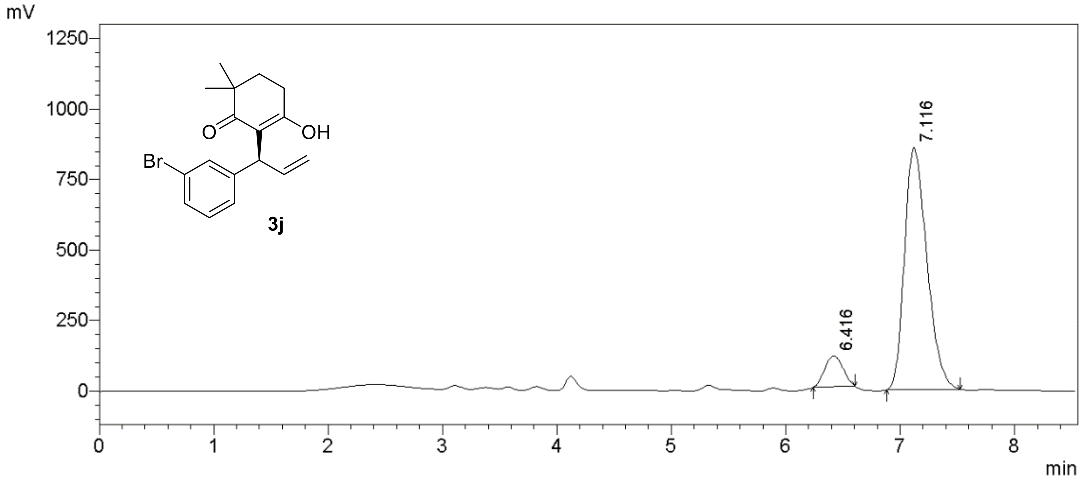

Peak No.	R. Time(min)	Peak Area(mV*min)	Percent (%)
1	6.416	1226910	9.495
2	7.116	11694158	90.505
**Total**		12921067	100.000

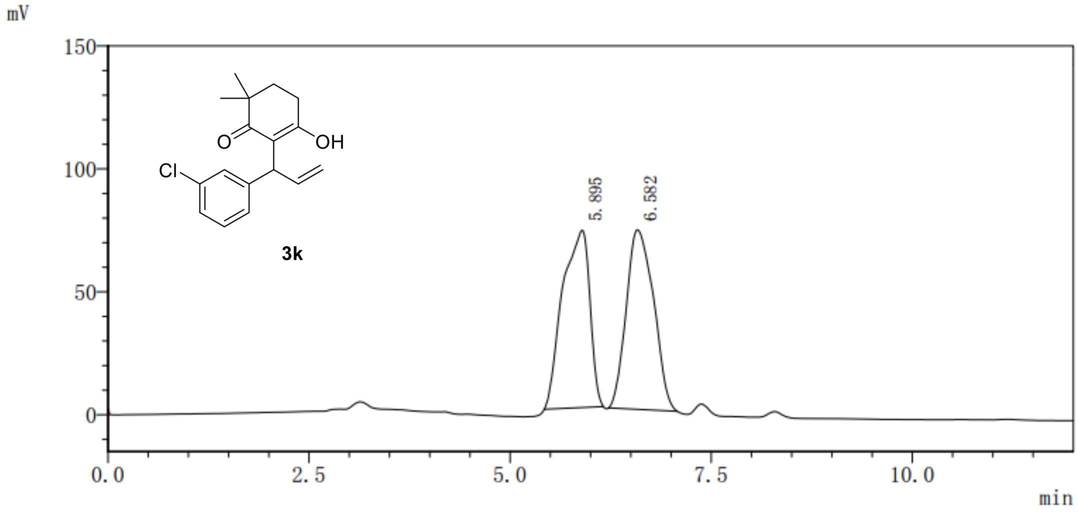

Peak No.	R. Time(min)	Peak Area(mV*min)	Percent (%)
1	5.895	1696141	49.532
2	6.582	1728214	50.468
**Total**		3424356	100.000

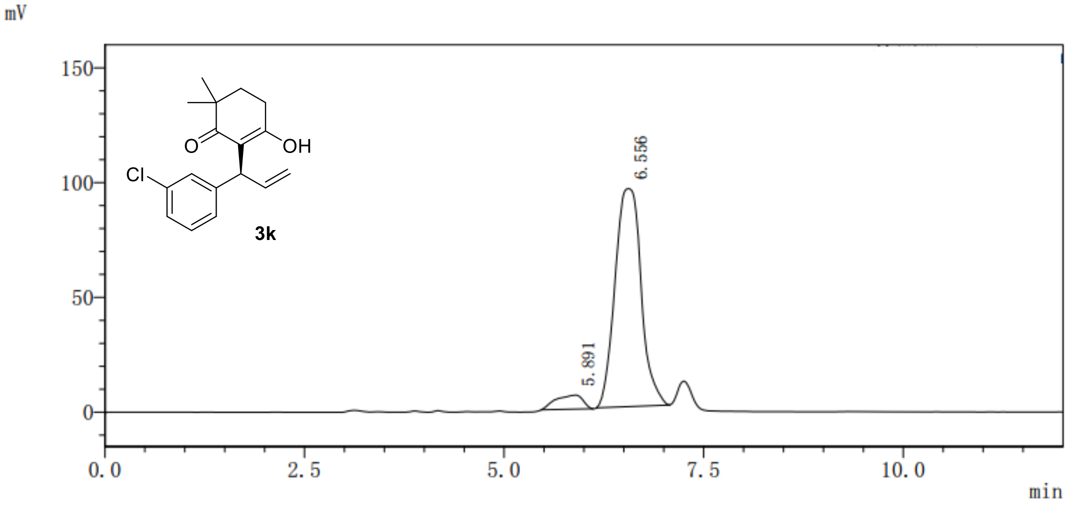

Peak No.	R. Time(min)	Peak Area(mV*min)	Percent (%)
1	5.891	145140	6.286
2	6.556	2163813	93.714
**Total**		2308953	100.000

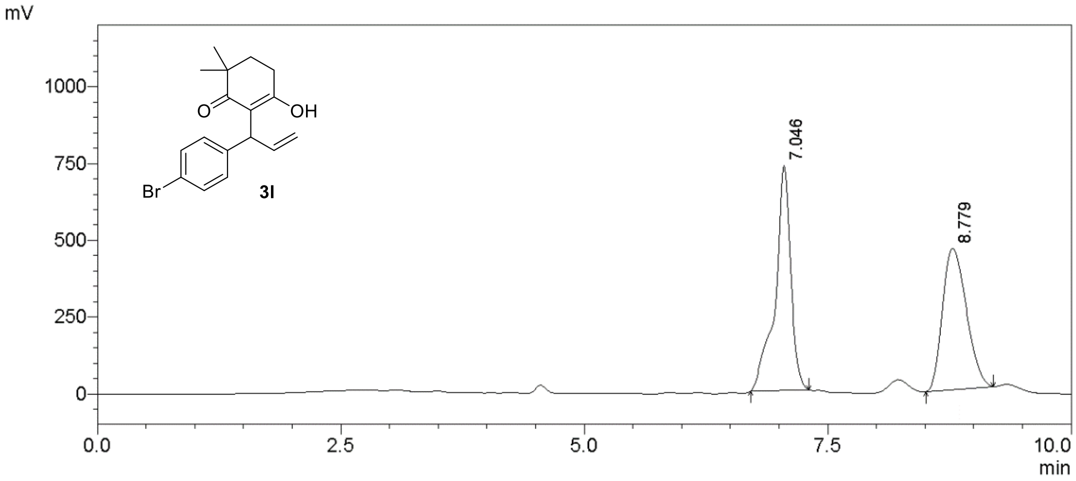

Peak No.	R. Time(min)	Peak Area(mV*min)	Percent (%)
1	7.046	8099342	50.965
2	8.779	7792659	49.035
**Total**		15892001	100.000

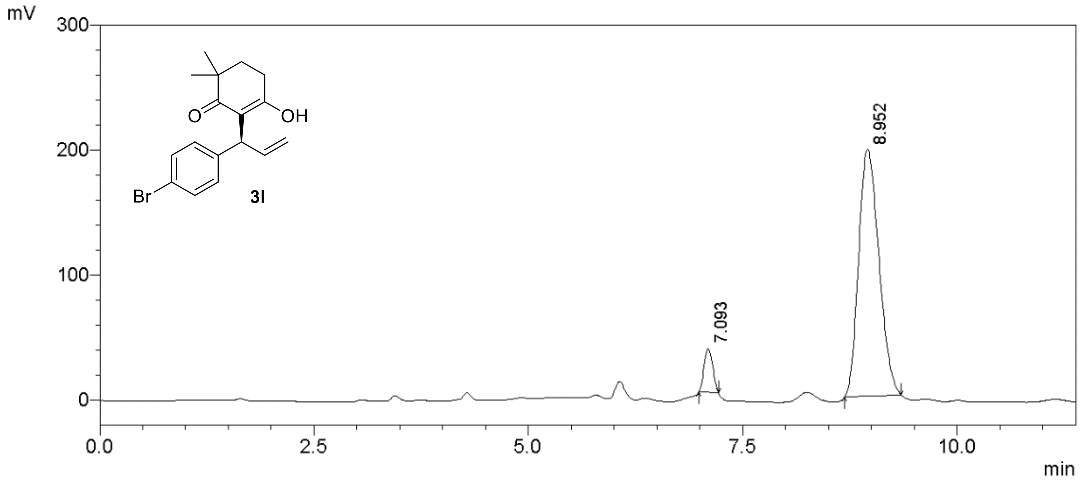

Peak No.	R. Time(min)	Peak Area(mV*min)	Percent (%)
1	7.093	241956	6.922
2	8.952	3253598	93.078
**Total**		3495553	100.000

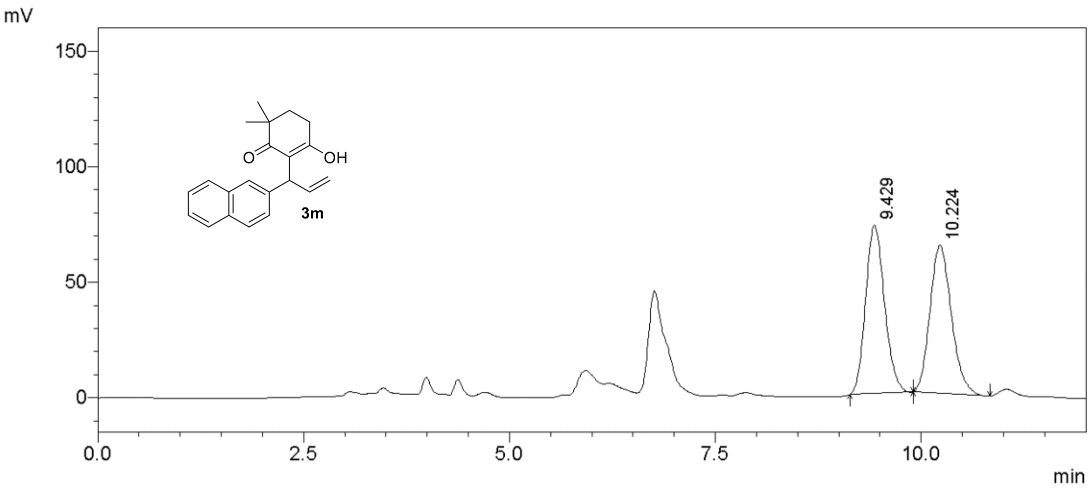

Peak No.	R. Time(min)	Peak Area(mV*min)	Percent (%)
1	9.429	1157171	50.040
2	10.224	1155316	49.960
**Total**		2312487	100.000

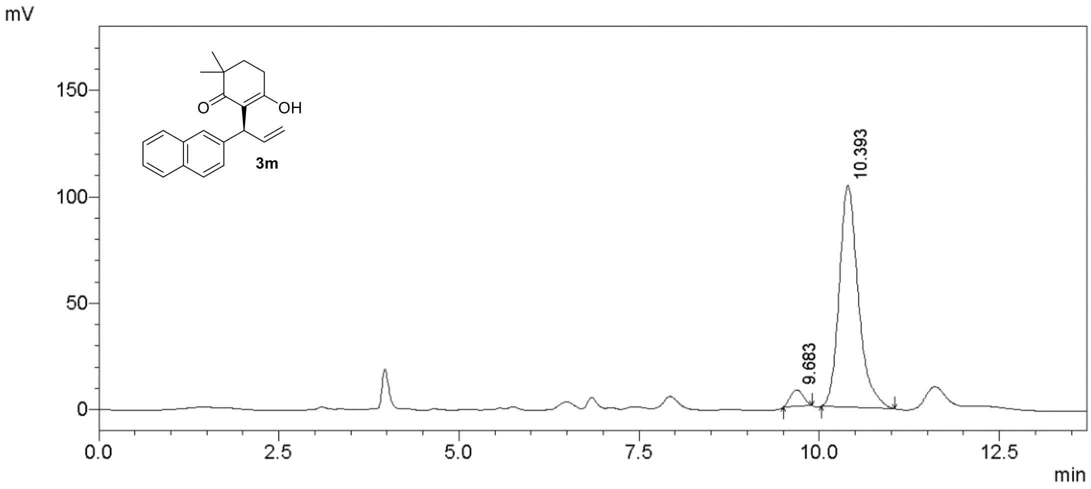

Peak No.	R. Time(min)	Peak Area(mV*min)	Percent (%)
1	9.683	98556	4.871
2	10.393	1924635	95.129
**Total**		2023191	100.000

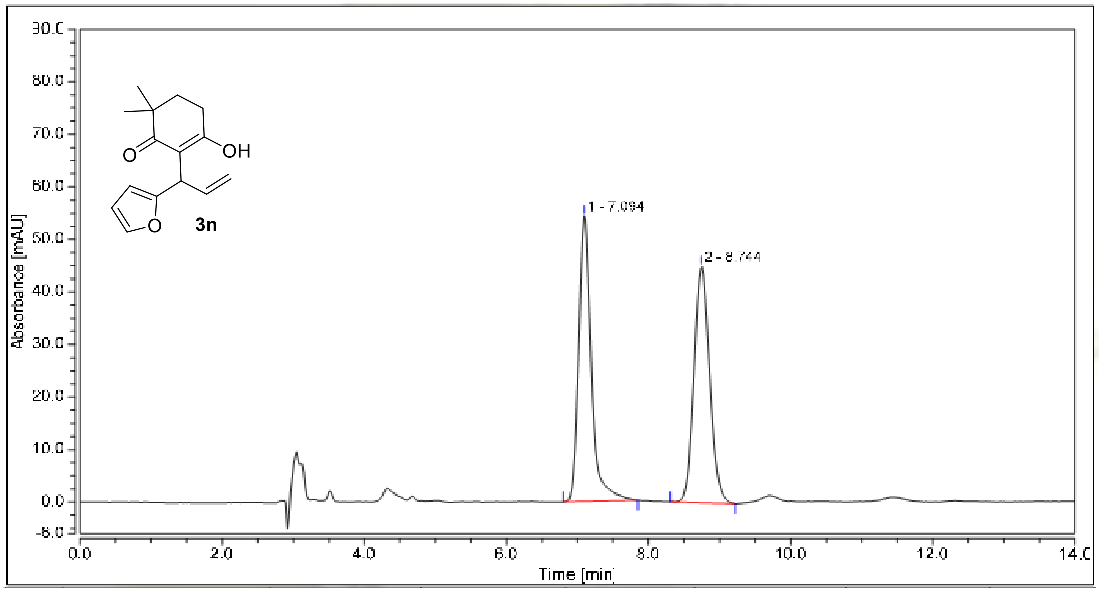

Peak No.	R. Time(min)	Peak Area (mAU*min)	Percent (%)
1	7.094	11.183	49.41
2	8.744	11.449	50.59
**Total**		22.633	100.000

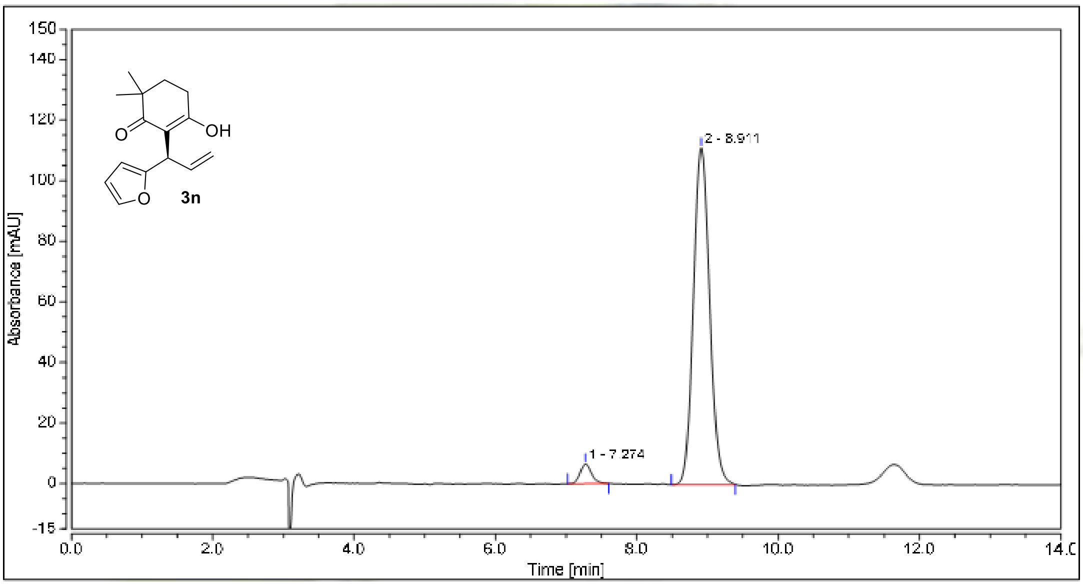

Peak No.	R. Time(min)	Peak Area (mAU*min)	Percent (%)
1	7.274	7006737	3.99
2	8.911	325365	96.01
**Total**		7332103	100.000

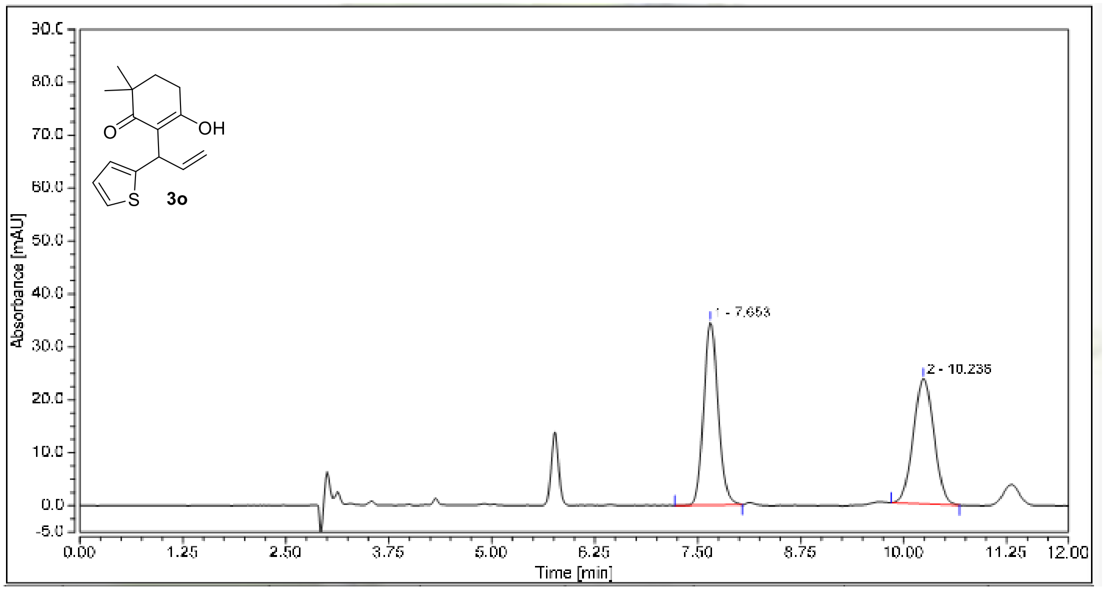

Peak No.	R. Time(min)	Peak Area (mAU*min)	Percent (%)
1	7.653	7.047	50.67
2	10.236	6.860	49.33
**Total**		13.907	100.000

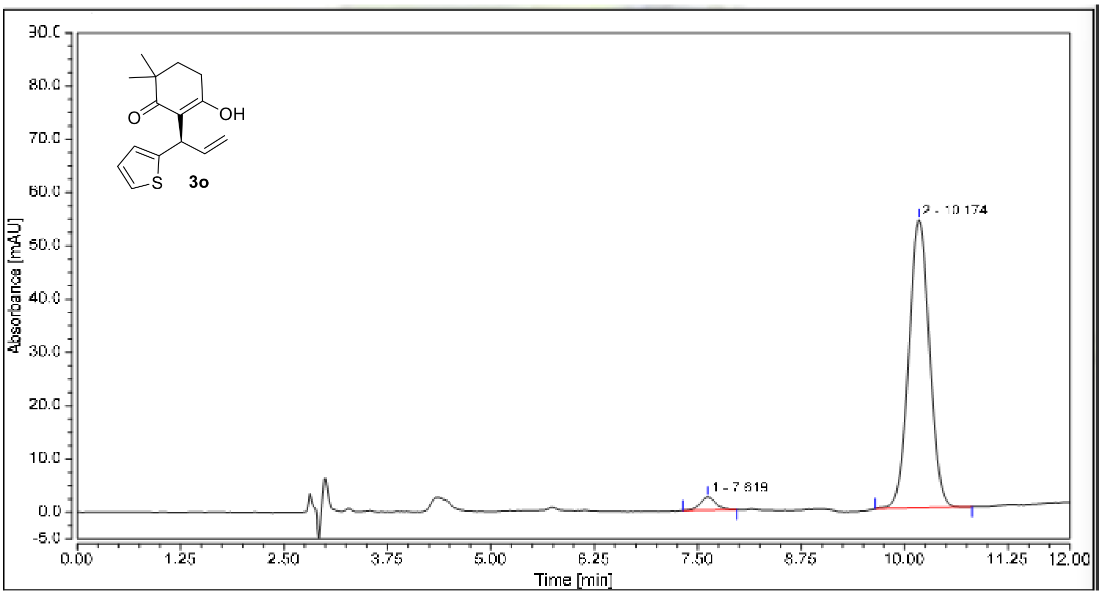

Peak No.	R. Time(min)	Peak Area (mAU*min)	Percent (%)
1	7.619	0.553	3.42
2	10.174	15.619	96.58
**Total**		16.172	100.000

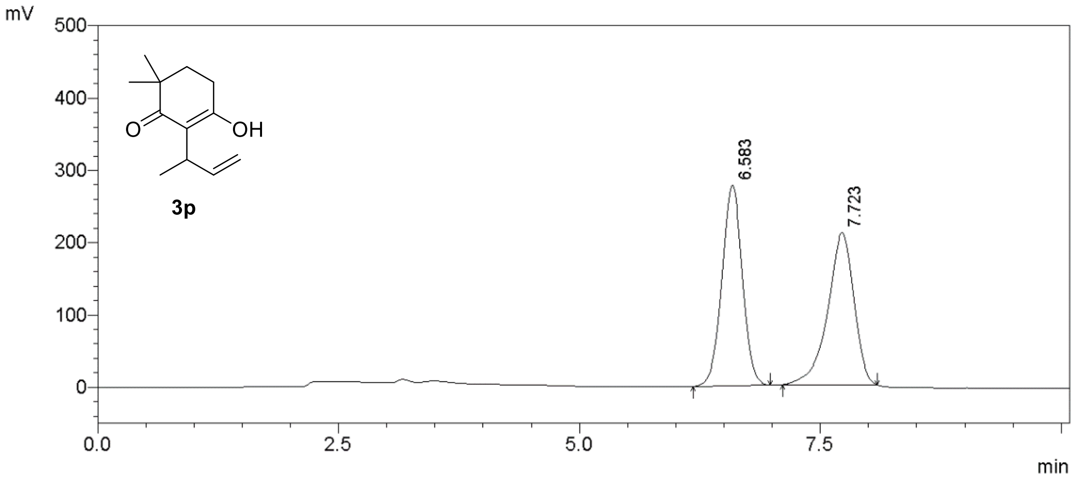

Peak No.	R. Time(min)	Peak Area(mV*min)	Percent (%)
1	6.583	4099851	50.677
2	7.723	3990381	49.323
**Total**		8090232	100.000

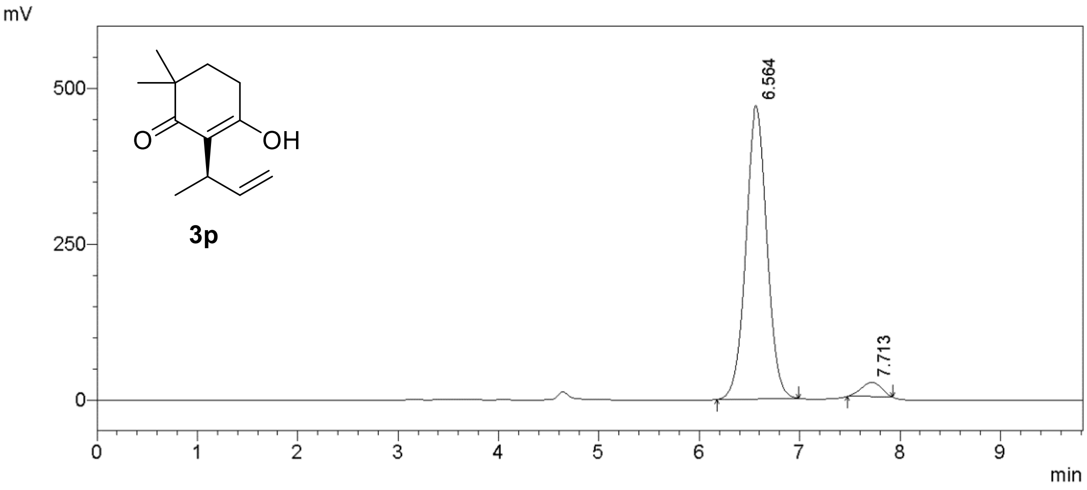

Peak No.	R. Time(min)	Peak Area(mV*min)	Percent (%)
1	6.564	7006737	95.562
2	7.713	325365	4.438
**Total**		7332103	100.000

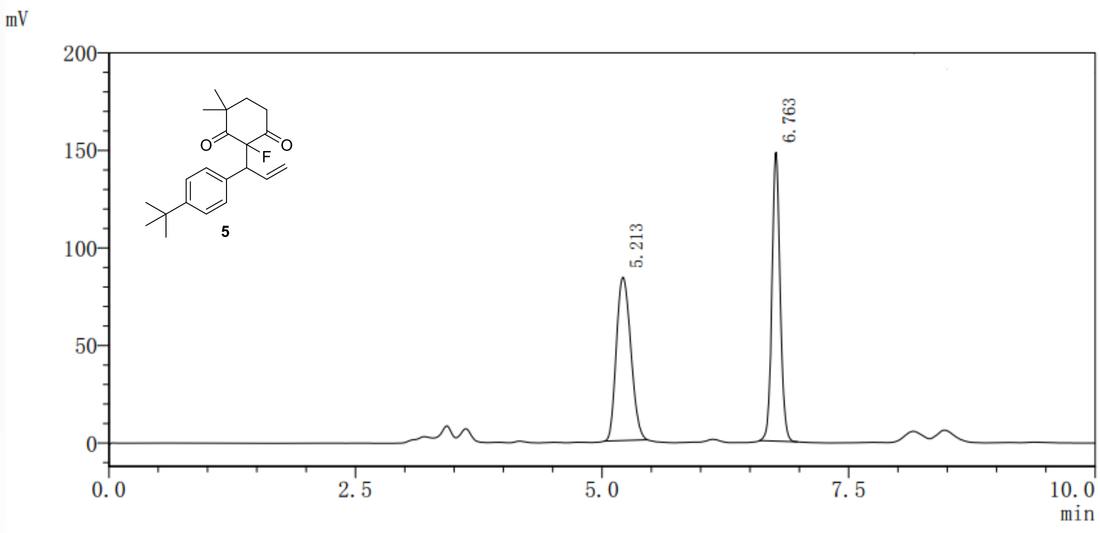

Peak No.	R. Time(min)	Peak Area(mV*min)	Percent(%)
1	5.213	849932	49.943
2	6.763	851888	50.057
**Total**		1701820	100.000

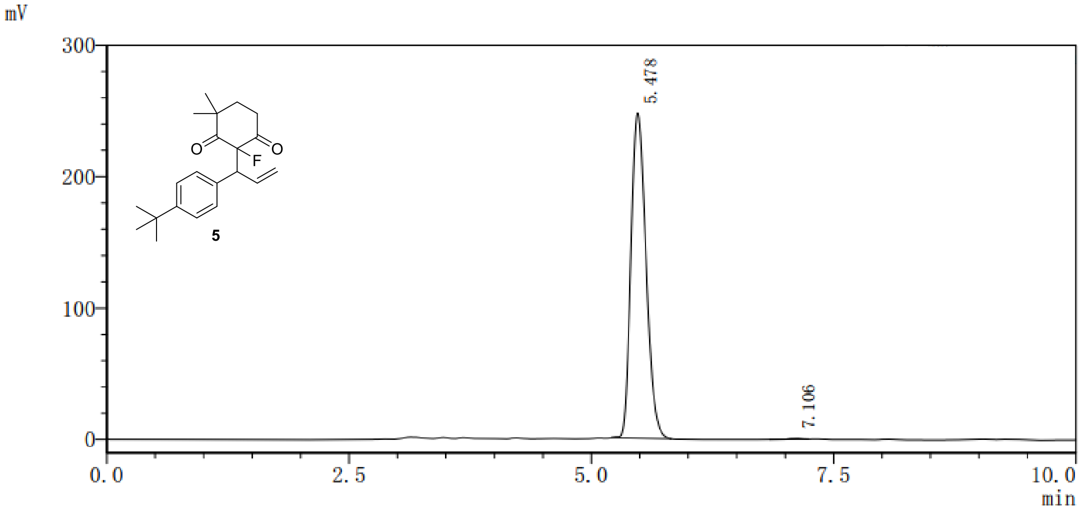

Peak No.	R. Time(min)	Peak Area(mV*min)	Percent(%)
1	5.478	2676604	99.814
2	7.106	4988	0.186
**Total**		2681592	100.000
